# A genome-wide investigation of the effect of farming and human-mediated introduction on the ubiquitous seaweed *Undaria pinnatifida*

**DOI:** 10.1038/s41559-020-01378-9

**Published:** 2021-01-25

**Authors:** Louis Graf, Younhee Shin, Ji Hyun Yang, Ji Won Choi, Il Ki Hwang, Wendy Nelson, Debashish Bhattacharya, Frédérique Viard, Hwan Su Yoon

**Affiliations:** 1 Department of Biological Sciences, Sungkyunkwan University, Suwon, Korea; 2 Aquaculture Management Division, National Institute of Fisheries Science, Busan, South Korea; 3 National Institute of Water & Atmospheric Research, Wellington, New Zealand; 4 School of Biological Sciences, University of Auckland, Auckland, New Zealand; 5 Department of Biochemistry and Microbiology, Rutgers University, New Brunswick, NJ USA; 6 Sorbonne Université, CNRS, AD2M, Station Biologique de Roscoff, Roscoff, France; 7 ISEM, Univ. Montpellier, CNRS, EPHE, IRD, Montpellier, France

**Keywords:** Ecology, Evolution, Genetics, Ecology

## Abstract

Human activity is an important driver of ecological and evolutionary change on our planet. In particular, domestication and biological introductions have important and long-lasting effects on species’ genomic architecture and diversity. However, genome-wide analysis of independent domestication and introduction events within a single species has not previously been performed. The Pacific kelp *Undaria pinnatifida* provides such an opportunity because it has been cultivated in its native range in Northeast Asia but also introduced to four other continents in the past 50 years. Here we present the results of a genome-wide analysis of natural, cultivated and introduced populations of *U. pinnatifida* to elucidate human-driven evolutionary change. We demonstrate that these three categories of origin can be distinguished at the genome level, reflecting the combined influence of neutral (demography and migration) and non-neutral (selection) processes.

## Abstract (web summary)

Human-driven evolutionary changes are captured by comparison of the genomes of natural, cultivated and introduced populations of a globally distributed Pacific kelp.

## Main

Unprecedented evolutionary experiments have resulted from the spread of humans on our planet. Initially considered anecdotal and rare^1^, human-driven evolutionary change is now reported at increasing rates^2,3^. These processes leave footprints in the genomes of many species that are cultivated^4,5^, domesticated^6,7^ or transported across different biogeographic regions (that is, biological introductions)^8,9^. In-depth examination and understanding of these changes is an important research area, because of their considerable ecological, economic and health implications^10^.

Domestication is a form of co-evolution between a species (that is, human) and another species it controls (in terms of growth and reproduction) for its own benefit. This process has shaped the evolution of hundreds of plants and animals^11,12^. As far back as 12,000 years ago, and the transition in human behaviour from food gathering to cultivation^13^, agricultural societies depended on the domestication and diversification of wild species, notably through selective breeding, hybridization or inbreeding^14,15^. As a result of the application of these methods, there was selection for so-called ‘domestication phenotypes’. These traits can arise through conscious selection (intentional choice made by humans of preferred phenotypes in cultivated species for use and propagation) or unintended selection (natural selection in crop species as a result of human cultivation practices in agro-ecological environments), challenging the clear discrimination of traits directly selected during domestication and other traits.

In a globalized environment, where borders are crossed legally and illegally every day by hundreds of thousands of humans and goods, plant and animal species are dispersed knowingly or unwittingly around the world. Since the end of the twentieth century, nonindigenous species have become a major concern in our societies^16,17^. These human-driven migrations bring into contact populations or species that have evolved in isolation in their respective native range, exacerbating evolutionary changes, notably through admixture and hybridization^18–20^, as well as by selective pressure on the introduced species in its new range^21^. Biological introductions thus represent a fascinating opportunity to understand major evolutionary processes, such as genotype by environment interactions^22^ or speciation dynamics^23^.

Only a handful of case studies offer the opportunity to simultaneously address human-driven evolutionary change due to domestication and introduction. The Pacific kelp *Undaria pinnatifida* (Harvey) Suringar (Laminariales, Phaeophyceae) provides such an opportunity. In its native range of Northeast Asia, this brown edible seaweed was exploited for centuries before being cultivated, and its farming represents 6.9% of worldwide seaweed production^24^. The transition from the harvesting of natural populations to the cultivation on ‘long lines’ happened during the 1950s^25^ with the development of seaweed cultivation techniques and their application to *U. pinnatifida* in Japan, then Korea and finally China^26^. During this period, farmers selected desired phenotypes and only recently were breeding techniques used to develop cultivars^26–28^. Parallel to cultivation, *U. pinnatifida* has been intentionally and unintentionally transported by humans across the planet. Since its first report outside its native range (that is, along the Mediterranean coast of France in the 1970s^29^), *U. pinnatifida* has become established along the coastlines of 14 countries across 4 continents^30–32^. This kelp presents the rare characteristic of being independently cultivated in its native range and introduced in four continents outside its native range. This situation contrasts with other well-studied cases such as *Oryza*^33^ and *Sorghum*^34^ in which domestication preceded escape to the wild. Recent studies have demonstrated the importance of genome-wide analyses, based on whole-genome sequencing data, to understand human-driven evolutionary change, notably with respect to domestication^4–7^, with fewer studies examining invasive species^8,9^, and none addressing both aspects at the same time. Here we report the genome sequence of a Korean cultivar of *U. pinnatifida*, sequenced independently from the genome sequence of the Chinese gametophyte^35^. On the basis of whole‐genome sequencing of multiple individuals sampled in native, cultivated and introduced populations, we compared genome architecture across these different categories. We argue that the differences observed among them probably result from the combined influence of demography and selection.

## Results and discussion

### The nuclear genome of *U. pinnatifida* Kr2015

**Fig. 1:**
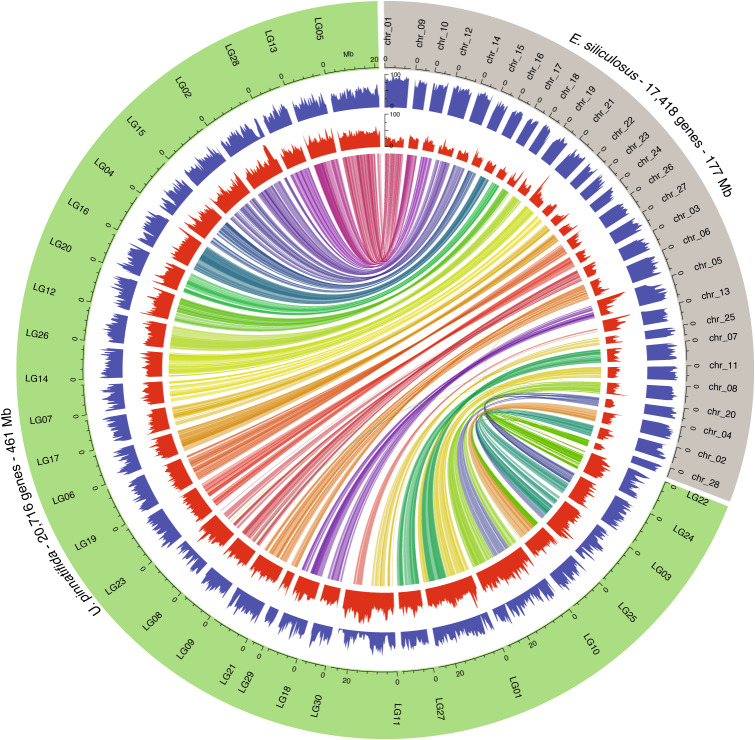
Pseudochromosome-level assembly of the *U. pinnatifida* genome and *E. siliculosus*. The outermost circle (blue) represents the density of genes in percentage of coverage in 500-kb windows. The middle circle (red) represents the density of repeated elements in percentage of coverage in 500-kb windows. The centre shows syntenic links between genes of the two species. The units (Mb) and tick marks on the axis for LG05 are common for all such axes.

We extracted genomic DNA from a cultivated *U. pinnatifida* sporophyte harvested in November 2015 in Wando, Korea and generated a nuclear genome assembly using PacBio long reads with ~100× sequence coverage (Supplementary Note and Supplementary Table 1). The assembled contigs were polished with ~32× coverage of Illumina paired-end reads. The resulting assembly consisted of 3,876 contigs with a total size of 634 megabases (Mb) with N50 (minimum contig length to cover 50 percent of the assembly) of 406 kb (Supplementary Figs. 1 and 2 and Supplementary Table 2). A genetic map^36^ was used to anchor and order 72.7% of the assembly (461 Mb; 1,325 contigs) into 30 linkage groups corresponding to the number of chromosomes in *U. pinnatifida*^37^ (Fig. 1, Supplementary Note, Supplementary Fig. 1 and Supplementary Tables 3–5). The genomes of *U. pinnatifida* from China^35^ and Kr2015 were largely comparable in length, composition and organization (Supplementary Note). Synteny analysis revealed discrepancies between the two assemblies that could represent recombination events or artefacts resulting from how these data were assembled (Supplementary Note and Supplementary Fig. 3). We also compared the Kr2015 genome to other brown algal genomes. The Kr2015 genome was annotated using a combination of transcript- and homology-based methods (Supplementary Note, Supplementary Fig. 4 and Supplementary Table 1). The annotation pipeline predicted 20,716 complete protein-coding genes, of which 78.25% were supported by transcriptome data (Supplementary Note).

The genome of *U. pinnatifida* Kr2015 is the largest reported thus far for brown algae. It is comprised of 52.1% (330.3 Mb) repeated elements, of which at least 19.14% are transposable elements, representing 121 Mb of the genome (Supplementary Note and Supplementary Table 6). Genomes of Laminariales are larger than those of Ectocarpales (for example, *Ectocarpus siliculosus*; see Supplementary Table 7). This genome expansion is driven by the differential rate of repeated element insertion (Supplementary Note and Supplementary Fig. 5). Insertion of repeated elements was homogeneous along the pseudochromosomes and resulted in a significantly (Wilcoxon rank sum test *P* value < 2.2 × 10^−16^) reduced gene density in Kr2015 when compared to *E. siliculosus* (Fig. 1). The traditional repeat-rich heterochromatin and gene-rich euchromatin could not be clearly differentiated in Kr2015 (refs. ^38–40^). Therefore, organization of brown algal chromosomes appears to be similar to that in fungi^41^, but different from that in plants^42^. This differential insertion of repeated elements does not appear to have disturbed the gene order: synteny is largely conserved between *U. pinnatifida* Kr2015 and *E. siliculosus* (Fig. 1). Despite the deep split of these two lineages 128.9–220.2 million years ago^43,44^, large chromosomal rearrangements are rare. Overall, 16 *U. pinnatifida* Kr2015 pseudochromosomes share synteny with one chromosome from *E. siliculosus* (Fig. 1 and Supplementary Note). The chromosome number discrepancy between *E. siliculosus* (28 chromosomes) and *U. pinnatifida* (30 chromosomes) may be explained by 4 splitting and fusion events involving 5 and 7 chromosomes, respectively (Fig. 1).

The gene inventory of *U. pinnatifida* is largely shared with other brown algae; however, the Laminariales common ancestor contains expanded gene families that encode nuclear-targeted proteins with transcription regulation functions (Supplementary Note). This suggests that following the split with Ectocarpales, the Laminariales may have evolved a more sophisticated control of gene expression (Supplementary Note).

### Genome polymorphism across individuals

**Fig. 2:**
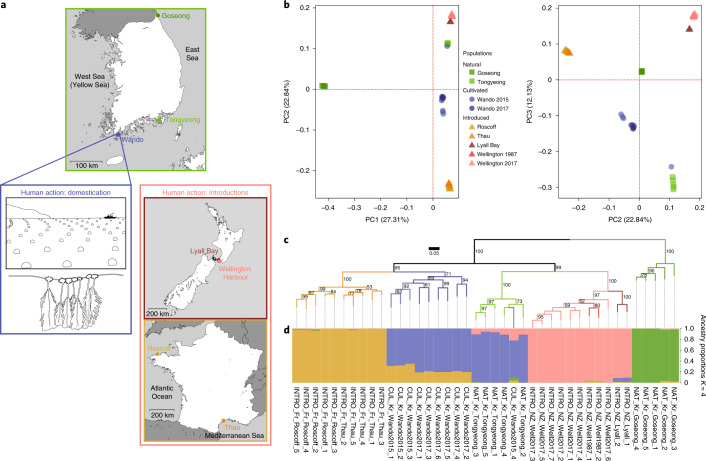
Genetic structure according to geographic locations. **a**, The sampling strategy for the study of the impact of human activities on the genome architecture and diversity of *U. pinnatifida*. Green box, natural populations from the native range: Goseong (Korea) and Tongyeong (Korea). Blue box, cultivated populations from the Wando (Korea) farming area harvested in 2015 and 2017. Red box, natural populations introduced in New Zealand and France, outside the native range: Lyall Bay (New Zealand), Thau (France), Roscoff (France) and Wellington Harbour (New Zealand) sampled in 1987 and 2017. **b**, PCA of 7,253,541 variants called in 41 individuals of *U. pinnatifida*. The left plot shows PC1 versus PC2; the right plot shows PC2 versus PC3. **c**, Maximum-likelihood phylogenetic tree reconstruction of the 9,777 high-quality SNPs shared by all 41 individuals. The support values shown near the nodes were estimated by 1,000 parametric bootstrap replications. A blue background highlights Korean populations, a red background highlights French populations and a green background highlights New Zealand populations. **d**, Admixture analysis showing the membership (ancestry proportion) to five identified clusters (*K* = 5) that best explained the overall genetic variance of the dataset.

We resequenced the genomes of 41 individuals of *U*. *pinnatifida* from 9 populations in 3 categories: 2 natural kelp beds; 2 cultivated populations from the native range; and 5 introduced populations from France and New Zealand (Fig. 2a). We generated a total of 853.77 Gb of cleaned–trimmed paired-end sequence from the 41 individuals (average 20.69 Gb per individual). These reads were mapped to the reference genome assembly of *U*. *pinnatifida*. We obtained an average sequencing depth of 30.67× and average genome coverage of 94.77% (Supplementary Table 8). Using GATK^45^ to call variants, we identified 6,123,124 high-quality single-nucleotide polymorphisms (SNPs) and 1,130,417 high-quality insertions or deletions (indels) shared across the 9 populations (Supplementary Note and Supplementary Fig. 6). A large proportion of the variants were found in intergenic regions (53.81%) and only 3.07% were present in exons (Supplementary Fig. 7).

**Extended Data Fig. 1:**
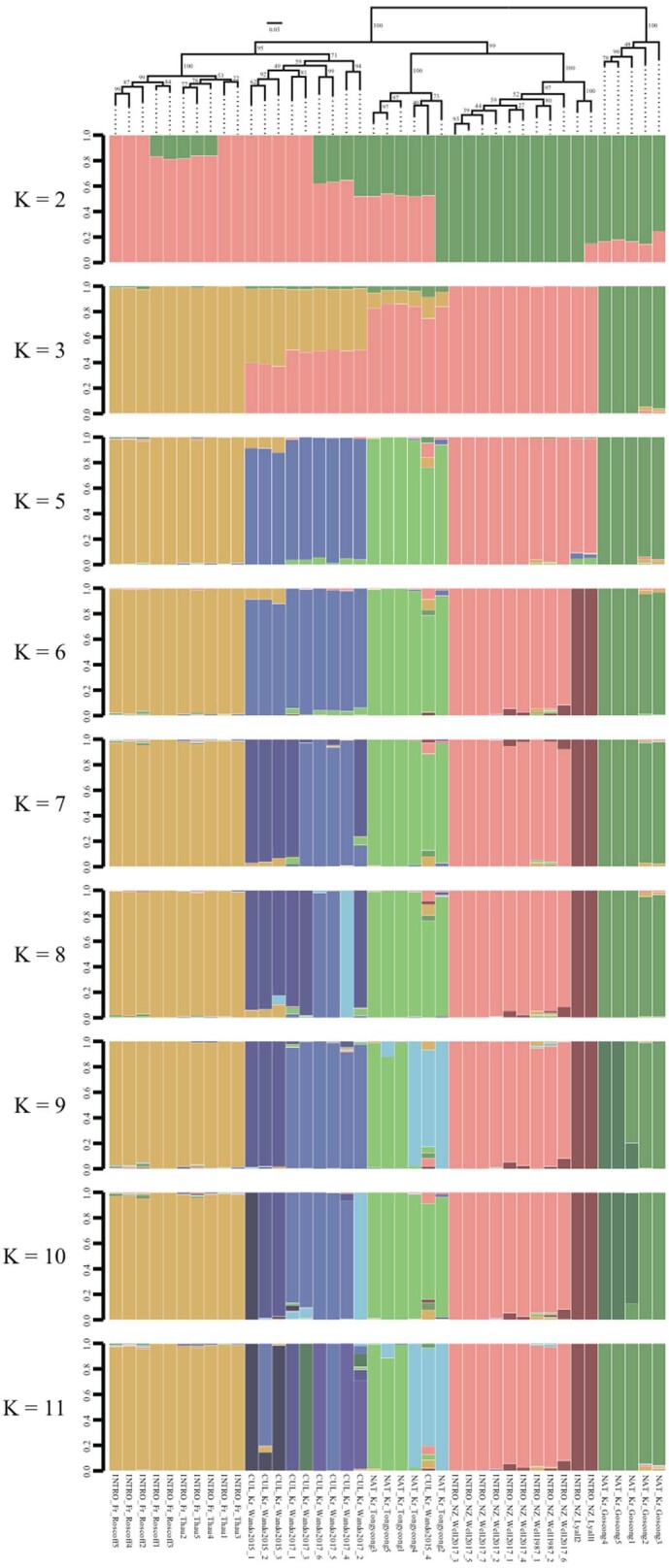
Admixture analysis. Ancestry proportion obtained with *snmf* for number of clusters K ranging from 2 to 11. The phylogenetic tree of Fig. 2c is reproduced on top.

The nuclear variant data were used to explore genetic diversity among *U*. *pinnatifida* individuals. Both principal component analysis (PCA) and phylogenetic reconstruction revealed a clear segregation of individuals according to their geographic locations (Fig. 2b,c). The admixture analysis performed with the R package LEA^46^ revealed consistent clusters for the number of groups (*K*) best explaining the genetic variance (*K* = 4 and 5; Extended Data Fig. 1, Supplementary Note and Supplementary Fig. 8). Only the CUL_Kr_Wando2015_4 individual was inconsistent with geographic clustering because it grouped with the Tongyeong individuals in the PCA analysis and the phylogenetic tree (Fig. 2b,c). This could be the outcome of either introgression between natural and cultivated populations, or cryptic genetic diversity within the cultivated accession (not detected here because of the limited number of individuals studied). As it had the highest level of admixture with a large number of SNPs/indels shared with Tongyeong individuals (Fig. 2d and Extended Data Fig. 1), admixture analysis supports the introgression hypothesis. This singular individual was excluded from subsequent analyses.

Time (albeit of short duration) appears to have little to no influence, because populations remain stable over time. For instance, the individuals sampled in 1987 and 2017 in the introduced population of Wellington (New Zealand) were indistinguishable in the three analyses (Fig. 2b–d and Extended Data Fig. 1). Similar observations based on double-digest restriction-site-associated DNA sequencing have been made over about 20 generations in populations introduced in France^47^. We examined two introduction ranges, which had been previously reported to have distinct introduction histories^48,49^. Our data are in agreement with these reports: the 21 introduced individuals formed 2 distinct clusters, corresponding to France and New Zealand. The clustering of the Lyall Bay and Wellington Harbour populations was consistent with local spreading by human vectors (for example, leisure boating), as observed in France^47^.

Overall, population structure analysis shows that the study populations do not present a cryptic substructure, confirms the temporal genetic stability of *U. pinnatifida*^48^ and marks the French and New Zealand introductions as two independent examples of how human activity has impacted the *U. pinnatifida* genome.

### Genomic landscape based on place of origin

**Fig. 3:**
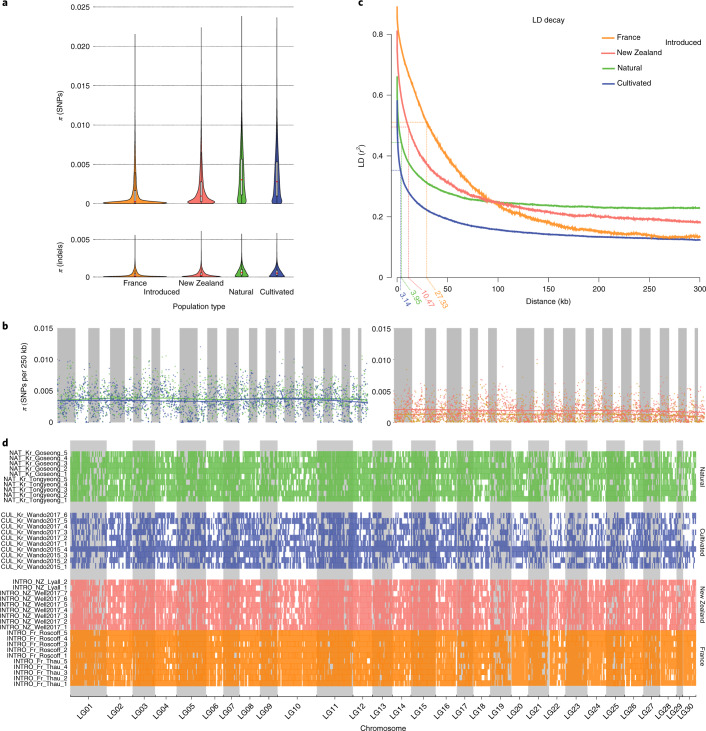
Genomic landscape in different population types. **a**, Violin plot of genetic diversity estimated using *π* in non-overlapping 10-kb windows. **b**, Manhattan plot of genetic diversity (*π*) estimated in 250-kb windows for the natural (green), cultivated (blue), New Zealand (red) and French (yellow) populations. Local polynomial regression fittings are shown on the plots. **c**, LD decay in the four different types of population, with the thin line indicating the distance at which LD is one-half of its maximum. **d**, ROH in the 41 individuals. Natural, cultivated, New Zealand and French are shown in green, blue, red and yellow, respectively.

To further explore the genome-wide impact of human activity, we characterized the genomic landscape in the different populations. Natural populations were characterized by high genetic diversity (mean *π* = 0.0044; Fig. 3a,b, Extended Data Fig. 1, Supplementary Fig. 9 and Supplementary Table 9) and high recombination rates (linkage disequilibrium (LD) half-maximum decay at 3.95 kb in natural; Fig. 3c) but relatively high homozygosity (natural mean total runs of homozygosity (ROH) length = 80.9 Mb and average ROH length = 1.13 Mb; Fig. 3d and Supplementary Table 10). Compared to natural populations, cultivated and introduced populations display contrasting features regarding their genomic landscape, for population diversity, LD and ROH. We might have expected that both cultivation and introduction processes would lead to a reduction in diversity through demographic bottleneck/founder events, associated with the introduction or the selection of few individuals; however, we did not observe such a pattern. Indeed, introduced populations of *U. pinnatifida* behaved as expected with low genetic diversity (France mean *π* = 0.0015; New Zealand mean *π* = 0.0022; Fig. 3a,b, Extended Data Fig. 1, Supplementary Fig. 9 and Supplementary Table 9), low recombination rates (LD half-maximum decay at 10.47 kb in New Zealand and at 27.33 kb in France; Fig. 3c) and high levels of homozygosity (France mean total ROH length = 338.5 Mb and average ROH length = 1.79 Mb; New Zealand mean total ROH length = 201.2 Mb and average ROH length = 1.08 Mb; Fig. 3d and Supplementary Table 10). In contrast, cultivated populations were characterized by high genetic diversity (cultivated mean *π* = 0.0040; Fig. 3a,b, Extended Data Fig. 1, Supplementary Fig. 9 and Supplementary Table 9), high recombination rates (LD half-maximum decay at 3.14 kb in cultivated; Fig. 3c) and low homozygosity (cultivated mean total ROH length = 87.9.5 Mb and average ROH length = 0.96 Mb; Fig. 3d and Supplementary Table 10).

#### Influence of introduction history on the genomic landscape

When compared to those of populations from its native range, the characteristics of the French and New Zealand *U. pinnatifida* populations probably reflect founder events, whereby a small number of individuals were introduced to the new habitat. Such founder events are uncommon in marine introduced species, when compared to their terrestrial counterparts^20^. However, we have evidence of such a founder effect that is known to reduce genetic diversity and the rate of LD decay, and to increase inbreeding (here, ROH), particularly in selfing species such as *U. pinnatifida*^47^.

However, additional comparisons between the French and New Zealand introduced populations revealed different patterns between the two regions. The two French populations display a lower genetic diversity (Fig. 3a,b) and a higher LD (Fig. 3b) than the New Zealand populations. The populations introduced to New Zealand waters display properties closer to those observed in the natural population in Korea than in the populations introduced to France. These features are in agreement with the supposed introduction history and vectors in these two regions. On the basis of field and genetic studies^47–51^, it has been hypothesized that the introduction occurred as a result of aquaculture in France and shipping in New Zealand. In France, *U. pinnatifida* would have been first introduced in the Thau Lagoon (Thau population here) with Pacific oyster imports from Asia, and then transported to Brittany (that is, the Roscoff population). Two sequential founder events thus probably occurred, from the same source in the native range^49^. Conversely, repeated introductions probably occurred with shipping in New Zealand, leading to a moderate decrease in genetic diversity when compared to the native range and the French populations, as found using mitochondrial haplotype analysis^49^. This scenario was further supported by individuals sampled in Wellington in 1987 at the time of the first report of this alga in New Zealand^52^. These seaweeds had a genetic diversity that was slightly higher than in the French population (Supplementary Fig. 10a and Supplementary Table 9), and the length of the ROH was shorter than in the French population (Supplementary Fig. 10b). Finally, the decrease of ROH length in 30 years (about 60 generations) suggests that repetitive introductions provided the potential for admixture in New Zealand.

#### Effect of cultivation on the genomic landscape

**Extended Data Fig. 2:**
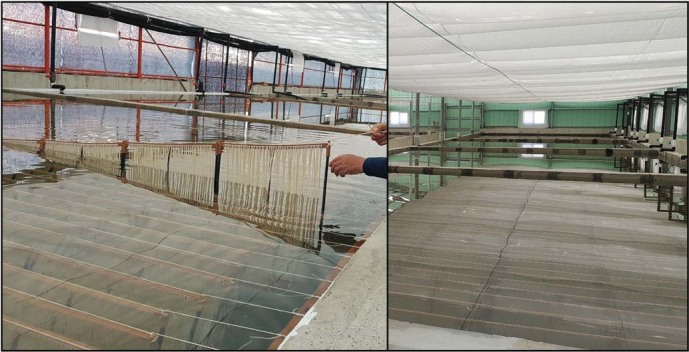
Large scale cultivation facility of *Undaria pinnatifida* in Korea. Indoors pools where multiple sporangia of *Undaria pinnatifida* are placed to enable recruitment on culture ropes (left).

**Extended Data Fig. 3:**
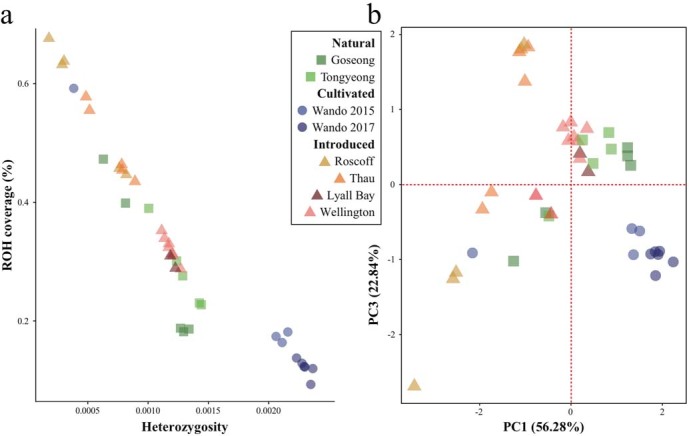
Heterozygosity and run of homozygosity. (**a**) Average genome-wide level of heterozygosity in the individuals as a function of the coverage of run of Homozygosity (ROH) in the genomes of the 41 individuals. (**b**) Principal Component Analysis (PCA) calculated on the number of ROH, total length of ROH and average length of ROH of each of the 41 individuals.

The cultivation process, through selective breeding, is expected to create a genetic bottleneck, resulting in cultivars that have low diversity and a suppressed recombination rate. The cultivated populations of *U. pinnatifida* in Korea, however, deviated from these predictions, with genetic diversity (mean *π* = 0.0040; Supplementary Table 9) and LD disequilibrium decay (LD half-maximum decay at 3.14 kb; Fig. 3c) comparable to those of natural populations (Fig. 3a–c). Interestingly, in France, cultivated populations of *U. pinnatifida* have a genetic diversity that is lower than that of natural populations (for example, twofold to threefold lower^47^) in accordance with expectations of the cultivation process (with some exceptions^53^). This observed discrepancy might be explained by the difference in the scale of the cultivation in these two countries. In France, *U. pinnatifida* cultivation remains limited to a few farms, whereas in Korea, its culture averages 0.5 million wet weight tonnes annually^24–26^. Owing to this large scale, the fertilization of culture ropes is carried out in large indoor pools in which multiple sporangia of individuals with valued phenotypes are placed together (Extended Data Fig. 2). Frequently, individuals from different natural populations are mixed with cultivated individuals from previous years. In this artificial environment, the mixing of genetically distinct zoospores is favoured, resulting in the observed high diversity and low LD; both of which are comparable to those of native natural populations. Furthermore, this cultivation methodology prevents the naturally high selfing rate of natural *U. pinnatifida* populations^47^. This selfing rate probably results from the low motility and short life span of the zoospores of this species^54,55^, traits that are mitigated by the large fertilization pool present in the water circulation system. This results in cultivated individuals having the highest level of heterozygosity and the lowest coverage of ROH in our study, even lower than in natural populations. Furthermore, these low values are consistent across all cultivated individuals (Fig. 3d and Extended Data Fig. 3), supporting the idea that they are a consequence of farming practices, the breeding method in particular.

This unexpected genomic landscape resulting from large-scale cultivation of *U. pinnatifida* could be of great interest for conservation biologists. *U. pinnatifida* populations have been declining in its native range^56^, as have kelp forests globally^57^. It is therefore clear that conservation efforts are needed to protect these valuable marine species. In this regard, our analysis suggests that if kelp cultivation is designed to maintain high genetic variation, then farmed individuals could act as reservoirs of evolutionary potential. *U. pinnatifida* provides a model for such an approach. Such genetic rescue approaches have previously been used in allogamous mammals^58,59^.

### Catalogue of regions under putative selection

**Fig. 4:**
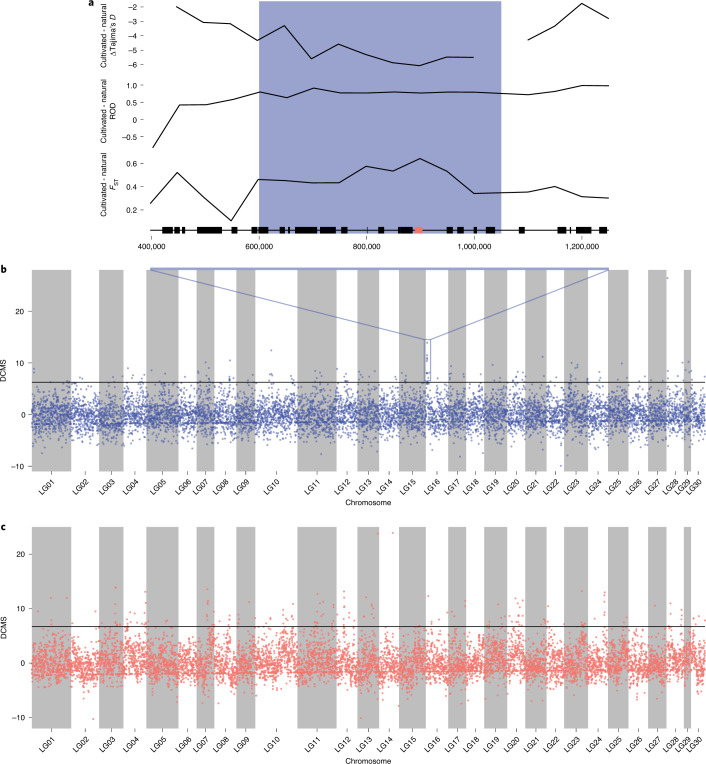
Signatures of selection in *Undaria pinnatifida*. **a**, A close-up of population genetic statistics (*F*_ST_, reduction of diversity (ROD) and Tajima’s *D*) in the linkage group LG16. The blue box highlights the genomic region under putative positive selection, black boxes represent annotated protein-coding genes encoded in this genomic region, and the red box represents a mannitol 1-phosphate dehydrogenase coding gene. **b**, Manhattan plot of the DCMS score calculated in non-overlapping 50-kb windows in the comparison between natural and cultivated populations. **c**, Manhattan plot of the DCMS score calculated in non-overlapping 50-kb windows in the comparison between individuals sampled in Wellington Harbour in 1987 and individuals sampled in Wellington Harbour in 2017.

The distinct genetic structures of *U. pinnatifida* populations (Fig. 2) could be explained by positive selection on many genomic regions. However, the history of these populations (that is, bottlenecks and reduced population effective sizes (that is, neutral evolutionary processes^60^)) could also produce patterns of genetic diversity that resemble selective sweeps. Furthermore, the putative functions of loci implicated in putative selective sweeps can be overinterpreted^61^, and in the absence of other evidence (for example, expression level and phenotype), they should be considered with caution. We identified putative selection signals associated with cultivated or introduced populations of *U. pinnatifida* by using a decorrelated composite of multiple signals (DCMS)^62^ method on three different statistics calculated in 50-kb windows along the genome (Fig. 4 and Supplementary Note).

A comparison of natural and cultivated individuals from the native range revealed that the 508 genes encoded in the 224 genomic windows identified to be under selection (DCMS score *P* value < 0.025) were enriched in several biological processes such as glycolipid biosynthesis and cytokinetic process (Supplementary Note and Supplementary Tables 11 and 12). Intentional selection for increased yield in *U. pinnatifida* culture could explain this enrichment in genes related to carbohydrate biosynthesis. These genes have functions in the alginate, mannitol and sulfate fucan pathways (Supplementary Table 11). However, in total, these pathways contain >150 genes, greatly exceeding the number of genes under putative selection (8 in total). Genomic analysis of *Saccharina japonica* also identified genes involved in carbohydrate metabolism (for example, fructose-1,6-bisphosphate aldolase) in genomic regions under putative selection^63^, but interestingly, different pathways were recovered in the two species. More generally, the biological process selected during cultivation in the two species differed greatly (Supplementary Note). This could indicate that the traits of interest selected by the farmers differ between the two species or that the cultivation processes are at different levels of completion.

**Extended Data Fig. 4:**
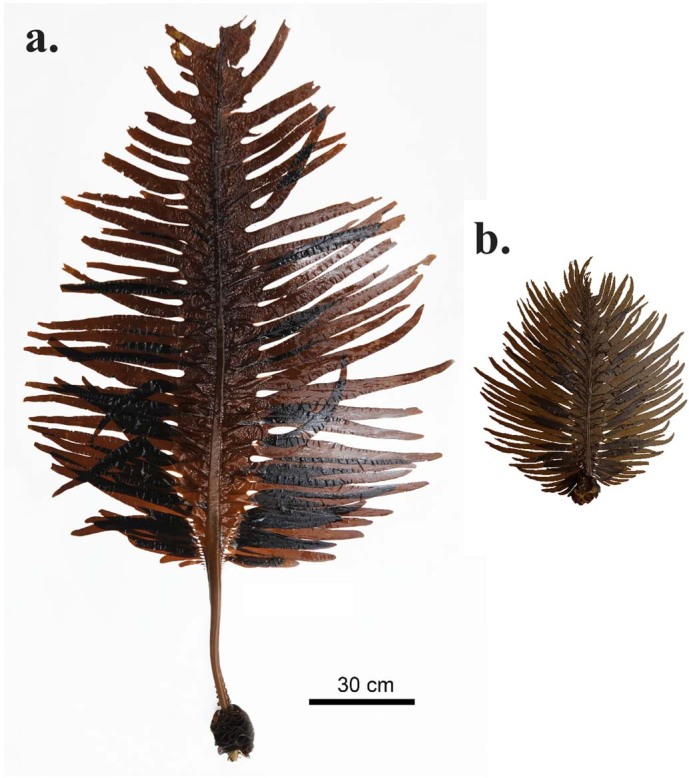
Morphotypes of *Undaria pinnatifida* in Korea. Observed phenotypes of *Undaria pinnatifida* individuals collected in (**a**) a farm in Wando (Korea) and in (**b**) a natural population in Tongyeong (Korea).

The important morphological differences between natural and cultivated individuals suggest that developmental processes have diverged in these two groups (Extended Data Fig. 4). However, gene families that might play a role in development, such as the Cupin-like, C2H2 zinc-finger or imm upregulated genes, were absent from the regions under putative selection despite the large number of copies encoded in the genome of *U. pinnatifida* (19, 34 and 10, respectively). Similarly, with the exception of one copy, peptidase s8 and s53, which were proposed as blade length and width quantitative trait loci in *S. japonica*^64^, were not detected by the DCMS analysis (Supplementary Table 11). The high phenotypic plasticity observed in brown algae^65,66^, and in *U. pinnatifida* in particular^67^, could help explain this absence and support a polygenic basis for the phenotypic differences, perhaps underpinned by differential regulation and post-transcriptional modification. In this context, the enrichment of a variety of genes having regulatory and kinase activities could be linked to selection of regulatory networks underlying development (Supplementary Table 11). Exploratory transcriptome analysis of genes within regions under positive selection revealed that they could potentially have different expression levels when compared to genes of similar functions encoded elsewhere in the genome (Supplementary Note, Supplementary Fig. 11 and Supplementary Table 12). However, these are preliminary results (Supplementary Note) and a more comprehensive transcriptomic analysis is needed to better understand the effect of positive selection on gene expression in the cultivated *U. pinnatifida*.

In contrast to the cultivated versus natural population comparisons, the analysis across 30–60 generations (about 1–2 generations per year^55^) in Wellington Harbour between 1987 and 2017 did not reveal the enrichment of a particular biological function (Supplementary Note and Supplementary Tables 13 and 14). The high variance in allelic frequency resulting from the founding effect during the initial introduction and the insufficient time of 30–60 generations for selection to operate on standing genetic variation could indicate that the signals detected by our analysis result mostly from neutral effects. It is also possible that the relatively wide ecological niche of *U. pinnatifida* and the comparable environments in Korea and New Zealand have suppressed divergence. For example, none of the genes involved in defence mechanisms against infection, such as the vanadium-dependent bromoperoxidases and iodoperoxidases^68^ or the LRR-GTPases of the ROCO family^69^, is encoded in the regions under putative selection. However, a number of the genes under selection appeared to have roles in stress, homeostasis and membrane functions, suggesting adaptation to the New Zealand environment (Supplementary Table 14).

## Conclusion

The generation of a high-quality genome assembly combined with resequencing data from 41 individuals provides a detailed picture of the effect of human activity on genome evolution in *U. pinnatifida*. Our results strongly support the introduction scenario proposed for France and New Zealand and reveal how genome architecture is shaped by introduction history. For individuals in the native range, our analysis revealed unexpected effects of cultivation on the genomic landscape and provided insights into how natural selection may impact these individuals. Furthermore, our study offers a foundation on which future analyses of dispersal and adaptation in new environments can be designed.

In the future, targeted sampling and an explicit experimental design are needed to better connect genetic and phenotypic information. In particular, quantitative trait locus mapping in crosses between cultivars from breeding lines and natural individuals could help elucidate the domestication process in *U. pinnatifida*. In the introduced populations, phenotypic comparisons and environmental measurements in native and introduced sites could allow a genome-wide association study to identify the genetic variants underlying regional phenotypic differences. In particular, the connectivity between the cultivated and natural populations should be assessed to test the role of escaped cultivars in the generation of genetic novelty in nature.

## Methods

### Algal material, genome sequencing and annotation

The *U. pinnatifida* individual used for reference genome sequencing was collected from a longline rope in a culture farm in Wando, Korea on 23 January 2015. High-quality DNA was extracted using a modified cetyl trimethylammonium bromide method (Supplementary Note). According to the instructions of the manufacturers, Illumina paired-end sequencing (PE: 101 bp) and long PacBio reads were sequenced and processed for error correction and quality filtration (Supplementary Note). The sequencing reads were assembled and polished, and finally superscaffolding was performed using data from Shan et al.^36^. The final assembly of the genome (Kr2015) was assessed by alignment of the proteins encoded in the genomes of *E. siliculosus* and *S. japonica* and core eukaryotic genes (Supplementary Table 5 and Supplementary Note).

Transposable elements and repeats were masked in the Kr2015 assembly using a combination of RepeatModeler and RepeatMasker, and their insertion time was estimated (Supplementary Note). For the gene prediction, a collection of proteins from seven species was mapped on the masked Kr2015 assembly and eight complementary DNA libraries were generated and sequenced (Supplementary Note). Genes were predicted using a homology-based and transcriptome-based in-house pipeline and the predicted genes were functionally annotated (Supplementary Note).

### Comparative analysis

Orthologous analysis was conducted on the genome data from a selection of 19 taxa representing the diversity of the stramenopiles. The sequences of orthologous single genes found in all species were aligned and used to reconstruct a maximum-likelihood phylogenetic tree that was used as the backbone of a Dollo parsimony analysis (Supplementary Note). Syntenic analyses were conducted with the Kr2015 gene model against the gene model of the Chinese assembly of *U. pinnatifida* and the gene model of *E. siliculosus* (Supplementary Note)

### Additional *U. pinnatifida* genomes and variant calling

Genomic DNA for a total of 41 *U. pinnatifida* individuals sampled in Korea (natural and cultivated populations), France (introduced populations) and New Zealand (introduced populations) was extracted, and for each individual, ~30× coverage of short-read data were generated (Supplementary Table 8, Supplementary Note). For each individual, after trimming of sequencing adapters and low-quality bases, the reads were mapped in the Kr2015 genome. These mapping data were used to call variants for each individual before all variants were combined to form the primary variant dataset containing 25,414,685 variants (21,619,805 SNPs and 3,794,880 indels). The variants were filtered for quality thresholds, allele frequency and genotyping rate to produce a final dataset of 7,253,541 (6,123,124 SNPs and 1,130,417 indels) variants.

### Population genomics

The population structure was investigated with PCA, phylogenetic tree reconstruction and admixture analyses (Supplementary Note). For each type of population, the expected heterozygosity (*H*_e_), *π*, fixation index (*F*_IS_), LD (estimated from *r*^2^) and ROH (that is, chromosome fragments within a single individual that have shared parental ancestry) were estimated (Supplementary Note). Detection of regions affected by selection was conducted using a combination of statistics calculated in non-overlapping 50-kb windows: reduction of diversity, delta Tajima’s *D* and population differentiation (*F*_ST_). For each window and each statistic, a *P* value was determined and these were used to calculate the DCMS value of each window (Supplementary Note).

### Reporting Summary

Further information on research design is available in the Nature Research Reporting Summary linked to this article.

## Supplementary information

Supplementary Information Supplementary Figs. 1–17 and Note.

Reporting Summary

Peer review information

Supplementary Tables Supplementary Tables 1–19.

## Data Availability

The raw sequencing reads were deposited in the National Center for Biotechnology Information database under the BioProject accession code PRJNA646283. The assemblies, gene model and functional annotation were deposited in the Marine Genome Information Center (http://www.magic.re.kr/) database under the accession code MA00358.
