# Supplementary material for: A genome-wide investigation of the effect of farming and human-mediated introduction on the ubiquitous seaweed Undaria pinnatifida
Source: Nat Ecol Evol. 2021 Jan 25;5(3):360–8. doi: 10.1038/s41559-020-01378-9 (PMC7929912; doi:10.1038/s41559-020-01378-9)
Supplement: Supplementary file 1 — Supplementary Figs. 1–17 and Note. [file 41559_2020_1378_MOESM1_ESM.pdf]

natureresearch

https://doi.org/10.1038/s41559-020-01378-9

Supplementary information

A genome-wide investigation of the effect of farming and human-mediated introduction on the ubiquitous seaweed *Undaria pinnatifida*

In the format provided by the authors and unedited

Supplementary Material

for

A genome-wide investigation of the effect of farming and human-mediated introduction on the ubiquitous seaweed *Undaria pinnatifida*

Louis Graf[1], Younhee Shin[1], Ji Hyun Yang[1], Ji Won Choi[1], Il Ki Hwang[2], Wendy Nelson[3], Debashish Bhattacharya[4], Frédérique Viard[5,6], Hwan Su Yoon[1,*]

[1]Department of Biological Sciences, Sungkyunkwan University, Suwon 440-746, Korea

[2]Aquaculture Management Division, National Institute of Fisheries Science, Busan, 46083, South Korea

[3]National Institute of Water & Atmospheric Research, University of Auckland, New Zealand

[4]Department of Biochemistry and Microbiology, Rutgers University, New Brunswick, NJ 08901, USA

[5]Sorbonne Université, CNRS, AD2M, Station Biologique de Roscoff, France

[6]ISEM, Univ Montpellier, CNRS, EPHE, IRD, Montpellier, France

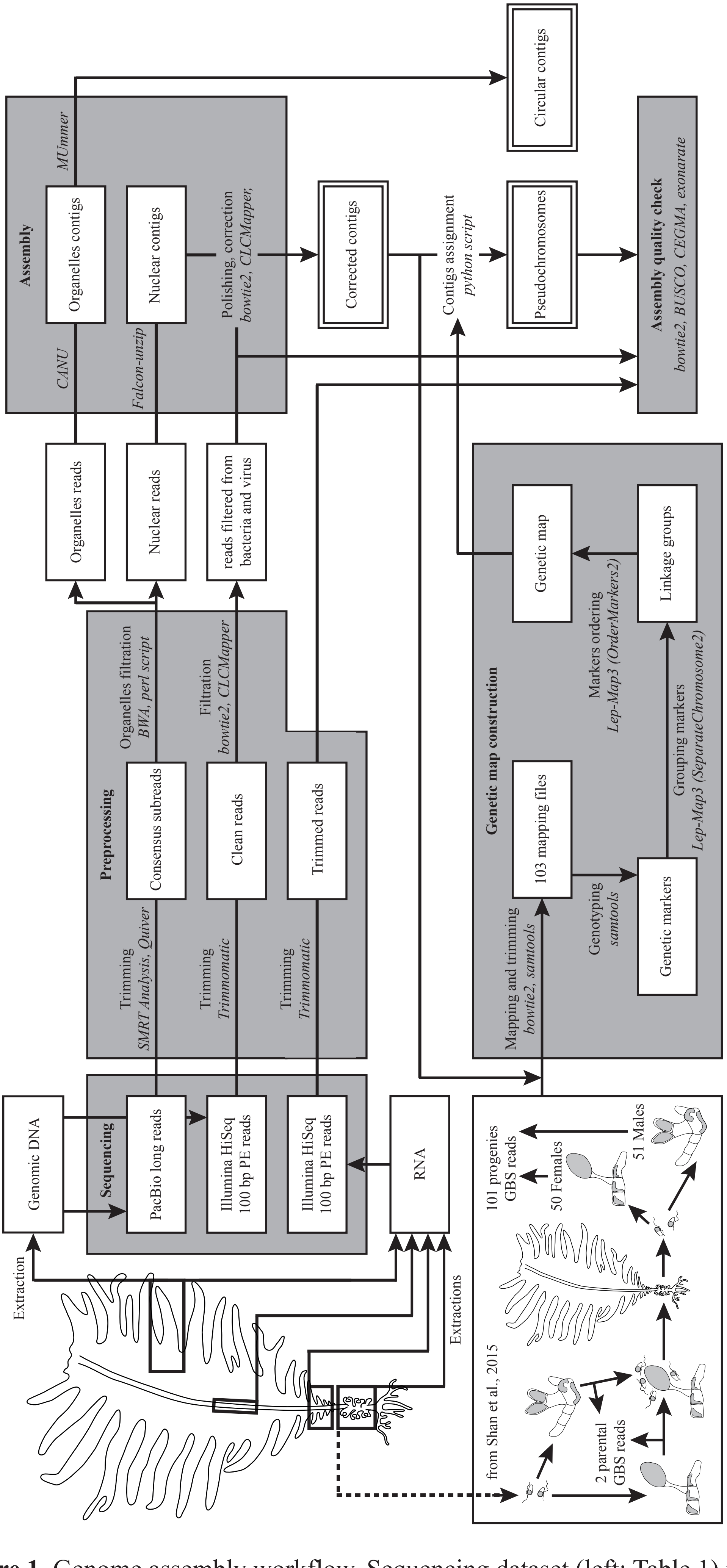

Supplementary Figure 1. Genome assembly workflow. Sequencing dataset (left; Table 1) was processed through the workflow using the tools indicated in Italics. PE stands for paired-end reads. GBS stands for genotyping-by-sequencing.

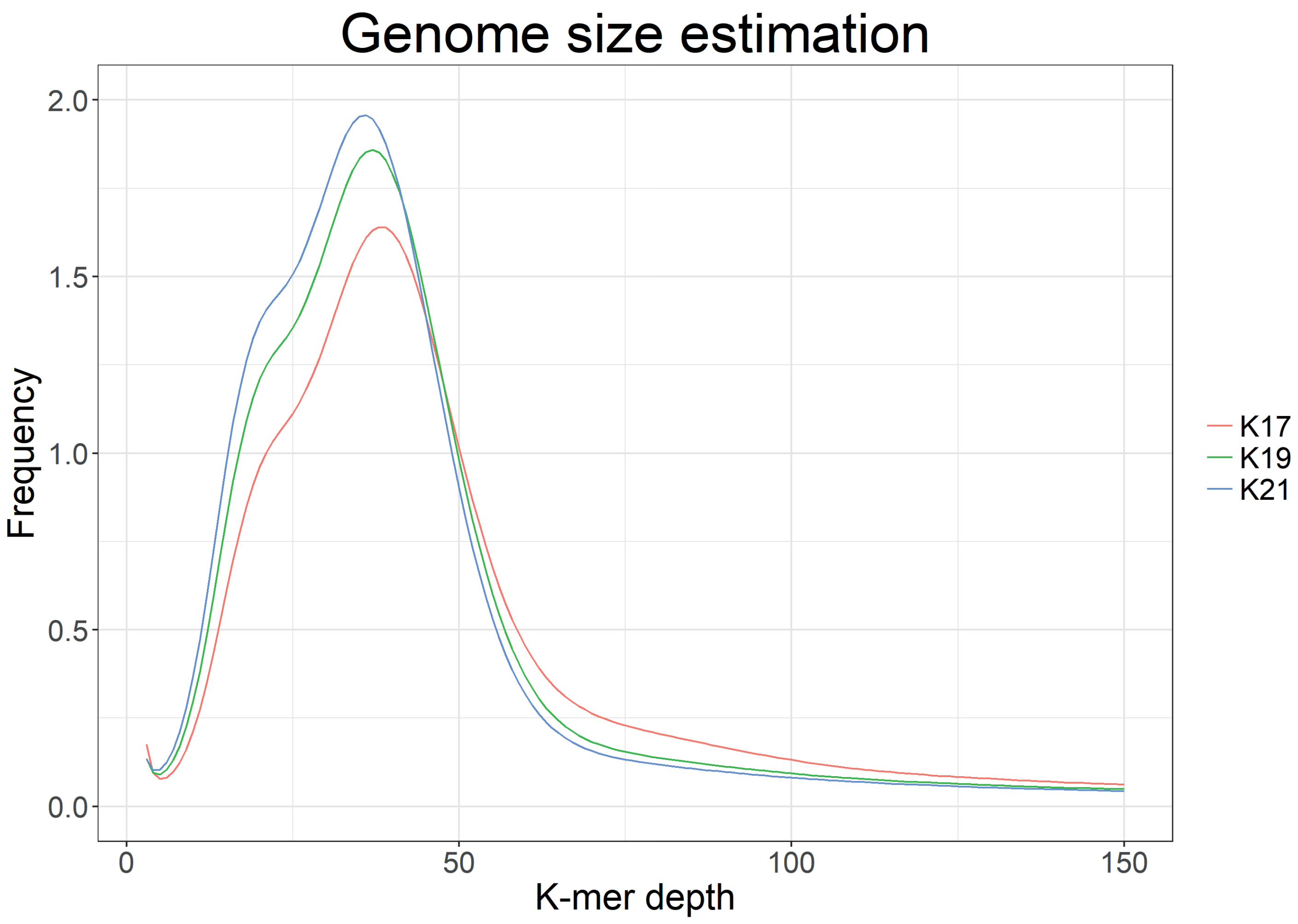

K-mer	Depth	Estimated genome size
17	45.4	556,299,307
19	45.3	557,392,637
21	45.2	558,546,707

Supplementary Figure 2. Genome size estimation for the nuclear genome of *Undaria pinnatifida* calculated from k-mer frequency distributions based on the filtered nuclear Illumina paired-end reads.

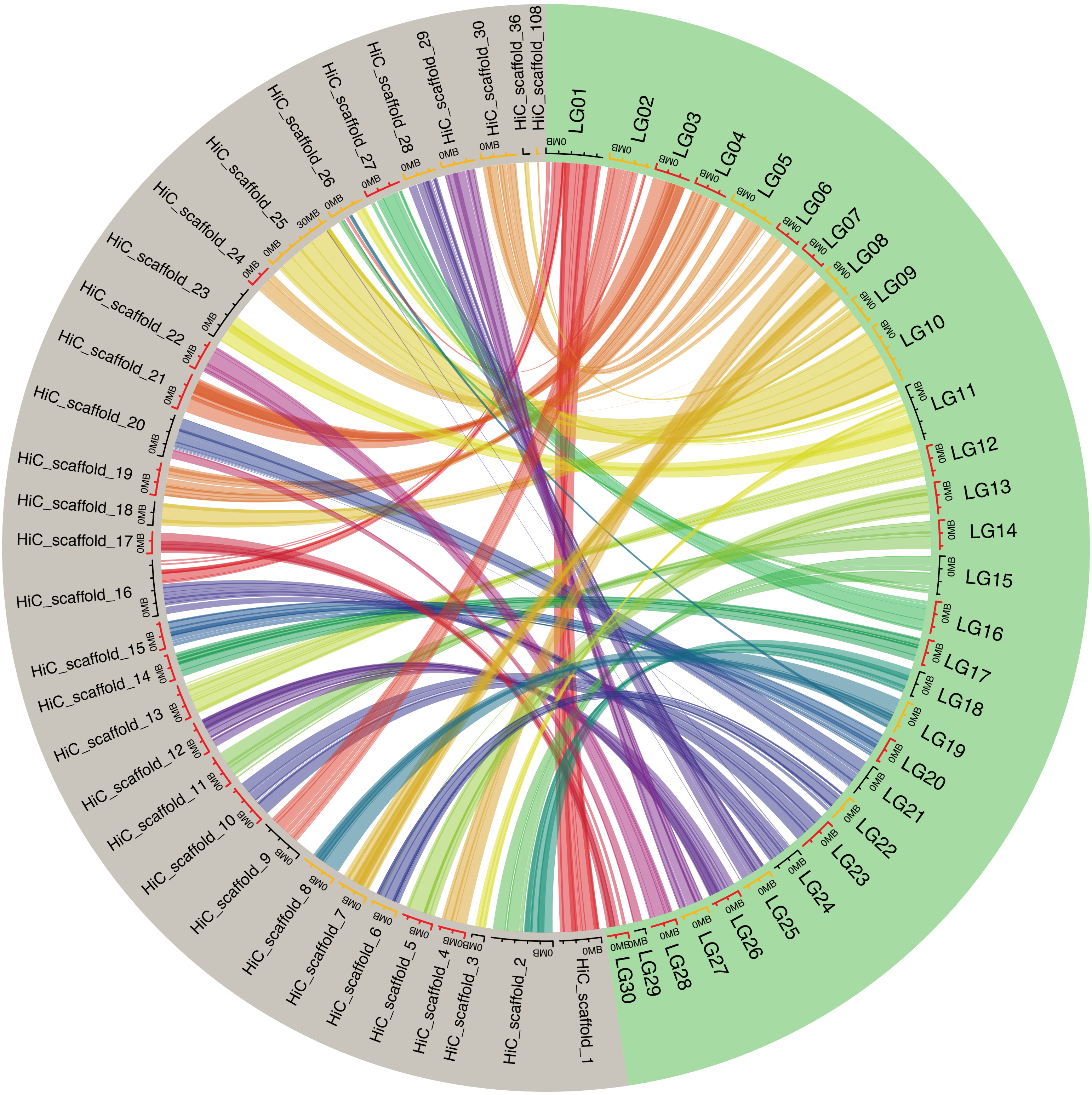

Supplementary Figure 3. Syntenic alignment of the Kr2015 genome and the Chinese genome assembly of *Undaria pinnatifida*. Pseudochromosomes marked in red indicate exclusively shared pseudochromosomes. Pseudochromosomes marked in orange indicate almost exclusively shared pseudochromosomes.

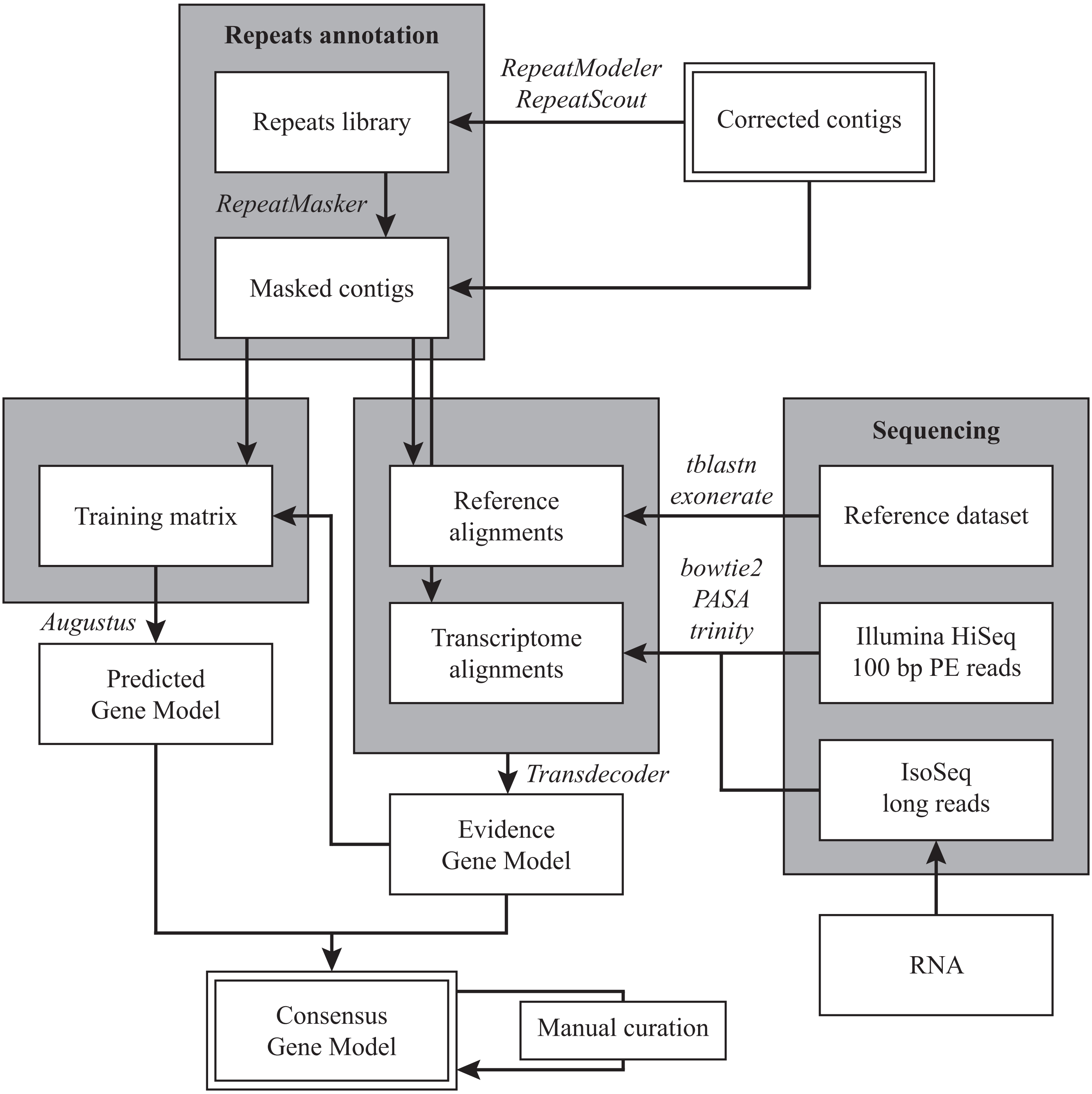

Supplementary Figure 4. Gene annotation workflow. The reference dataset (right) was composed of proteins recovered from the annotation of various brown algal genomes (*Ectocarpus siliculosus*, *Cladosiphon okamuranus* and *Saccharina japonica*). Stramenopile genomes (*Nannochloropsis gaditana*, *Phaeodactylum tricornutum*, *Thalassiosira pseudonana*) and the algal proteins from the uniprot and plant transcription factor databases. RNA sequencing data and reference data were processed through the workflow using the tools indicated in italics. PE stands for paired-end reads.

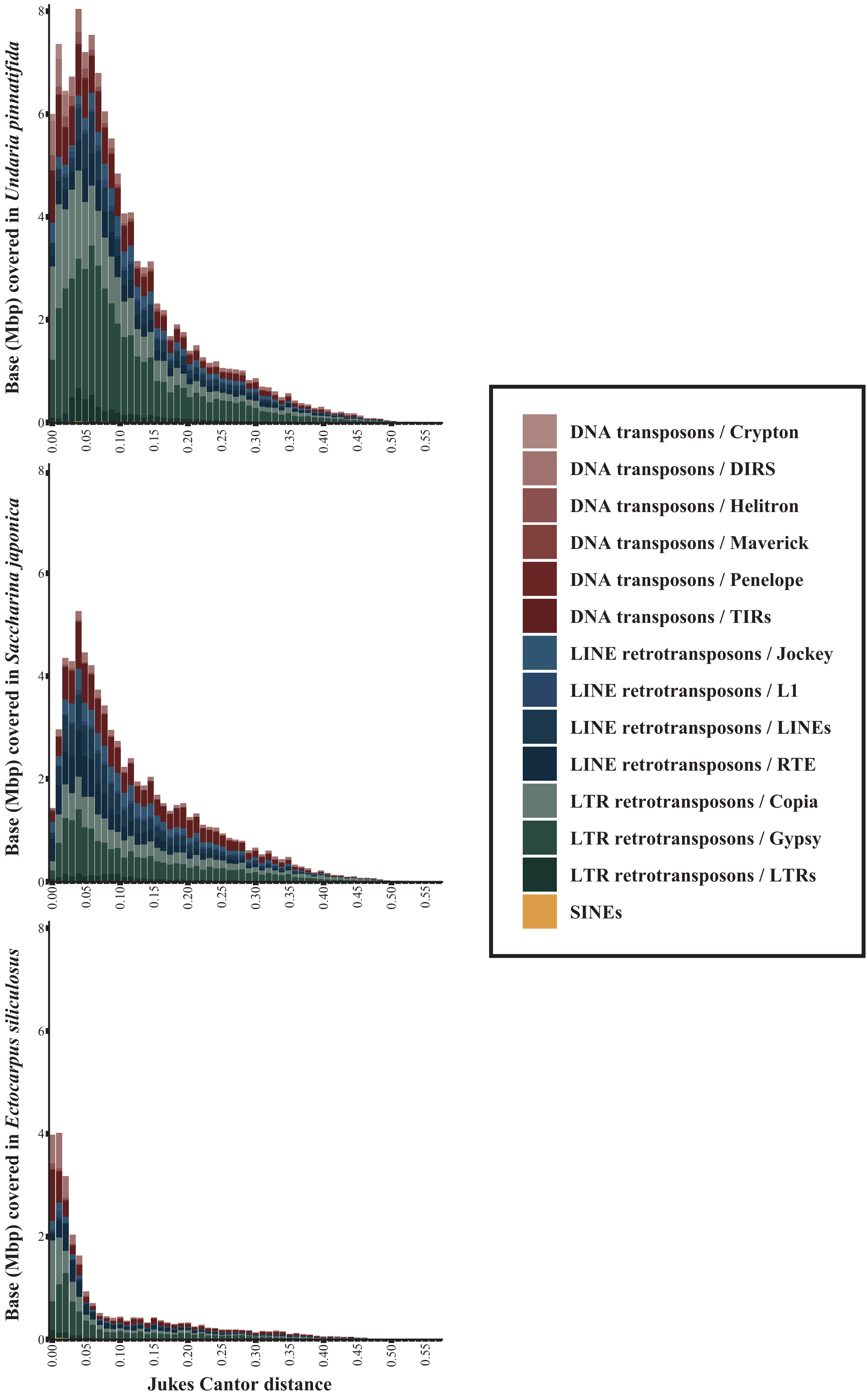

Supplementary Figure 5. Timing of repetitive element insertion and abundance in the genomes of *Undaria pinnatifida*, *Saccharina japonica* and *Ectocarpus siliculosus*. The x-axis is the distance of individual repeats to the consensus repeat (estimated with the Jukes-Cantor model) and the y-axis is the percentage of genome covered. Repeat elements that were not annotated were excluded from this analysis.

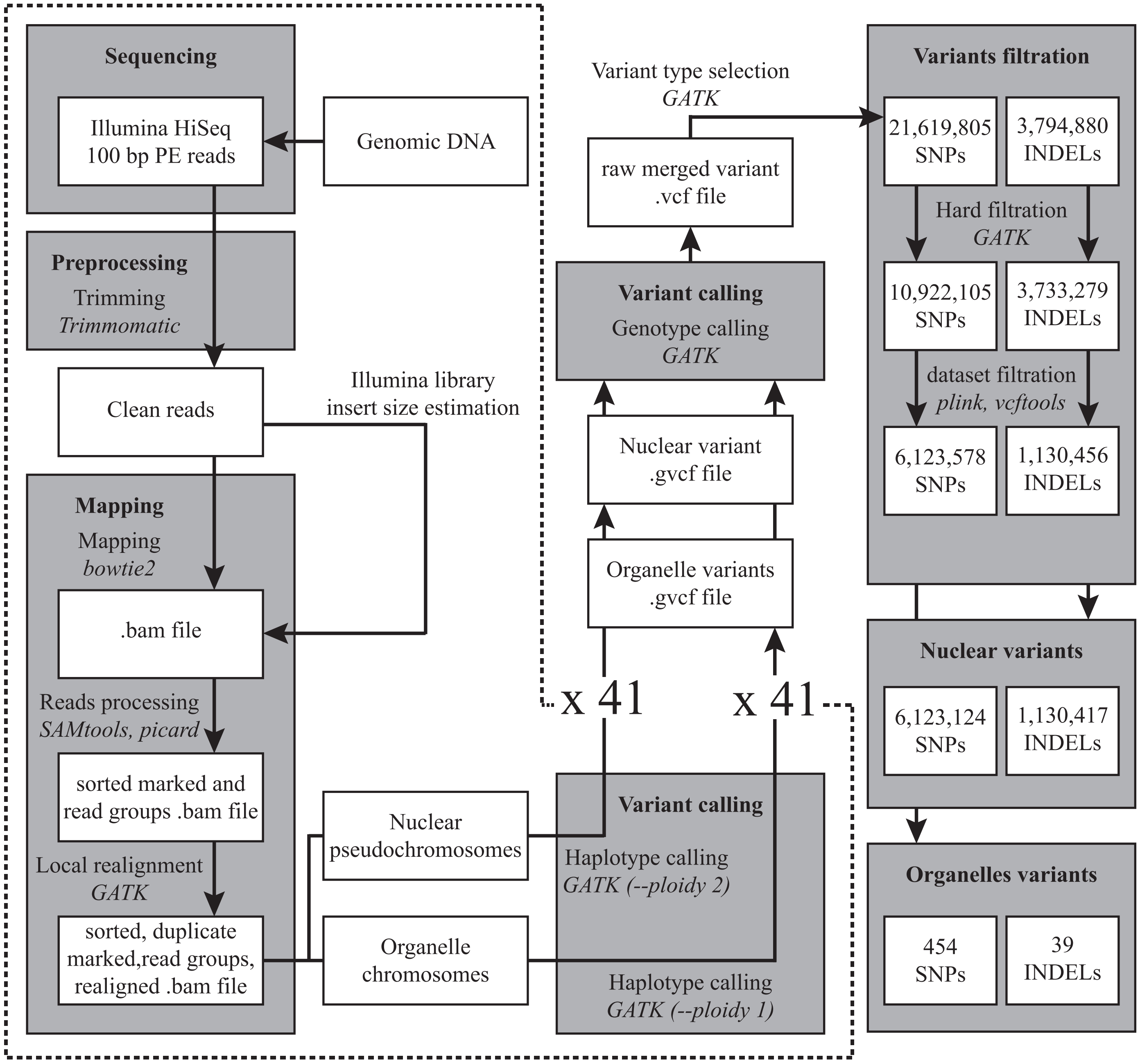

Supplementary Figure 6. Variant calling workflow. For each individual genomic DNA was extracted, sequenced and processed through the work-flow using the tools indicated in italic. PE stands for paired-end reads.GBS stands for genotyping-by-sequencing.

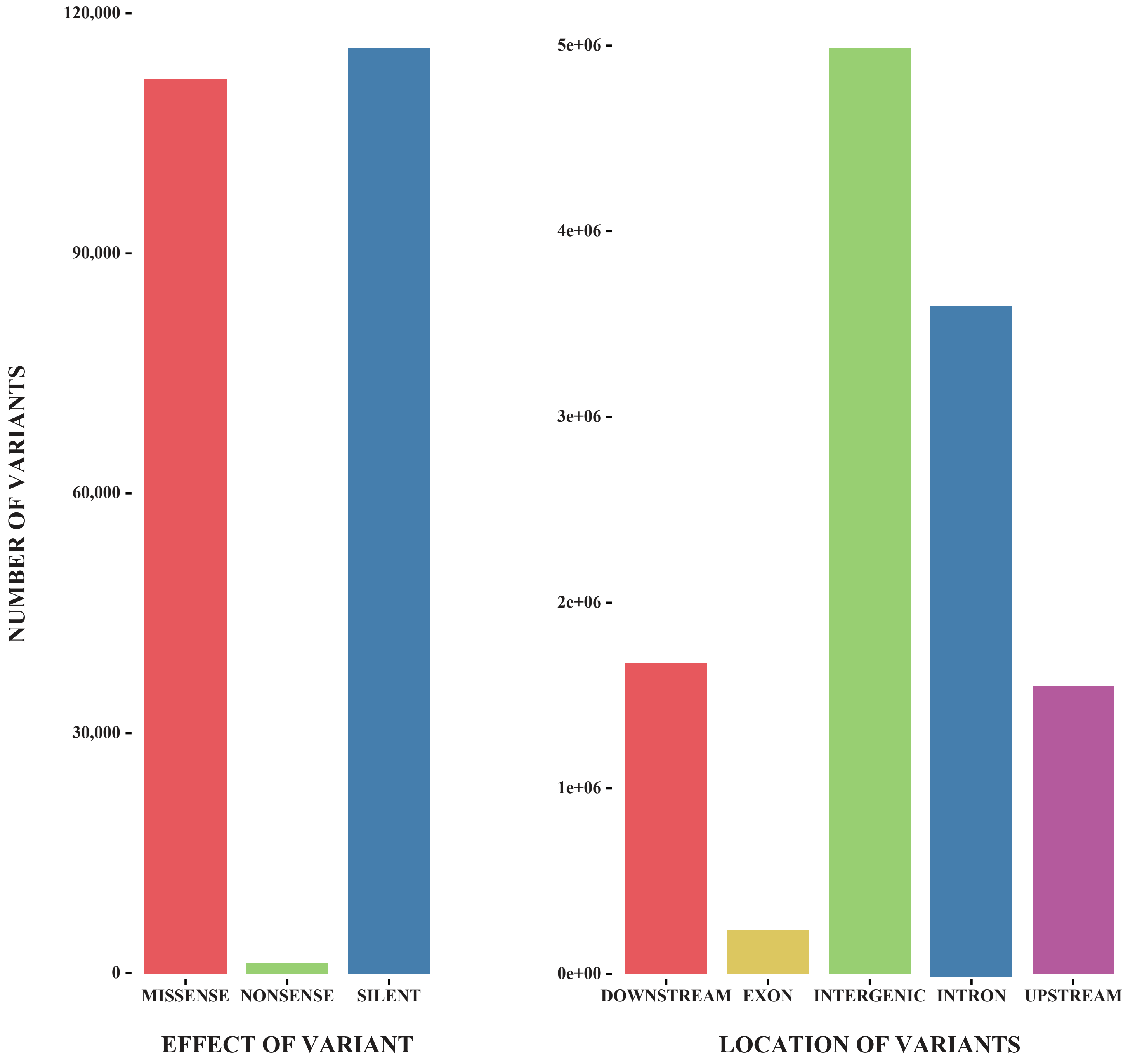

Supplementary Figure 7. Annotation summary of the variants called in the 41 individuals of *Undaria pinnatifida*. The left barplot shows the effect of variants in coding sequence: missense (non-synonymous) variants, nonsense variants that encode a stop codon, silent (synonymous) variants. Location of variant: downstream and upstream are variants located 5 kbp in the 5′ or 3′ regions of genes.

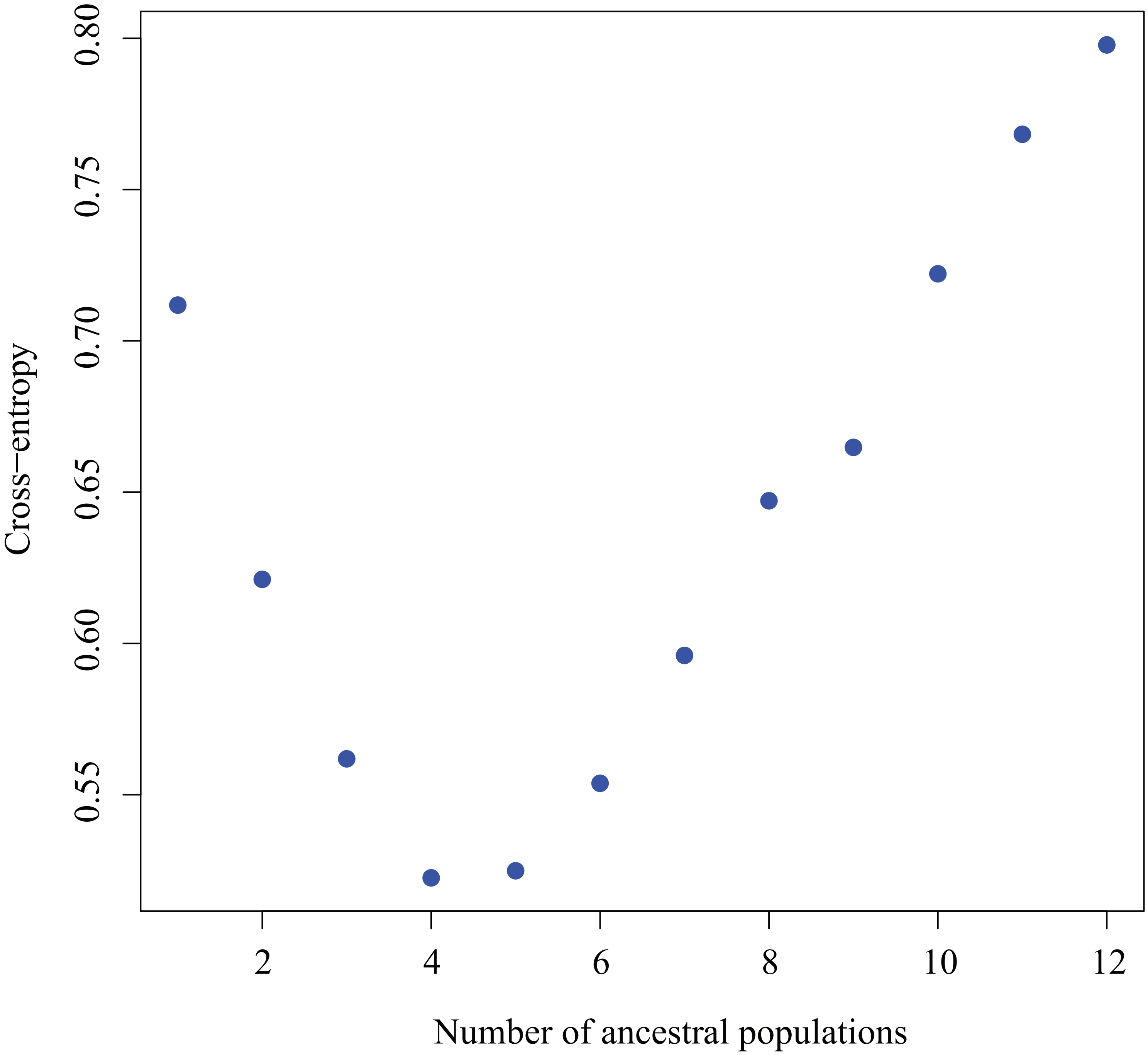

Supplementary Figure 8. Value of the cross-entropy criterion as a function of the number of populations estimated by the *snmf* algorithm.

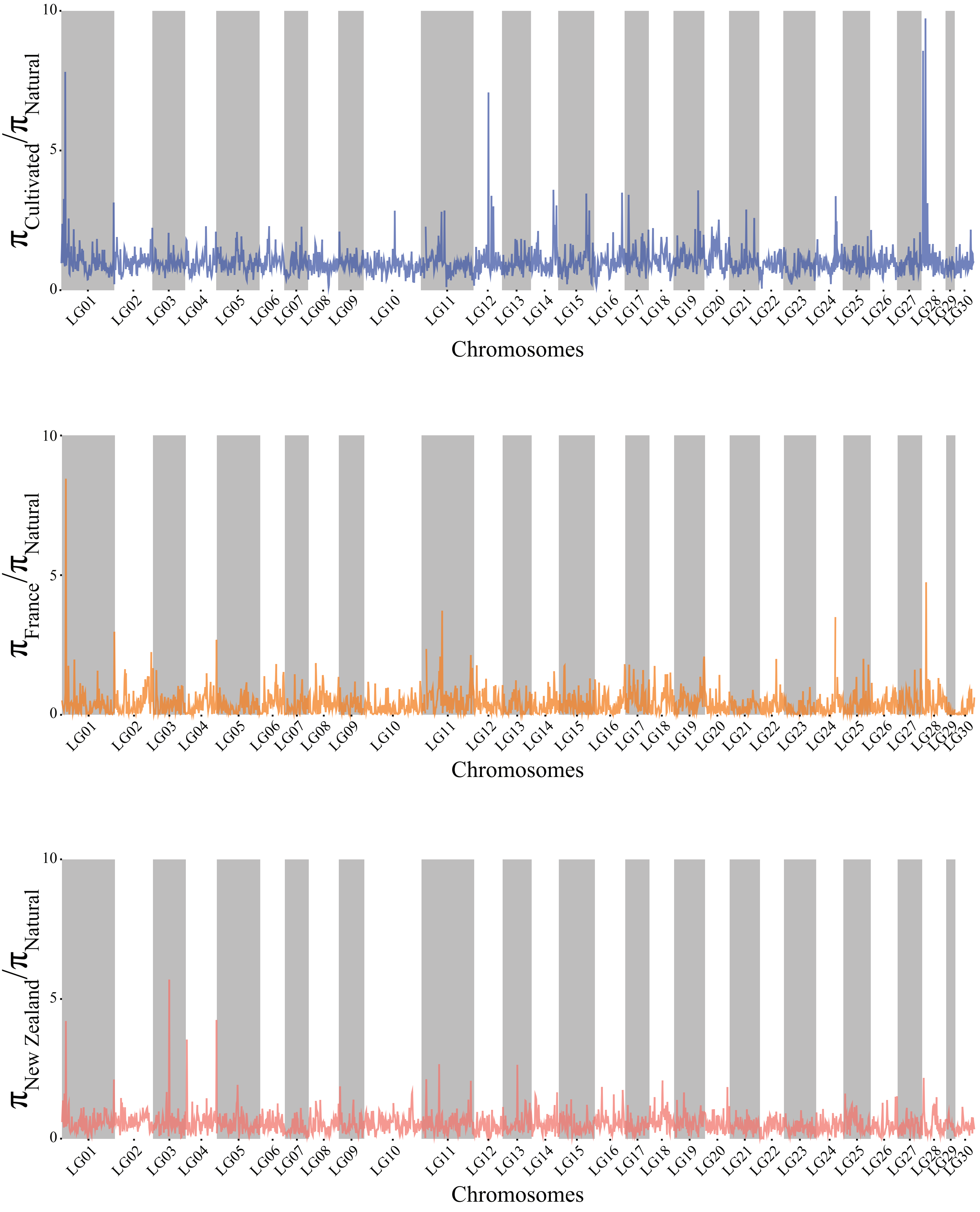

Supplementary Figure 9. Graph lines of the geneteic diversity (π) ratio calculated in non-overlapping 250 kb windows between the cultivated and natural populations (blue), the French and natural populations (yellow) and the New Zealand and natural populations (red).

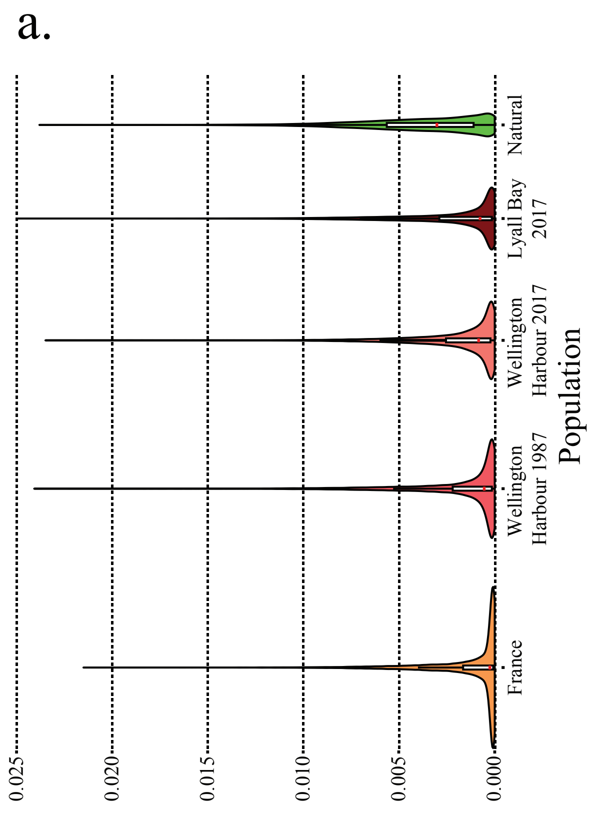

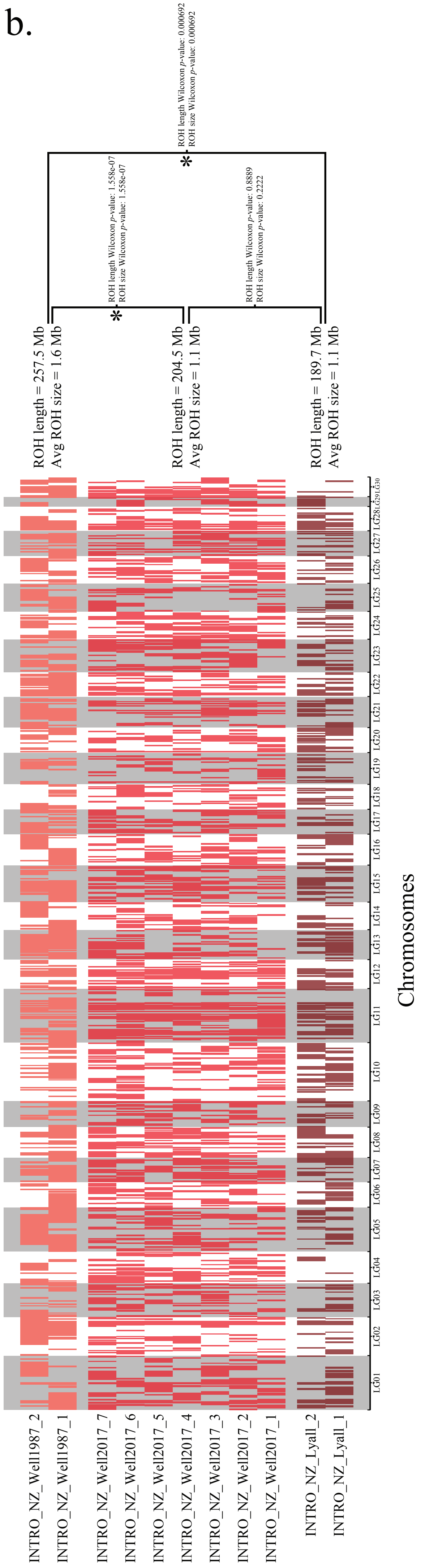

Supplementary Figure 10. (a) Violin plot of the genetic diversity estimated by π in non-overlapping 10 kb windows. (b) Run of homozygosity (ROH) in the 11 individuals from New Zealand.

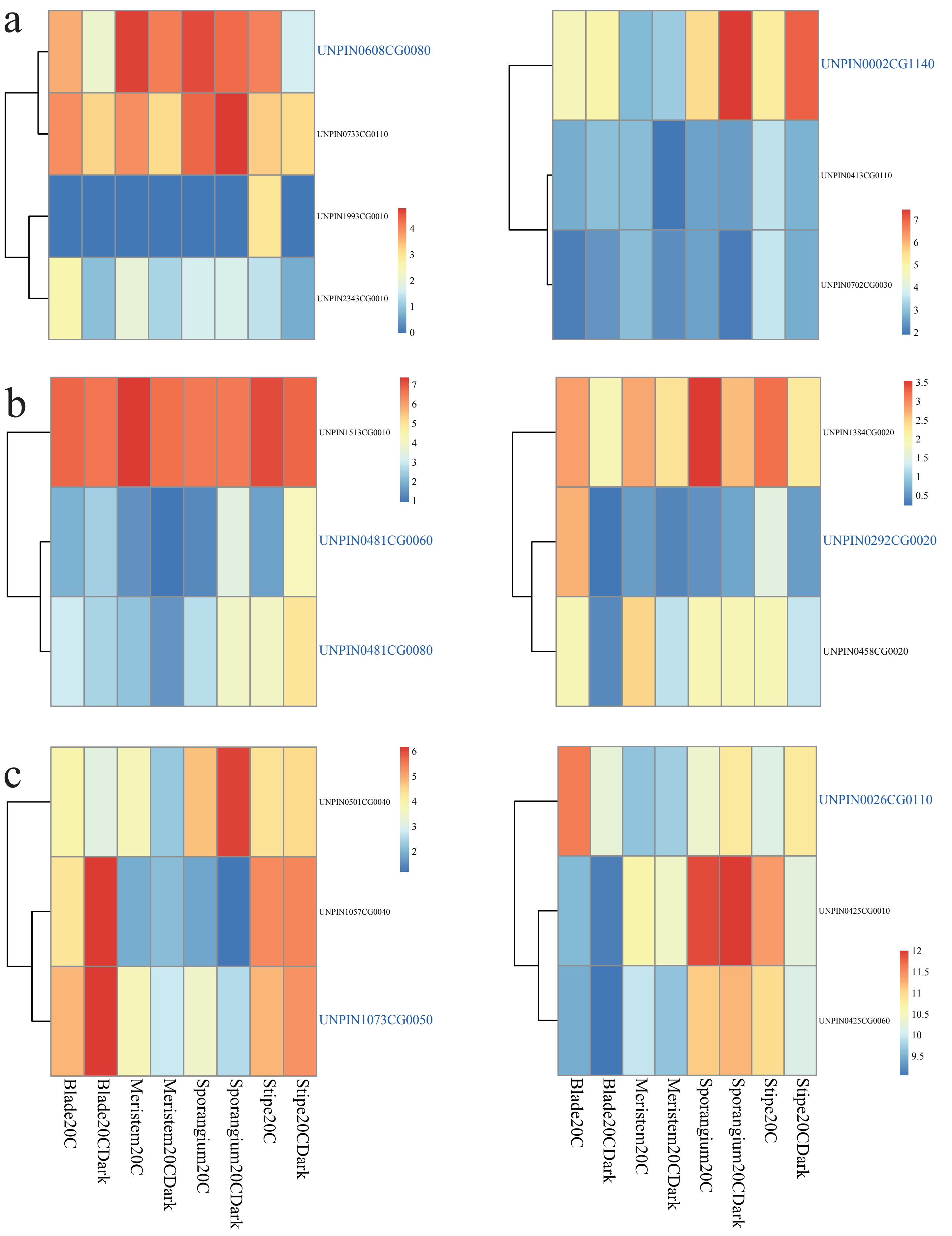

Supplementary Figure 11. Expression patterns of ortholog genes encoded in *Undaria pinnatifda*. Heatmap of the log2 transcripts per million (TPM) in four tissues submitted to two different treatments (Supplementary Note). Genes in blue are encoded in regions under putative positive selection. (a) Ortholog groups in which the member encoded in a genomic region under putative positive selection showed increased expression (Wilcoxon rank sum test p-value < 0.05). (b) Ortholog groups in which the member encoded in a genomic region under putative positive selection showed decreased expression (Wilcoxon rank sum test p-value < 0.05). (c) Ortholog groups in which the member encoded in a genomic region under putative positive selection showed no difference in expression (Wilcoxon rank sum test p-value > 0.05).

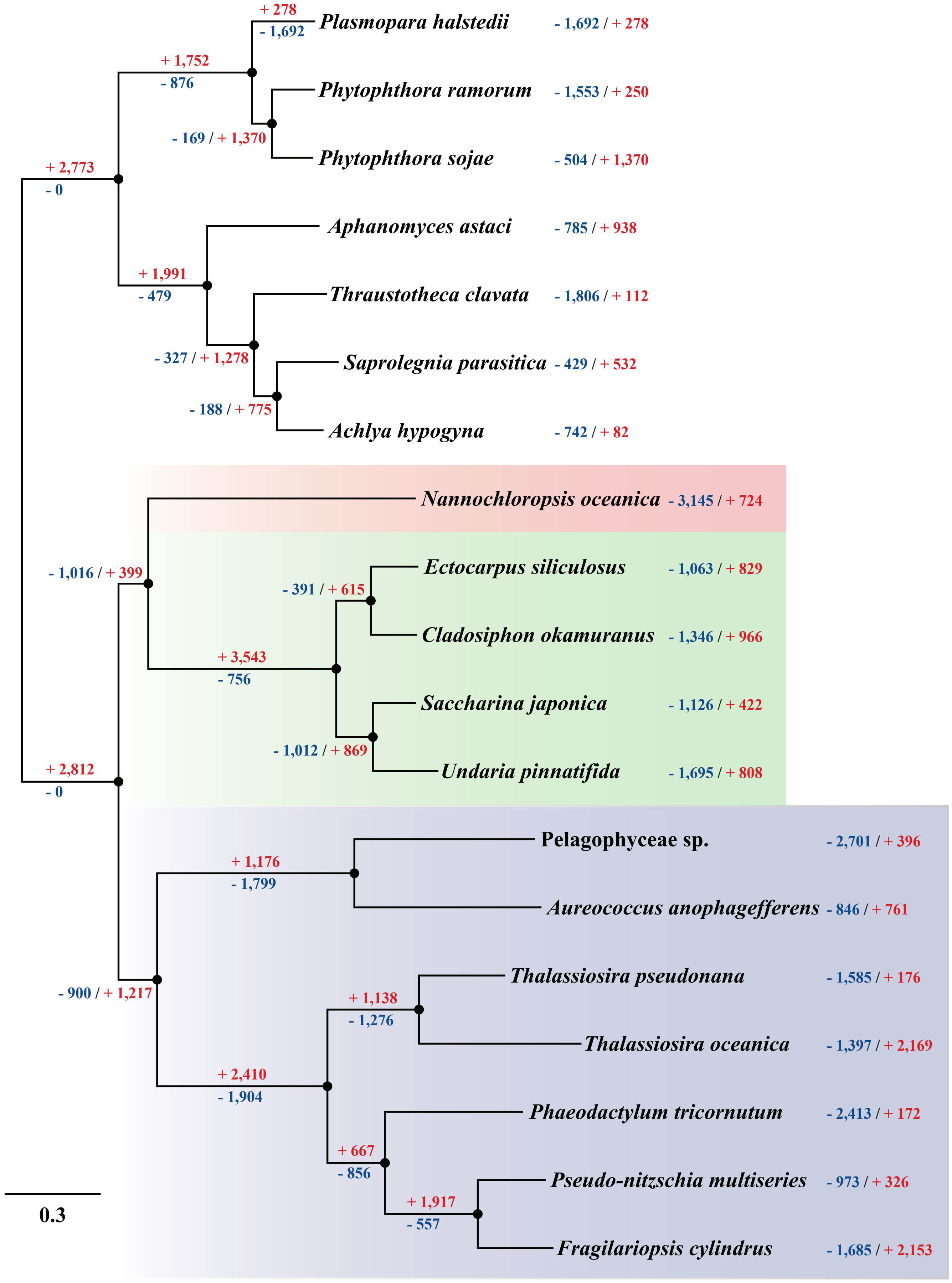

Supplementary Figure 12. Maximum likelihood phylogenetic tree of the Stramenopiles reconstructed with the alignment of 459 orthologous genes shared by all 20 taxa. The tree was inferred using IQTree with an independent model of evolution for every gene. Black circles on a node represent 100% support estimated by IQTree with the UltraFast Bootstrap Approximation algorithm. Scale bar = 0.3 substitutions per site. Ortholog families gained and lost, evaluated by Dollop, are shown near the branch and for every taxa. Gained orthologous gene families are shown in red. Lost orthologous gene families are shown in blue. The three recognized photosynthetic clades of the Stramenopiles, SI (green background), SII (red background) and SIII (blue background) were recovered.

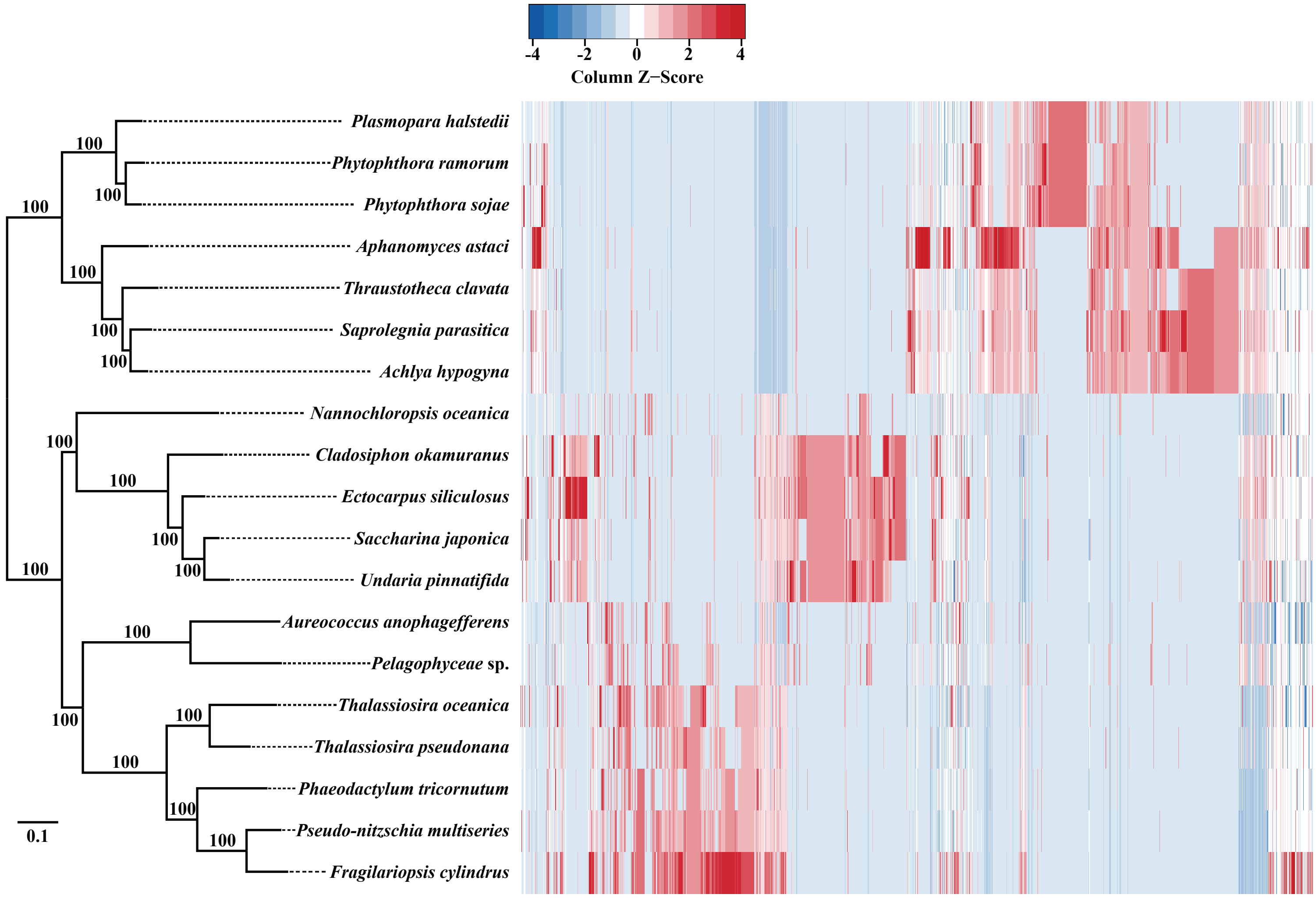

Supplementary Figure 13. K-means clustering of orthologous gene families based on gene abundance in each species. Each column represents a gene family and each row, a species. The species were ordered according to the tree reconstructed in Supplementary Figure 12.

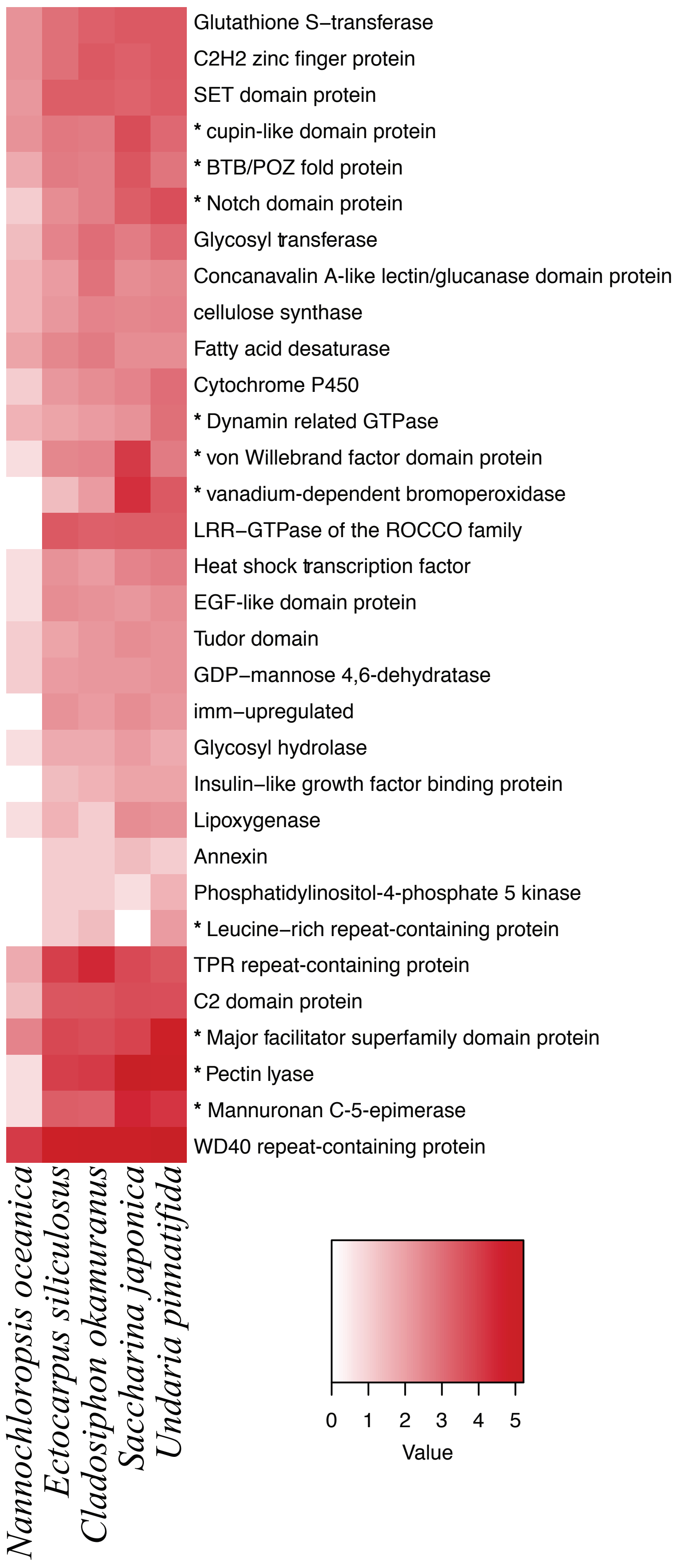

Supplementary Figure 14. Comparison of the number of genes in key gene families related to the evolution of the brown algae. Each row represents a gene family. Each column represents one of the four brown algae species (i.e. *Cladosiphon okamuranus*, *Ectocarpus siliculosus*, *Saccharina japonica* and *Undaria pinnatifida*) or the Eustigmatophyceae species, *Nannochloropsis oceanica*. "*" indicates gene families that were significantly (chi square test p-value < 0.05) enriched in Laminariales compared with Ectocarpales.

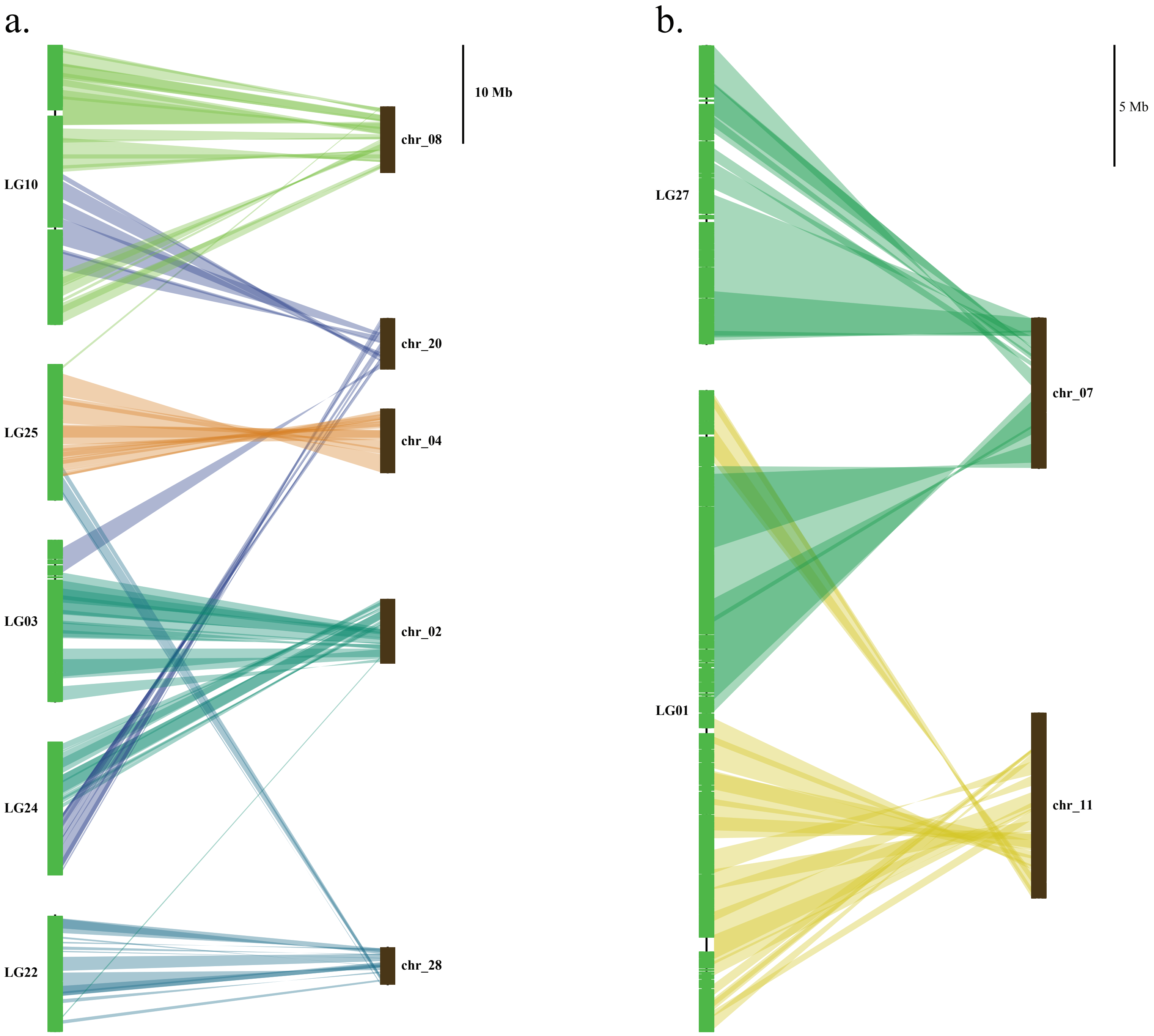

Supplementary Figure 15. (a) Syntenic alignment between pseudochromosomes LG03, LG10, LG22, LG24, LG251 of Kr2015 and chromosome chr_02, chr_04, chr_08, chr_20, chr_28 of *Ectocarpus siliculosus*. (b) Syntenic alignment between pseudochromosomes LG27, LG01 of Kr2015 and chromosome chr_07, chr_11 of *Ectocarpus siliculosus*.

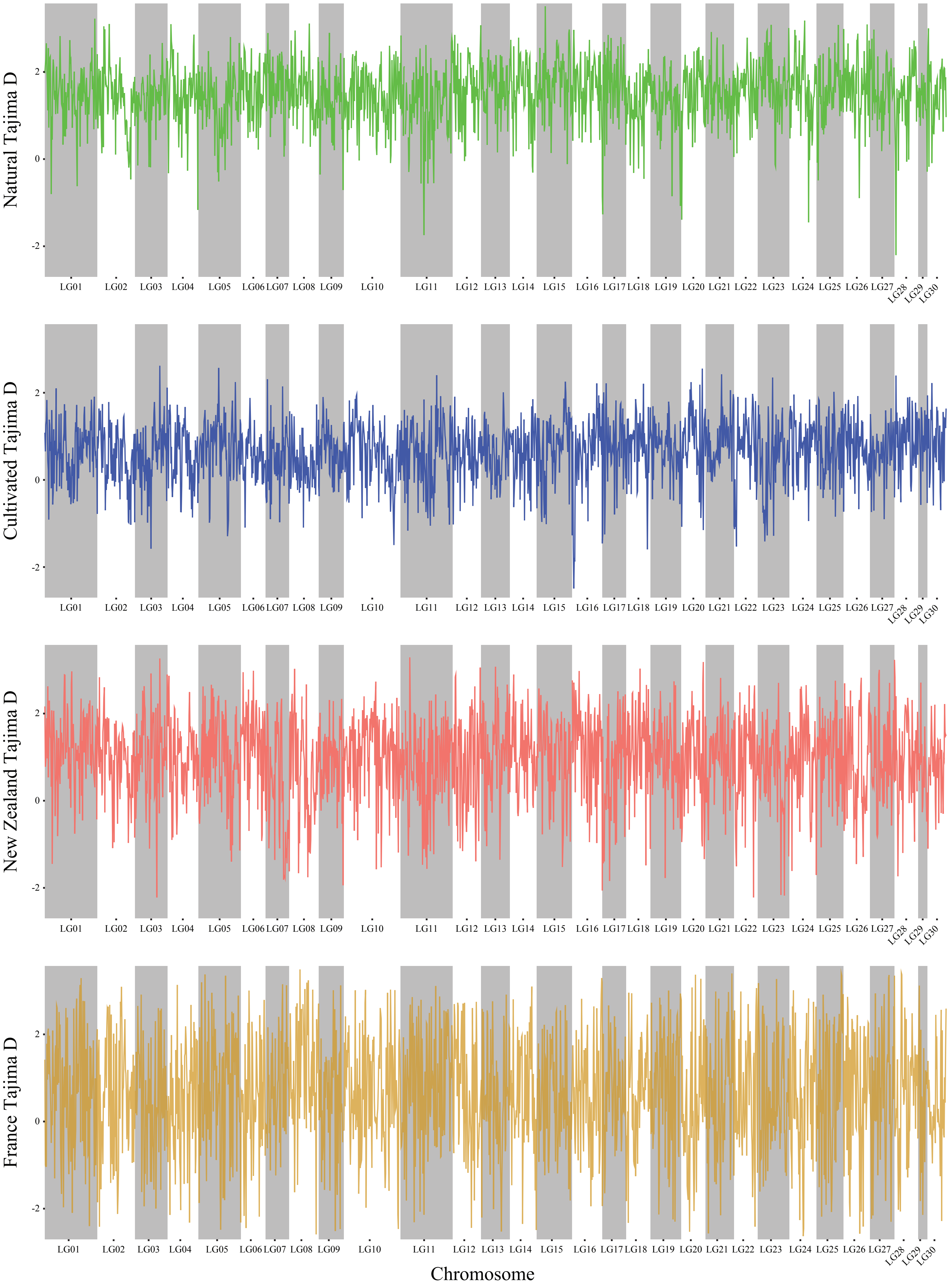

Supplementary Figure 16. Graph lines of the Tajima's D calculated in non-overlapping 50 kb windows in the natural populations (green), the cultivated populations (blue), the New Zealand populations (red) and the French populations (yellow).

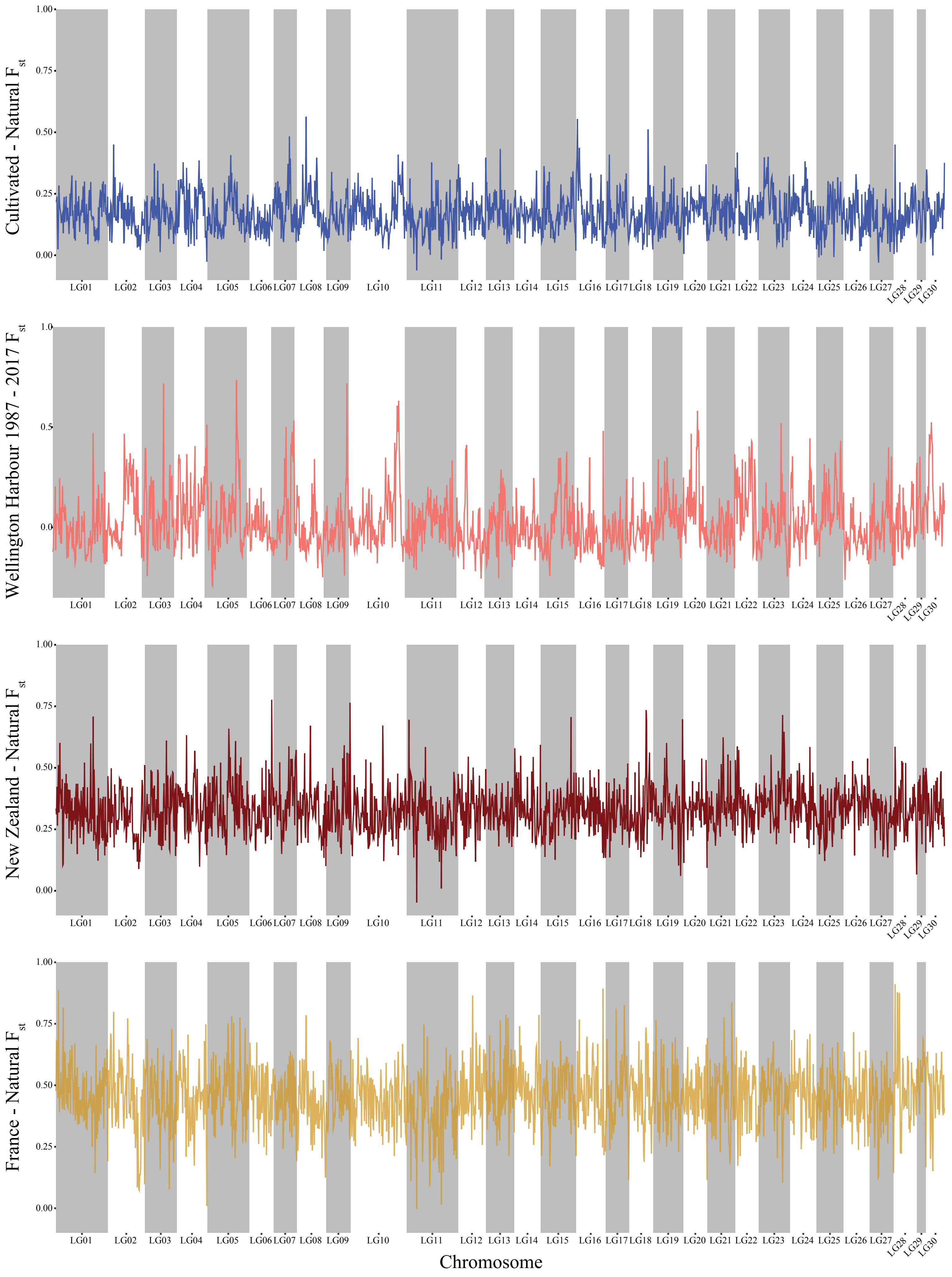

Supplementary Figure 17. Graph lines of the population differentiation (F_{ST}) calculated in non-overlapping 50 kb windows between the cultivated and natural populations (blue), the Wellington 1987 individuals and Wellington 2017 individuals (light red), the New Zealand and natural populations (dark red) and the French and natural populations (yellow).

Supplementary Note

1 - Sequencing, assembly, annotation of the Kr2015 genome. 2
1 - 1 Alga material and DNA isolation 2
1 - 2 Genomic Sequencing 2
1 - 2 - 1 PacBio library and sequencing 2
1 - 2 - 2 Illumina paired-end library and sequencing 3
1 - 3 RNA sequencing 4
1 - 3 - 1 Algal material and RNA isolation 4
1 - 3 - 2 Isoseq library cDNA and sequencing 4
1 - 3 - 3 Illumina paired-end cDNA library and sequencing 5
1 - 3 Genome assembly 6
1 - 4 Superscaffolding 7
1 - 5 Assembly quality evaluation 9
1 - 6 Organellar genomes 10
1 - 7 Genome annotation 11
1 - 7 - 1 Transposable elements and repetitive elements 11
1 - 7 - 2 Gene prediction 11
2 - Comparative genomics 12
2 – 1 Role of the transposable elements in the genome size determination 12
2 - 2 Genome organisation of the brown algae 14
2 - 3 Orthologous analysis and Dollo parsimony analysis 15
2 - 3 Synteny analysis 18
3 - Population genomics 20
3 - 1 Algal material 20
3 - 2 DNA isolation and Illumina paired-end sequencing 21
3 - 3 Read mapping and variant calling 22
3 - 4 Genome polymorphism across individuals 23
3 - 4 - 1 Principal Component Analysis 23
3 - 4 - 2 Phylogenetic tree 23
3 - 4 - 3 Admixture analysis 23
3 - 5 Genome landscape 24
3 - 5 - 1 Genetic diversity estimators 24
3 - 5 - 2 Linkage disequilibrium 24
3 - 5 - 3 Run of homozygosity 25
3 - 6 Selection 26
References 29

1 - Sequencing, assembly, annotation of the Kr2015 genome.

1 - 1 Alga material and DNA isolation

Blade tissue of the sporophyte was flash frozen in liquid nitrogen and ground to powder using the Automill TK-AM5 frozen crusher (http://www.tokken.jp). 400 µL of a modified lysis buffer was added to 500 mg of the ground sample. The modified lysis buffer consisted of 2M NaCl, 50mM Na2EDTA (pH 8.0), 2% polyvinylpyrolidone (PVP)-40, 0.1% BSA, 0.4M sucrose, and 50mM CaCl2. Homogenates were then incubated for 30 min at 37ºC. After lysis, samples were centrifuged at 15,700g for 15 min at room temperature. The supernatant was transferred into a new tube. After the lysis step, total genomic DNA was extracted using the DNeasy Plant Mini kit (Qiagen), following manufacturer's instructions. After the DNA extraction step, a purification step was performed according to the "Guidelines for Using a Salt: Chloroform Wash to Clean Up gDNA" (Pacbio samplenet, Shared Protocol).

1 - 2 Genomic Sequencing

1 - 2 - 1 PacBio library and sequencing

Using the covaris G-tube, 20 Kb fragments were generated by shearing genomic DNA according to the manufacturer's recommended protocol. The AMpureXP bead purification system was used to remove the small fragments. A total of 5 µg for each sample was used as input into library preparation. The SMRTbell library was constructed using SMRTbell™ Template Prep Kit 1.0 (PN 100-259-100). Using the BluePippin size selection system the small fragments were removed to obtain a large-insert library.

After sequencing primers were annealed to the SMRTbell template, DNA polymerase was bound to the complex (DNA/Polymerase Binding kit P6). Following the polymerase binding reaction, the MagBead Kit was used to bind the library complex with MagBeads before sequencing. MagBead bound complexes enabled more reads per SMRT Cell. This polymerase-SMRTbell-adaptor complex was then loaded into zero-mode waveguides (ZMWs). The SMRTbell library was sequenced using 92 cells SMRT cells (Pacific Biosciences) using C4 chemistry (DNA sequencing Reagent 4.0) and 1 x 240 minute movies were captured for each SMRT cell using the PacBio RS (Pacific Biosciences) sequencing platform (Supplementary Table 1).

1 - 2 - 2 Illumina paired-end library and sequencing

DNA library was prepared according to Illumina Truseq Nano DNA Library prep protocol. For sample library preparation, 0.2 μg for insert 550 bp of high molecular weight genomic DNA were randomly sheared to yield DNA fragments using the Covaris S2 system. The fragments were blunt ended and phosphorylated, and a single 'A' nucleotide was added to the 3' ends of the fragments in preparation for ligation to an adapter that has a single-base 'T' overhang. Adapter ligation at both ends of the genomic DNA fragment conferred different sequences at the 5' and 3' ends of each strand in the genomic fragment. Ligated DNA was PCR amplified to enrich for fragments that have adapters on both ends. The quality of the amplified libraries was verified by capillary electrophoresis (Bioanalyzer, Agilent).

The library was clustered on the Illumina cBOT station and sequenced paired end for 101 cycles on the HiSeq 2500 sequencer according to the Illumina cluster and sequencing protocols.

1 - 3 RNA sequencing

1 - 3 - 1 Algal material and RNA isolation

To obtain a wide range of transcripts, RNA was sequenced from various sporophyte tissues maintained under various conditions (Supplementary Table 1). *U. pinnatifida* sporophytes collected fom a long line rope in a culture farm in Wando, Korea on 2015 January 23rd were maintained alive in an icebox and brought to the laboratory. They were cleaned to remove external contaminants. Tissue from the sporangium, the meristem, the blade and the stipe were subsampled and then each subjected to either 1) 12h at 20°C immersed in autoclaved seawater at approx. 600 lux, or 2) 12h at 20°C immersed in autoclaved seawater in total dark. Right after the end of the treatment tissues were frozen in liquid nitrogen and kept at -80°C. Each algal sample was kept frozen and ground on the Automill TK-AM5 frozen crusher (http://www.tokken.jp). Total RNA was extracted from an average of 200 mg of ground tissue following the protocol developed by Ahn et al. (2004), modified by adding chloroform extraction two more times and two RNA washing steps. Total RNA was loaded and resolved through a 1.0% agarose gel in order to check its integrity. After a 30 min electrophoresis at 100V, the gel was stained in a solution of ethidium bromide (0.5 µg/ml) for 30 min and unstained in distilled water. RNA quantification and qualification was performed on the 2100 Expert Bioanalyzer platform (https://www.genomics.agilent.com) using the RNA 6000 Nano Kit (Agilent, CA, USA).

1 - 3 - 2 Isoseq library cDNA and sequencing

Using the SMARTer PCR cDNA Synthesis Kit (Clontech 634925), RNA was synthesized to cDNA. 250 ng of total RNA was used in each cDNA synthesis

reaction. To determine the optimal number of cycles for large-scale PCR, cycle optimization was performed. After large-scale PCR, using the BluePippin size selection system 3 fractions of cDNA (1-2 kb, 2-3 kb, 3-6 kb) were prepared. Each sample was used as a separate input for library preparation. The SMRTbell library was constructed by using SMRTbell™ Template Prep Kit 1.0 (PN 100-259-100). After a sequencing primer was annealed to the SMRTbell template, DNA polymerase was bound to the complex (DNA/Polymerase Binding kit P6). Following the polymerase binding reaction, the MagBead Kit was used to bind the library complex with MagBeads before sequencing. MagBead bound complexes enable for more reads per SMRT Cell. This polymerase-SMRTbell-adaptor complex was then loaded into zero-mode waveguides (ZMWs). The SMRTbell library was sequenced using 16 SMRT cells, 1-2 kb: 4 cells, 2-3 kb: 5 cells, 3-6 kb: 7 cells) (Pacific Biosciences) using C4 chemistry (DNA sequencing Reagent 4.0) and 1 x 240 minute movies were captured for each SMRT cell using the PacBio RS (Pacific Biosciences) sequencing platform.

1 - 3 - 3 Illumina paired-end cDNA library and sequencing

Sequencing libraries were generated from one microgram of total RNA using TruSeq RNA Sample Prep Kit (Illumina) according to the manufacturer's protocol. In brief, the poly-A containing RNA molecules were purified using poly-T oligo attached magnetic beads. After purification, the total poly A+RNA was fragmented into small pieces using divalent cations under elevated temperature. The cleaved mRNA fragments were reverse transcribed into first strand cDNA using random primers. Short fragments were purified with a QiaQuick PCR extraction kit and resolved with EB buffer for end reparation and addition of poly (A). Subsequently, the short

fragments were connected with sequencing adapters. The resulting cDNA libraries were then paired-end sequenced (2x101bp) on the HiSeq™ 2000 (Illumina) platform.

1 - 3 Genome assembly

The genome assembly process is summarized in the Supplementary Figure 1. PacBio complete sequence reads were processed for error correction with SMRT Analysis v2.3. Using the published mitochondrial genome (Li et al., 2015) and plastid genome (Zhang et al., 2016), organellar PacBio reads were filtered out using BWA (Li & Durbin, 2009) and custom perl scripts. Total DNA sequences were subjected to pre-processing steps including adapter trimming, quality trimming and contamination removal for paired-end DNA sequences from Illumina HiSeq2500. Adapter trimming and quality trimming were conducted using Trimmomatic methods (Bolger et al., 2014) with parameter settings like leading:5, trailing:5, sliding window:4:15, and minlen:30. Trimmed sequences were checked for bacterial contamination by mapping them using bowtie2 (Langmead & Salzberg, 2012) against marine metagenome whole genome shotgun (WGS) sequences (Bio Project: PRJNA13694) downloaded from NCBI. Mapped reads were removed with their respective pairs, from now on these sequence are called as pre-processed.

All HiSeq pre-processed sequences were subjected to genome size estimation using the k-mer-based method that was used in the panda genome. The k-mer frequency with 21-mer was obtained using the Jellyfish (Marcais & Kingsford, 2011) method and genome size was calculated (Li et al., 2010). The length of the nuclear genome of *U. pinnatifida* was calculated from k-mer frequency distributions based on the 25.25 Gb of Illumina HiSeq cleaned reads. The nuclear genome size was calculated as the average of the genome sizes obtained for the various k-mer (17; 19; 21 bp) and a

value of 557.41 Mb was obtained (Supplementary Figure 2). This estimate of the nuclear genome size was in accordance with the size estimated using flow cytometry experiments (580 Mb: Le Gall et al., 1993).

Error corrected PacBio long reads were imported to the denovo assembler FALCON-Unzip assembler (Chin et al., 2016). Assembled contigs were further polished with Quiver consensus method to reduce the errors (Chin et al., 2013). Finally, the assembled and polished contigs were subjected to error correction using CLCAssemblyCell v4.2.0 (https://www.qiagenbioinformatics.com/products/clc-assembly-cell/) with pre-processed HiSeq sequences.

Assembly of the cleaned nuclear PacBio long reads was performed using the Falcon-Unzip assembler. A total of 3,876 contigs were assembled for a total length of 633,990,350 bp of the nuclear genome of *U. pinnatifida*. The assembly N50 was 406,301 bp (Supplementary Table 2) and 73 contigs were longer than 1 Mb.

1 - 4 Superscaffolding

The contig assembly was further assembled into pseudochromosomes using a genetic map published by Shan et al. (2015). The data comprised of 103 individuals including 2 parents and 101 progenies for a total of 28.06 Gb of reads. Linkage mapping of this data was conducted with the pipeline Lep-MAP3 (Rastas, 2017).

First, each individual was mapped to the assembled contigs using bowtie2 (Langmead & Salzberg, 2012) using the very-sensitive-end-to-end algorithm to reduce mapping of reads at multiple positions. The average mapping rate was of 71.58%. All mapping files were further trimmed and sorted with SAMtools version 1.5 (Li et al., 2009; Li, 2011) to conserve only the reads mapped with a MAPQ higher than 10. Genotype posterior probabilities were calculated by the Lep-MAP3 pipeline from the output of

samtools mpileup ran on each individual mapping file using default settings. To provide the final dataset for linkage mapping, the module ParentCall2 was used to determine reliable parental genotype of each markers. Then markers were separated into linkage groups using the SeparateChromosome2 module using informative markers only paternally, only maternally and for both parents (informativeMask=123) with a segregation distortion aware LOD scores (distortionLod=1) limit of 16 (lodLimit=16) and a recombination fraction of 0.3 (theta=0.3). This resulted in 30 linkage groups. Single markers that were not included in the linkage groups were further joined to the 30 linkage groups using the JoinSingles2All module using the same setting as for the SeparateChromosome2 module.

Markers were ordered for each linkage group separately using the OrderMarkers2 module using default parameters. The module was run sequentially 5 times to improve likelihood of the order. Each linkage group's map was inspected and corrected manually when needed prioritizing the physical position of the markers. The final likelihood and map length were estimated using the module OrderMarkers2. Contigs were assigned to linkage groups and arranged to form 30 pseudochromosomes. The contigs within pseudochromosomes were spaced by 100 "N".

Using 18,878 markers a genetic map of 30 linkage groups was reconstructed for a total distance of 1,981.72 cM. These results are in accordance with those found by Shan et al. (2015) (i.e. 30 linkage groups for a total distance of 1,816.28 cM). Due to their small size and dot-like structure, the precise number of chromosomes has been historically hard to determine (Lewis, 1996), despite these difficulties, the haploid number of chromosomes in *U. pinnatifida* was estimated to be 30 by different authors (Inoh & Nishibayashi, 1955; Inoh & Nishibayashi, 1960; Ohmori, 1967; Migita,

1967; Yabu et al., 1988) and this number can be regarded as reliable. Therefore, the genetic map was likely to contain the correct number of linkage groups. Taken together these reports indicated the good quality of the genetic map and that it was reasonable to order the contigs based on its information. When assigning the contigs to the linkage groups, 36 contigs showed signs of chimeric assembly (i.e. markers were found in two linkage groups and/or at non-congruent genetic positions). Because of the large genetic distance between the samples used to construct our assembly and the samples used to construct the genetic map (i.e. from China and South Korea, respectively) it was assumed that individuals from those two populations had likely undergone genetic recombination and/or small chromosome rearrangements generating chimers. Therefore, chimeric contigs were not split but the less likely positioning in a linkage group (i.e. position with the smallest number of markers) was removed from the linkage group. The pseudochromosomes reconstructed with the help of this genetic map contained 1,325 contigs for a total length of 461 Mb (Supplementary Table 3). The remaining 2,351 contigs that were not included into the 30 linkage groups were all artificially grouped into a single linkage group (LG00). They were mostly short contigs with a N50 of 81,538 bp (Supplementary Table 4). All together these 30 pseudochromosomes and the remaining contigs formed the Kr2015 nuclear genome of *U. pinnatifida.*

1 - 5 Assembly quality evaluation

The completeness of the Kr2015 assembly was assessed using various methods. First, proteins found in the genomes of *Ectocarpus siliculosus* and *S. japonica* were mapped onto the Kr2015 assembly using exonerate (Slater & Birney, 2005) resulting in the mapping of 14,257 proteins (87.62% of the 16,271 total set) and 15,500 proteins

(82.74% of the 18,733 total set), respectively. The high proportion of the proteins detected in our assembly supports the conclusion that the Kr2015 assembly encompasses most of the genome sequence of *U. pinnatifida*. Second, the completeness of the Kr2015 assembly was also estimated with the core eukaryote gene set (eukaryota_odb9) in the BUSCO pipelines v.1.1 (Simão et al., 2015). The Kr2015 assembly contained 222 full-length CEGs and 14 partial CEGs from BUSCO for a total completeness of 77.88% (Supplementary Table 5). The proportion of missing genes was higher than that observed in glaucophytes (93% CEGs from BUSCO: Price et al., 2019) and red algae (69.9%-88.5% CEGs from BUSCO: Lee et al., 2018) suggesting that the Kr2015 assembly may be incomplete. However, when compared to BUSCO analysis we performed on other brown algae genomes, the Kr2015 assembly was at least as complete as other brown algal genomes (Supplementary Table 5).

1 - 6 Organellar genomes

PacBio raw reads were filtered for organellar reads using BWA (Li & Durbin, 2009) and custom perl scripts against mitochondria and chloroplast sequence generated in previous studies (Li et al., 2015; Zhang et al., 2016). In total, 102,423 organellar reads were isolated and de novo assembled using CANU with the setting genomeSize=400k (Koren et al., 2017). From these assembled contigs, the best matched contig for the mitochondria and chloroplast genomes were identified using blastn (e-value 10e-05). Finally, since the mitochondrial and chloroplast genomes are typically circular, the forms of the contig were asserted using MUMmers plotting and one of the self-similar ends was trimmed to manually create a circular structure. Annotation of the organelle genomes was conducted in Geneious version 8.1.2 (https://www.geneious.com) using

the “Annotate from” option and the previously published organelle genomes as templates.

1 - 7 Genome annotation

1 - 7 - 1 Transposable elements and repetitive elements

Repeat regions of the Kr2015 genome were predicted using *de novo* method and classified into repeat subclasses using reference databases. *De novo* repeat library was predicted using RepeatModeler (http://www.repeatmasker.org/RepeatModeler/) and was annotated using the RepeatClassifier module included in RepeatModeler. Repeat sequences having no similarity with the reference databases were further analyzed using blastx against the NCBI Non-redundant protein database (e-value cutoff 10e-20) as they might represent tandemly repeated genes. A total of 818 of these repeats were shown to have identity to known protein sequences and were removed from the repeat library. The final predicted repeats library containing 13,012 sequences was used to mask the Kr2015 genome using RepeatMasker v4.0.7 (http://www.repeatmasker.org/cgi-bin/WEBRepeatMasker) with the engine rmblast v2.2.27+. Similar procedure was conducted on the genomes of *S. japonica* and *E. siliculosus*.

1 - 7 - 2 Gene prediction

An in-house gene prediction pipeline was constructed using three steps: evidence-based gene modeller, ab-initio gene model and consensus gene model (Supplementary Figure 4). Finally, the transcripts and genes from the consensus gene model were subjected to functional annotation. These annotation pipelines are explained in the

methods section of *Capsicum* (Kim et al., 2014) and *Haliotis* (Nam et al., 2017) structural genome annotation. Initially, sequenced transcriptomes from Illumina were mapped to the *U. pinnatifida* repeat masked reference genome using Tophat2 (Kim et al., 2013) and gene structural boundaries were predicted using Cufflink (Trapnell et al., 2010; Roberts et al., 2011) and PASA (Haas et al., 2003). Orthologous reference genomes were manually selected and included *Arabidopsis thaliana*, *E. siliculosus*, *Nannochloropsis oceanica*, *Phaeodactylum tricornutum*, *Thalassiosira pseudonana*, and *S. japonica*, algae proteins of uniprot and plant transcription factor proteins. Protein sequences from these genomes were mapped to the masked genome of *U. pinnatifida* using Exonerate (Slater & Birney 2005). For ab initio gene prediction, Augustus was trained using RNA-seq data and known proteins using the complete transcriptome as training matrix for HMM. Gene prediction data from each method was combined using EVidenceModeler (Haas et al., 2008) to build a consensus gene set for the genome. The consensus gene models were manually curated using in-house python script to reduce false-positive predictions. The pipeline predicted 20,716 complete protein-coding gene. The majority (94.31%) of these gene models were supported by transcriptome and/or protein evidence. The 20,716 complete protein-coding genes were subjected to functional annotation by obtaining the ontologies from reference databases (NCBI - NR databases, swiss-prot, gene ontologies and KEGG pathways) using the Blast2GO method (Götz et al., 2008).

2 - Comparative genomics

2 – 1 Role of the transposable elements in the genome size determination

The repeatome of *U. pinnatifida* constituted 52.10% of its genome, of which at least 19.14% were TEs, representing 121 Mb of the genome. The majority of TEs were

retrotransposons and notably, Long-Terminal Repeats (LTRs) of the superfamilies Copia and Gypsy (3.64% and 6.49%, respectively). Other retrotransposons of the orders LINEs and SINEs represented a smaller proportion of the genome (4.85%) and DNA transposons an even smaller proportion (2.35%). This was in accordance with the repeats content reported in Shan et al. (2020).

This repeatome was comparable to that of the *S. japonica* genome, with the repeatome covering 48.23% of the genome, with TEs composing 12.41% of the genome (67 Mb) with the proportion difference in TEs explained by the higher numbers of LTRs in the Kr2015 genome (Supplementary Table 6). On the other hand, the repeatome of *E. siliculosus* constituted only 31.50% of its genome and the TEs 12.15%. Interestingly, this expansion of the repeatome appeared to be correlated with a length expansion of the genome in the Laminariales (543-635 Mb) compared to the length of the genome in the Ectocarpales (169-196 Mb).

To investigate this question, the JC distance between the consensus sequence of each element and their respective insertions in the three genomes was used as an estimate of the insertion time of the repeats. For each transposable element present in the genome's repeat library, genetic distance between each repeat copies found in the assembly and their respective consensus sequence were parsed from the RepeatMasker output and used to calculate the Jukes-Cantor distance (Jukes & Cantor, 1969; JC) using the formula: d = -(3/4)loge(1-(4/3)p), where p is the genetic distance between a repeat copy to its consensus. The two Laminariales species showed a peak of insertions for JC-distance values between 0.04-0.06, which was not present in *E. siliculosus* (Supplementary Figure 5). Because this peak was placed at low JC-values it is reasonable to estimate that it happened after the split of the Ectocarpales and Laminariales. Furthermore, comparison between *U. pinnatifida* and

S. japonica showed that if the profile of insertions was similar it varied by the intensity of the insertions and was totally different for JC-distance values of 0-0.01. Both differences were explained by the much higher insertion rate of LTRs elements in *U. pinnatifida* with around twice as many of them inserted with JC-distances ranging from 0.02 to 0.15 (Supplementary Figure 5). These results support the importance of TE insertions to explain the expansion of the genome in species of the order Laminariales. Similarly, the role of TEs in the determination of genome size has been widely recognized, notably in plants and animals (Bennetzen, 2002; Kidwell, 2002; Kazazian, 2004, Feschotte & Pritham, 2007; Sessegolo et al., 2016) and more recently in red algae (Lee et al., 2018). However, due to the narrow range of taxon sampling in this study, the pattern of genome size variation in the Phaeophyceae needs to be further characterized with the addition of more genomes.

2 - 2 Genome organisation of the brown algae

With the *E. siliculosus* pseudochromosomes reconstructed and annotated (Cormier et al., 2017) and the ones from the Kr2015 *U. pinnatifida* genome, the structure of the brown algal chromosomes were investigated by inspecting the density of genes and repeats along the pseudochromosomes. Gene density compared between the genomes was significantly different (Wilcoxon rank sum test p-value < 2.2e-16) and was almost two-times higher in the genome of *E. siliculosus* compared to the Kr2015 genome of *U. pinnatifida* (Figure 1). These observations were explained by the transposable elements driven genome size expansion in *U. pinnatifida* that was not accompanied by an expansion in gene number (Supplementary Table 7), therefore spreading the same number of genes on larger chromosomes.

Furthermore, in both species the genes generally showed a homogeneous distribution along all pseudochromosomes with only a few 1 Mb windows (i.e. 10 in *E. siliculosus* and 21 in *U. pinnatifida*) showing a gene density statistically lower than the genome background (below 1.5*IQR based on Tukey's method). The gene and repeat densities in the genome reported in Shan et al. (2020) appeared to follow a similar homogeneous distribution at the exception of their chromosome 3. This observation suggests that the insertion of transposable elements during the genome expansion in the Laminariales was random and occurred at equal frequencies everywhere in the genome.

2 - 3 Orthologous analysis and Dollo parsimony analysis

To study the evolution of gene content in the Laminariales as well as in brown algae, genome data from 18 taxa representing the diversity of the stramenopiles were gathered and compared to that of the Kr2015 genome (Supplementary Table 15). Ortologous analysis were conducted with Orthofinder (Emms & Kelly, 2015) and clustered the 357,280 genes into 46,492 gene families (Supplementary Table 15), the largest one grouped 297 genes across the 19 species. Out of these orthologous gene families. 459 orthologous single genes were aligned using MAFFT version6 using the G-INS-i strategy and with an offset value of 0.1 (Katoh & Toh, 2008). Maximum likelihood reconstruction was conducted with IQ-Tree v1.6.9 (Nguyen et al., 2015) with independent substitution model for each single genes determined with the -m TEST option. Branch supports were obtained with the ultrafast bootstrap (UFBoot) implemented in IQ-Tree with 1000 replications (Hoang et al., 2018). The phylogenetic tree was rooted between the photosynthetic stramenopiles (Ochrophyta) and the non-photosynthetic stramenopiles (Supplementary Figure 12). This

phylogenetic tree was used as a framework for Dollo parsimony analysis. The results show that multiple gene inventory expansions occurred during the evolution of the stramenopiles, starting after the split of the photosynthetic and non-photosynthetic lineages, that gained 2,812 and 2,773 gene families, respectively. From this point onward, the gene repository in the photosynthetic stramenopiles underwent important shuffling and specialization, reflected in the important gene families' shuffling (e.g. in the common ancestor of diatoms). Strikingly the brown algae showed a major expansion of their gene repository with 3,543 gene families gained and 756 loss for a net gain of 2,787 gene families (Supplementary Figure 12-13).

GO term enrichment analysis were conducted using Fisher's exact test implemented in the R library TopGO (Alexa & Rahnenfuhrer, 2020) with all annotated genes in the Kr2015 genome as background and different subsets of the Kr2015 genes as foreground.

The genes encoded in the 3,543 gene families gained in the common ancestor of the brown algae were used as the foreground subset in GO enrichment analysis. The most significantly enriched GO terms (Fisher's test p-value < 0.05) were related to the membrane biology, and notably synthesis of some of its component (Supplementary Table 16). Consistently with what Ye et al. (2015) reported, these gene families were mainly involved in the cell wall biosynthesis of the brown algae, such as cellulose synthase, mannuronan C-5-epimerase, the alpha-(1,6)-fucosyltransferase and pectin lyase (Michel et al., 2010). Other families that were gained in the brown algae included leucine-rich GTPase, imm upregulated genes, WD40 repeat-containing genes, insulin-like growth factor, lipoxygenase, Notch domain containing genes, or SET domain containing genes, families that might have a role in defence, development and growth (Peters et al., 2008; Roy Choudhury et al., 2010; Vera et al.,

2011; Zambounis et al., 2012). Interestingly, some of these gene families, notably the pectin lyase families, were also expanded in the genome of *Ulva mutabilis*, a green seaweed, suggesting convergent evolution between these lineages and further functional investigations on these gene families should be conducted to investigate their role during the establishment of multicellularity (De Clerck et al., 2018).

In the common ancestor of the Laminariales, the 869 gained gene families were significantly enriched for GO terms (Fisher's test p-value < 0.05) related to transcriptional regulatory functions (Supplementary Table 18). This result suggest that the differentiation between the orders of brown algae might be linked to changes in expression regulatory network more than in the gain of new functions, Furthermore, the level of expansion of key gene families was not uniform across the four brown algal species and in general (10 out of 32 gene families [chi square test p-value < 0.05]) the Laminariales contained more copies of these genes suggesting that the increased body size and relative complexity of these algae over the Ectocarpales was correlated to the expansion of a few key gene families (Supplementary Figure 14).

The significantly enriched GO terms (Fisher's test p-value < 0.05) in the 808 specific to the Kr2015 genome did not clearly lean toward a category (Supplementary Table 12). However, a number of "responses" and "defence" terms were significantly enriched, that could suggest that in *U. pinnatifida* there has been species-specific adaptation to environmental and biotic interactions.

Overall, these results could suggest that the evolution of the brown algae was marked by the acquisition of a large number of functions in their common ancestor and that the different lineages gained specific transcription regulation network of these functions. However, based only on four genomes from two of the 20 orders in brown

algae, this hypothesis remains extremely weak. Large sequencing effort of brown algae genomes is required to deepen our understanding of the evolution of the brown algae.

2 - 3 Synteny analysis

Syntenic blocks were identified using MCScanX (Wang et al., 2012) between 1) the Kr2015 and the Shan et al. (2020) gene models and 2) the Kr2015 and *E. siliculosus* gene models. The minimum syntenic block length was set to 5 genes and the maximum gap between genes in a syntenic block was set to 25 genes. The results were visualized using the R package circlize (Gu et al., 2014).

The synteny analysis between the two assemblies of the *U. pinnatifida* genome showed that 15 pseudochromosomes were exclusively linked, meaning that they shared synteny with only one pseudochromosome (Supplementary Figure 3; e.g. Kr2015 LG03 with the HiC_scaffold_21). The number of pseudochromosomes not exclusively linked (i.e. 13) was surprisingly high for assemblies of the same species. However, out of these non-exclusive pseudochromosomes, eight of them shared almost exclusively with another and only shared a single syntenic block with another pseudochromosome. These could have resulted from the different assembly methodologies (i.e. genetic map superscaffolding for Kr2015 vs HiC scaffolding) or could represent small genome. Furthermore, the pseudochromosme LG05 shared syntenic blocks only with the HiC_scaffold_30 and a HiC_scaffold not included in the 30 chromosomes of *U. pinnatifida* (HiC_scaffold_108), bringing the number of almost exactly shared pseudochromosome to 24. The remaining 6 pseudochromosomes showed complex fusion/split patterns probably resulting from

assembly's methodologies and the discussion of which goes beyond the scope of our study.

The synteny analysis between the pseudochromosomes of Kr2015 and *E. siliculosus* showed that 16 pseudochromosomes were exclusively linked (Figure 1; e.g. KR2015 LG05 and *E. siliculosus* chr_01). The difference in karyotype number (28 chromosomes in *E. siliculosus* and 30 *U. pinnatifida*) was explained by four cases of split/fusion. They occurred between two Kr2015 pseudochromosomes and one *E. siliculosus* pseudochromosome in three cases (Figure 1; Kr2015 LG08 - LG09 and *E. siliculosus* chr_03; Kr2015 LG18 - LG30 and *E. siliculosus* chr_05; Kr2015 LG21 - LG29 and *E. siliculosus* chr_06). They occurred between one Kr2015 pseudochromosome and two *E. siliculosus* pseudochromosomes in one case (Figure 1; Kr2015 LG11 and *E. siliculosus* chr_13 and chr_25). The remaining seven pseudochromosmes of both species formed two complex events of partial chromosomes fusion/split(s) (Supplementary Figure 15).

Interestingly, despite the general high conservation between the chromosomes of Kr2015 and *E. siliculosus*, the loss of synteny with the pseudochromosome chr_13 of *E. siliculosus* in Kr2015 was striking (Figure 1). Interestingly, this pseudochromosome corresponds to the sex chromosome of *E. siliculosus* and contains the Sex Determining Region (SDR) of this species (Ahmed et al., 2014; Cormier et al., 2017) and this loss of synteny was in accordance with the rapid evolution of the sex related loci in brown algae (Lipinska et al., 2017).

3 - Population genomics

3 - 1 Algal material

For the population analyses, 41 individuals were sampled from eight populations located in Korea, France and New Zealand (Figure 2 [maps generated by GMT 5.4.5; Wessel et al., 2013] and Supplementary Table 8).

In Korea, mature sporophytes from Tongyeong (34°50'07.9"N 128°24'01.5"E) were collected on December 29th 2017 and mature sporophytes from Goseong (38°17'45.2"N 128°33'01.4"E) were collected on January 4th 2018. In both of these populations,

All the mature sporophytes from Wando (approx. 34°19'30.2"N 126°39'05.5"E) were collected on longlines from an *U. pinnatifida* farm on January 23rd 2015 and April 4th 2017. After collection, sporophytes of *U. pinnatifida* were washed in autoclaved seawater, dried with paper towels, and preserved in silica gel.

In France, sporophytes were collected on April 19th 2016 from the Thau lagoon (Mediterranean Sea) from rocky habitats (3m depth) nearby the Roquerols lighthouse (43°25'49.8"N 3°40'20.2"E), and in the Bloscon marina in Roscoff (Brittany) along pontoons on April 5th, 2016. Samples were dried with paper towels and preserved into silica gel renewed twice before shipment.

In New Zealand, samples were collected January 9th 2017 from the upper subtidal zone, growing on rock and concrete surfaces in Oriental Bay, Wellington Harbour (within 50m of the site where *Undaria pinnatifida* was first recognised in New

Zealand), and in Lyall Bay (on the Cook Strait coast of Wellington) on January 12th 2016. Samples were rinsed with clean seawater, dried with paper towels and preserved in silica gel.

Additionally, voucher specimens preserved in 1987, the first ever recorded in New Zealand (Hay & Luckens, 1987) were obtained from the Museum of New Zealand Te Papa Tongarewa.

3 - 2 DNA isolation and Illumina paired-end sequencing

DNA was isolated from 20 to 50 mg of dried blade tissue for each individual using the GeneAll Exgene Plant SV Minin Kit (GeneAll Biotechnology, Korea). Due to the high polysaccharide content in *U. pinnatifida*, lysis was conducted using a double amount of PL and PD Buffer. Subsequent steps were conducted according to manufacturer's instructions. Finally, two cleaning steps were conducted using the PowerClean® DNA Clean-Up Kit (Qiagen, Carlsbad, CA).

DNA libraries were prepared according to Illumina Truseq Nano DNA Library prep protocol. For sample library preparation, 0.2 μg for insert 550 bp size of high molecular weight genomic DNA were randomly sheared to yield DNA fragments using the Covaris S2 system. The fragments were blunt ended and phosphorylated, and a single 'A' nucleotide was added to the 3' ends of the fragments in preparation for ligation to an adapter that has a single-base 'T' overhang. Adapter ligation at both ends of the genomic DNA fragment conferred different sequences at the 5' and 3' ends of each strand in the genomic fragment. Ligated DNA was PCR amplified to enrich for fragments that have adapters on both ends. The quality of the amplified libraries was verified by capillary electrophoresis (Bioanalyzer, Agilent). The library was clustered

on the Illumina cBOT station and sequenced paired end for 101 cycles on the HiSeq 2500 sequencer according to the Illumina cluster and

3 - 3 Read mapping and variant calling

Total DNA sequences were subjected to pre-processing steps including adapter trimming, quality trimming and contamination removal for paired-end DNA sequences from Illumina HiSeq2500. Adapter trimming and quality trimming were conducted using Trimmomatic methods (Bolger et al., 2014) with the following parameter settings: leading 5, trailing 5, sliding window 4:15, and minlen 30. To detect variants in each individual, the methods described in Van der Auwera et al. (2013) were followed. Rapidly, for each individual the following procedure was performed: (1) insert size of the sequencing library was estimated during library construction. (2) Trimmed forward (R1) and reverse (R2) reads were mapped to the unmasked genome of *U. pinnatifida* (version 1.0) using bowtie-2 version 2.2.6 using the local-very-sensitive alignment and the -I and -X estimated earlier (Langmead & Salzberg, 2012). (3) Mapped reads were sorted with SAMtools version 1.5 (Li et al., 2009). (4) Duplicated reads were marked with picard version 2.9.2 (http://broadinstitute.github.io/picard/). (5) Local realignment around indels was performed using the IndelRealigner function of the Genome Analysis Toolkit GATK.3.8-0 (DePristo et al., 2011).

For each individual a general variant calling file (gVCF) was constructed with the HaplotypeCaller function of GATK.3.8-0 with the following parameters: --genotyping_mode DISCOVERY --emitRefConfidence GVCF --ploidy 2. Individual gVCF files were combined by the GenotypeGVCFs function of GATK.3.8-0 to form a single variant calling file (VCF). The total VCF file was split by type of variant (i.e.

SNP, INDEL). A hard filtering of the variants was carried out following guidelines provided by the Broad institute (https://gatkforums.broadinstitute.org/gatk/discussion/2806/howto-apply-hard-filters-to-a-call-set). The filters applied were as follows: (QD < 2.0), Fisher strand bias (FS > 60.0), mapping quality (MQ < 40.0), (MQRankSum < -12.5), (ReadPosRankSum < -8.0), minimum coverage (DP < 50) and maximum coverage (DP > 1500). Furthermore, variants were trimmed for a minimum allele frequency of 0.0365 and no missing genotyping, using a combination of plink v1.9 (Purcell et al., 2007) and vcftools v0.1.15 (Danecek et al., 2011). The final variants dataset was annotated with SnpEff v4.3 (Cingolani et al., 2012).

3 - 4 Genome polymorphism across individuals

3 - 4 - 1 Principal Component Analysis

The combined dataset of 6,123,124 SNPs and 1,130,417 INDELs variants was used to calculate principal components using plink v1.9 (Purcell et al., 2007).

3 - 4 - 2 Phylogenetic tree

The maximum-likelihood tree of the 41 individuals was reconstructed using SNPhylo (Lee et al., 2014) with 100 bootstrap replicates and default parameters. After SNPhylo internal filtration steps 2,384 biallelic SNPs were used to reconstruct the tree.

3 - 4 - 3 Admixture analysis

For admixture analysis, we took advantage of the *snmf* algorithm implemented in the R package LEA (Frichot & François, 2014). Because the *snmf* algorithm does not

make assumptions on Hardy-Weinberg equilibrium it is particularly suited for a highly selfing species like *U. pinnatifida*. The *snmf* function was run on the biallelic SNPs and INDELs (totalling 7,186,271 variants) with 100 repetitions and default parameters for K values comprised between 1 and 11.

3 - 5 Genome landscape

3 - 5 - 1 Genetic diversity estimators

The expected heterozygosity (H_e), nucleotide diversity π and fixation index (F_{IS}) in the nine populations were calculated for the 6,123,124 SNPs using the "population" module of the stacks v1.48 pipeline (Catchen et al., 2011). The calculation in sliding-windows was conducted in vcftools 1.15 (Danecek et al., 2011) for different length of non-overlapping windows.

3 - 5 - 2 Linkage disequilibrium

Linkage disequilibrium (LD) holds interesting information on the population history, population size (e.g., founder events) and reproductive system (e.g., selfing) (Ardlie et al., 2002; Amaral et al., 2008; Glémin et al., 2019). LD was estimated from r^2 for pairs of SNPs (Hill & Robertson, 1968). The linkage disequilibrium analysis was conducted in PopLDdecay v3.40 (Zhang et al., 2019) with default parameters. The analysis was conducted on the variants (SNPs and INDELs) detected on the 30 pseudochromosomes.

3 - 5 - 3 Run of homozygosity

Run of homozygosity (ROH) were identified with plink v1.9 (Purcell et al., 2007) with sliding window of 500 Kb (--homozy-window-kb 500) with 20 heterozygotes loci allowed in a window (--homozyg-window-het 20) and a proportion of overlapping window of 0.025 (--homozyg-window-treshold 0.025). Because genetic drift can be a strong force in small populations and can increase the length of the ROH[43-44], we also performed comparisons between populations when only considering ROH longer than 1.5 Mb region and obtained similar results as with all ROH (Supplementary Table 10).

Genome heterozygosity was estimated for each individual as the proportion of heterozygote variants in the genome. For each individual, the 6,123,124 high quality SNP were used to generate a fasta file for each sample with the GATK FastaAlternateReferenceMaker (version 3.8-0) with the IUPAC codes to represent heterozygous loci. The genome heterozygosity of each individual was estimated by dividing the total number of IUPAC coded positions by the total number of positions in the fasta file. As expected, the level of heterozygosity and the ROH coverage were strongly correlated (pearson correlation r = -0.9379, p-value < 2.2e-16) in all individuals (Extended Data Figure 3). Interestingly, the ROH and heterozygosity variability between the cultivated individuals (excluding CUL_Kr_Wando2015_4) was much lower than between the natural individuals, maybe the reflection of the population size difference between these types of populations. Further supporting this idea were the individuals introduced in New Zealand that showed the lowest variability. However, because the introduced populations in France had levels of variability comparable to that of natural populations for a much lower effective population size, this suggests that this variability might not be the result of solely

demographic effects. The reproductive system of *U. pinnatifida*, or a combination of both factors could help explain this observation.

Principal component analysis calculated on the different ROH statistics (i.e. Number of ROH, total length of ROH, average length of ROH; Supplementary Table 10) of each individual revealed similar clustering. The cultivated and New Zealand introduced populations formed tight clusters whereas the natural and French introduced populations formed looser clusters. Interestingly, the individuals sampled in 1987 and 2017 from the introduced population in Wellington (New Zealand) were clearly distinguished in two separate clusters on the principal component 1 and 2 (Extended Data Figure 3).

3 - 6 Selection

Signals of selection were detected by combining three statistics calculated in 50 kb sliding windows along the Kr2015 genome of *U. pinnatifida*. We used the (1) reduction of diversity (ROD) calculated as ROD = 1 - (π_{der} / π_{anc}); (2) the delta Tajima's D calculated as $\Delta T_D = T_{D\text{-}anc} - T_{D\text{-}der}$; and (3) the population differentiation (F_{ST}). The statistics were combined using the decorrelated composite of multiple signals method (DCMS; Ma et al., 2015).

Tajima's D (Supplementary Figure 16), F_{ST} (Supplementary Figure 17) and π were estimated in non-overlapping 50 kb windows in all populations for the combined SNPs and INDELs dataset using vcftools 1.15 loci (Danecek et al., 2011) and a minimum allele frequency (-maf) equal to 1 / (2 x number of individuals) to exclude monomorphic loci. For each statistic we tested if its distribution fitted the normal distribution, and, as none did, we performed a two-step normalization approach (Templeton, 2011). From each of the *z*-scores distribution obtained we derived a *p*-

value. The correlation of the *p*-value of each statistics were calculated in R (R Core Team, 2020) and used to calculate their respective weight factors (Supplementary Table 19). Finally for each window the DCMS was estimated and a *p*-value was derived for each window following the similar method described above. Regions under putative positive selection were defined as the windows with a *p*-value < 0.025. GO term enrichment analysis were conducted using Fisher's exact test implemented in the R library TopGO (Alexa & Rahnenfuhrer, 2020) with all annotated genes in the Kr2015 genome as background and genes encoded in regions under putative positive selection as foreground.

The analysis of natural and cultivated identified 224 (107 in the contigs not assigned to the pseudochromosomes) genomic windows putatively under selection that had a significant DCMS score (*p*-value < 0.025). The average length of these regions was of 70.2 kb. They were found in all the linkage groups of *U. pinnatifida*, with the largest region covering 450 kb on the pseudochromosome LG16 (Figure 4a). These regions encoded 508 genes of which 292 (57.5%) were functionally annotated. Gene Ontology (GO) analysis of these genes revealed that they were enriched in several biological processes such as glycolipid biosynthesis and cytokinetic process (Supplementary Table 12).

The analysis of the 1987 and 2017 Wellington Harbour individuals revealed 252 (112 in the contigs not assigned to the pseudochromosomes) genomic windows putatively under selection (average length 62.9 kb; longest region 350 kb on non-assigned pseudochromosome LG12) under selection (*p*-value < 0.025) in Wellington Harbour (Figure 4c). These regions encoded 511 genes that were enriched in biological process such as intracellular signal transduction, cellular response to stimulus or homeostatic process (Supplementary Table 14).

To further investigate the effect of domestication in two brown algae, a comparison of the genes reported to be under selection in domesticated individuals by Ye et al. (2015) with the genes encoded in regions under putative positive selection was conducted by blast (*e*-value cutoff 10e-50). The comparison revealed that out of the 508 genes in *U. pinnatifida* and 714 in *S. japonica*, only 22 genes were found in both species.

Using the RNA sequencing data generated for annotation of the Kr2015 genome (see 1-3), we investigated the expression of genes encoded in the genomic regions under putative positive selection. We first mapped the cleaned RNA reads for each of the eight libraries to the reference gene models using RSEM v1.3.3 (Li & Dewey, 2011) and the Transcripts Per Million (TPM) of each gene was estimated for each library. We then compared the expression level in the orthologous groups with at least one copy encoded in a genomic region under putative selection and one copy encoded outside of this region. Out of the 166 orthologous groups under consideration, 94 did not show an expression difference between the genes (Wilcoxon rank sum test p-value > 0.05). In the remaining 72 orthologous groups, expression appeared to be different between copy(ies) encoded in a genomic region under putative selection and copy(ies) encoded elsewhere on the genome (Wilcoxon rank sum test p-value < 0.05 (Figure 4). This analysis only incorporated data obtained from a single individual, and from different tissues submitted to different treatments (see 1-3). It therefore does not represent a proper comparative analysis of gene expression. However, these results suggest that genes under positive selection might display expression differences when compared to neighbouring genes. A genome-wide association study and transcriptomic analysis should be conducted to clearly identify such loci and their effect on the phenotypes of *Undaria pinnatifida*

References

Ahmed, S. *et al.* A Haploid System of Sex Determination in the Brown Alga *Ectocarpus* sp. *Current Biology* **24**, 1945-1957 (2014).

Ahn, J.-S. *et al.* Optimization of RNA Purification Method from *Ecklonia cava* Kjellman (Laminariales, Phaeophyceae). *ALGAE* **19**, 123-127 (2004).

Alexa, A. & Rahnenfuhrer, J. topGO: Enrichment Analysis for Gene Ontology. R package version 2.40.0. (2020).

Amaral, A. J., Megens, H.-J., Crooijmans, R. P. M. A., Heuven, H. C. M. & Groenen, M. A. M. Linkage disequilibrium decay and haplotype block structure in the pig. *Genetics* **179**, 569-579 (2008).

Ardlie, K. G., Kruglyak, L. & Seielstad, M. Patterns of linkage disequilibrium in the human genome. *Nature Reviews Genetics* **3**, 299-309 (2002).

Bennetzen, J. L. Mechanisms and rates of genome expansion and contraction in flowering plants. *Genetica* **115**, 29-36 (2002).

Bolger, A. M., Lohse, M. & Usadel, B. Trimmomatic: a flexible trimmer for Illumina sequence data. *Bioinformatics* **30**, 2114-2120 (2014).

Catchen, J. M., Amores, A., Hohenlohe, P., Cresko, W. & Postlethwait, J. H. *Stacks* : Building and Genotyping Loci *De Novo* From Short-Read Sequences. *G3: Genes|Genomes|Genetics* **1**, 171-182 (2011).

Chin, C.-S. *et al.* Nonhybrid, finished microbial genome assemblies from long-read SMRT sequencing data. *Nature Methods* **10**, 563-569 (2013).

Chin, C.-S. *et al.* Phased diploid genome assembly with single-molecule real-time sequencing. *Nature Methods* **13**, 1050-1054 (2016).

Cingolani, P. *et al.* A program for annotating and predicting the effects of single nucleotide polymorphisms, SnpEff: SNPs in the genome of *Drosophila melanogaster* strain w^{1118} ; iso-2; iso-3. *Fly* **6**, 80-92 (2012).

Cormier, A. *et al.* Re-annotation, improved large-scale assembly and establishment of a catalogue of noncoding loci for the genome of the model brown alga *Ectocarpus*. *New Phytologist* **214**, 219-232 (2017).

Danecek, P. *et al.* The variant call format and VCFtools. *Bioinformatics* **27**, 2156-2158 (2011).

De Clerck, O. *et al.* Insights into the Evolution of Multicellularity from the Sea Lettuce Genome. *Current Biology* **28**, 2921-2933.e5 (2018).

DePristo, M. A. *et al.* A framework for variation discovery and genotyping using next-generation DNA sequencing data. *Nature Genetics* **43**, 491-498 (2011).

Emms, D. M. & Kelly, S. OrthoFinder: solving fundamental biases in whole genome comparisons dramatically improves orthogroup inference accuracy. *Genome Biology* **16**, (2015).

Feschotte, C. & Pritham, E. J. DNA Transposons and the Evolution of Eukaryotic Genomes. *Annual Review of Genetics* **41**, 331-368 (2007).

Frichot, E. & François, O. LEA: An R package for landscape and ecological association studies. *Methods in Ecology and Evolution* **6**, 925-929 (2015).

Glémin, S., François, C. M. & Galtier, N. Genome evolution in outcrossing vs. selfing vs. asexual species. In *Evolutionary Genomics* (ed. Anisimova, M.) vol. 1910 331-369 (Springer New York, 2019).

Götz, S. *et al.* High-throughput functional annotation and data mining with the Blast2GO suite. *Nucleic Acids Research* **36**, 3420-3435 (2008).

Gu, Z., Gu, L., Eils, R., Schlesner, M. & Brors, B. Circlize implements and enhances circular visualization in R. *Bioinformatics* **30**, 2811-2812 (2014).

Haas, B. J. *et al.* Improving the *Arabidopsis* genome annotation using maximal transcript alignment assemblies. *Nucleic Acids Res.* **31**, 5654-5666 (2003).

Haas, B. J. *et al.* Automated eukaryotic gene structure annotation using EVidenceModeler and the Program to Assemble Spliced Alignments. *Genome Biology* **9**, R7 (2008).

Hay, C. H. & Luckens, P. A. The Asian kelp *Undaria pinnatifida* (Phaeophyta: Laminariales) found in a New Zealand harbour. *New Zealand Journal of Botany* **25**, 329-332 (1987).

Hill, W. G. & Robertson, A. Linkage disequilibrium in finite populations. *Theoretical and Applied Genetics* **38**, 226-231 (1968).

Hoang, D. T., Chernomor, O., von Haeseler, A., Minh, B. Q. & Vinh, L. S. UFBoot2: Improving the Ultrafast Bootstrap Approximation. *Molecular Biology and Evolution* **35**, 518-522 (2018).

Inoh, S. & Nishibayashi, T. (1955). On the mitosis in the sporangium of *Undaria pinnatifida* (Harv.) Sur. *La Kromosomo* **22-24**, 788-793 (1955).

Inoh, S. & Nishibayashi, T. On the mitosis in the sporangium of *Undaria pinnatifida* (Harv.) Sur. (Addendum). *La Kromosomo* **44/45,** 1498-1499 (1960).

Jukes, T. H. & Cantor, C. R. Evolution of Protein Molecules. in *Mammalian Protein Metabolism* 21-132 (Elsevier, 1969).

Katoh, K. & Toh, H. Recent developments in the MAFFT multiple sequence alignment program. *Briefings in Bioinformatics* **9**, 286–298 (2008).

Kazazian, H. H. Mobile Elements: Drivers of Genome Evolution. *Science* **303**, 1626-1632 (2004).

Kidwell, M. G. Transposable elements and the evolution of genome size in eukaryotes. *Genetica* **115**, 49-63 (2002).

Kim, D. *et al.* TopHat2: accurate alignment of transcriptomes in the presence of insertions, deletions and gene fusions. *Genome Biology* **14**, R36 (2013).

Kim, S. *et al.* Genome sequence of the hot pepper provides insights into the evolution of pungency in *Capsicum* species. *Nature Genetics* **46**, 270-278 (2014).

Koren, S. *et al.* Canu: scalable and accurate long-read assembly via adaptive *k* -mer weighting and repeat separation. *Genome Research* **27**, 722-736 (2017).

Langmead, B. & Salzberg, S. L. Fast gapped-read alignment with Bowtie 2. *Nature Methods* **9**, 357-359 (2012).

Lee, J. *et al.* Analysis of the Draft Genome of the Red Seaweed *Gracilariopsis chorda* Provides Insights into Genome Size Evolution in Rhodophyta. *Molecular Biology and Evolution* **35**, 1869-1886 (2018).

Lee, T.-H., Guo, H., Wang, X., Kim, C. & Paterson, A. H. SNPhylo: a pipeline to construct a phylogenetic tree from huge SNP data. *BMC Genomics* **15**, 162 (2014).

Le Gall, Y., Brown, S., Marie, D., Mejjad, M. & Kloareg, B. Quantification of nuclear DNA and G-C content in marine macroalgae by flow cytometry of isolated nuclei. *Protoplasma* **173**, 123-132 (1993).

Lewis, R. J. Chromosomes of the brown algae. *Phycologia* **35**, 19–40 (1996).

Li, H. *et al.* The Sequence Alignment/Map format and SAMtools. *Bioinformatics* **25**, 2078-2079 (2009).

Li, H. A statistical framework for SNP calling, mutation discovery, association mapping and population genetical parameter estimation from sequencing data. *Bioinformatics* **27**, 2987-2993 (2011).

Li, B. & Dewey, C. N. RSEM: accurate transcript quantification from RNA-Seq data with or without a reference genome. *BMC Bioinformatics* **12**, (2011).

Li, H. & Durbin, R. Fast and accurate short read alignment with Burrows-Wheeler transform. *Bioinformatics* **25**, 1754-1760 (2009).

Li, R. *et al.* The sequence and de novo assembly of the giant panda genome. *Nature* **463**, 311-317 (2010).

Li, T.-Y. *et al.* Complete mitochondrial genome of *Undaria pinnatifida* (Alariaceae, Laminariales, Phaeophyceae). *Mitochondrial DNA* **26**, 953-954 (2015).

Lipinska, A. P. *et al.* Multiple gene movements into and out of haploid sex chromosomes. *Genome Biology* **18**, (2017).

Ma, Y. *et al.* Properties of different selection signature statistics and a new strategy for combining them. *Heredity* **115**, 426-436 (2015).

Marçais, G. & Kingsford, C. A fast, lock-free approach for efficient parallel counting of occurrences of k-mers. *Bioinformatics* **27**, 764-770 (2011).

Michel, G., Tonon, T., Scornet, D., Cock, J. M. & Kloareg, B. The cell wall polysaccharide metabolism of the brown alga *Ectocarpus siliculosus*. Insights into the evolution of extracellular matrix polysaccharides in Eukaryotes. *New Phytologist* **188**, 82-97 (2010).

Migita, S. Studies on artificial hybrids between *Undaria peterseniana* (Kjellm.) Okam. and *U. pinnatifida* (Harv.) Sur. Bulletin of the Faculty of Fisheries, Nagasaki University **24**: 9-20. (1967).

Nam, B.-H. *et al.* Genome sequence of pacific abalone (*Haliotis discus hannai*): the first draft genome in family Haliotidae. *GigaScience* **6**, (2017).

Nguyen, L.-T., Schmidt, H. A., von Haeseler, A. & Minh, B. Q. IQ-TREE: A Fast and Effective Stochastic Algorithm for Estimating Maximum-Likelihood Phylogenies. *Molecular Biology and Evolution* **32**, 268-274 (2015).

Ohmori, T. (1967). Morphogenetical studies on Laminariales. Biological Journal of Okayama University. 13: 23-84.

Peters, A. F. *et al.* Life-cycle-generation-specific developmental processes are modified in the immediate upright mutant of the brown alga *Ectocarpus siliculosus*. *Development* **135**, 1503-1512 (2008).

Purcell, S. *et al.* PLINK: A Tool Set for Whole-Genome Association and Population-Based Linkage Analyses. *The American Journal of Human Genetics* **81**, 559-575 (2007).

R Core Team. R: A language and environment for statistical computing. R Foundation for Statistical Computing, Vienna, Austria. (2020)

Rastas, P. Lep-MAP3: robust linkage mapping even for low-coverage whole genome sequencing data. *Bioinformatics* **33**, 3726-3732 (2017).

Roberts, A., Trapnell, C., Donaghey, J., Rinn, J. L. & Pachter, L. Improving RNA-Seq expression estimates by correcting for fragment bias. *Genome Biology* **12**, R22 (2011).

Roy Choudhury, S., Roy, S., Singh, S. K. & Sengupta, D. N. Molecular characterization and differential expression of β-1,3-glucanase during ripening in banana fruit in response to ethylene, auxin, ABA, wounding, cold and light–dark cycles. *Plant Cell Reports* **29**, 813-828 (2010).

Sessegolo, C., Burlet, N. & Haudry, A. Strong phylogenetic inertia on genome size and transposable element content among 26 species of flies. *Biology Letters* **12**, 20160407 (2016).

Shan, T., Pang, S., Li, J., Li, X. & Su, L. Construction of a high-density genetic map and mapping of a sex-linked locus for the brown alga *Undaria pinnatifida* (Phaeophyceae) based on large scale marker development by specific length amplified fragment (SLAF) sequencing. *BMC Genomics* **16**, (2015).

Shan, T. *et al.* First Genome of the Brown Alga *Undaria pinnatifida*: Chromosome-Level Assembly Using PacBio and Hi-C Technologies. *Frontiers in Genetics* **11**, (2020).

Simão, F. A., Waterhouse, R. M., Ioannidis, P., Kriventseva, E. V. & Zdobnov, E. M. BUSCO: assessing genome assembly and annotation completeness with single-copy orthologs. *Bioinformatics* **31**, 3210-3212 (2015).

Slater, G. S. C. & Birney, E. Automated generation of heuristics for biological sequence comparison. *BMC Bioinformatics* **6**, 31 (2005).

Templeton, G. F. A Two-Step Approach for Transforming Continuous Variables to Normal: Implications and Recommendations for IS Research. *Communications of the Association for Information Systems* **28**, (2011).

Trapnell, C. *et al.* Transcript assembly and quantification by RNA-Seq reveals unannotated transcripts and isoform switching during cell differentiation. *Nature Biotechnology* **28**, 511-515 (2010).

Van der Auwera, G. A. *et al.* From FastQ Data to High-Confidence Variant Calls: The Genome Analysis Toolkit Best Practices Pipeline: The Genome Analysis Toolkit Best Practices Pipeline. in *Current Protocols in Bioinformatics* (eds. Bateman, A., Pearson, W. R., Stein, L. D., Stormo, G. D. & Yates, J. R.) 11.10.1-11.10.33 (John Wiley & Sons, Inc., 2013).

Vera, J., Castro, J., Gonzalez, A. & Moenne, A. Seaweed Polysaccharides and Derived Oligosaccharides Stimulate Defense Responses and Protection Against Pathogens in Plants. *Marine Drugs* **9**, 2514-2525 (2011).

Wang, Y. *et al.* MCScanX: a toolkit for detection and evolutionary analysis of gene synteny and collinearity. *Nucleic Acids Research* **40**, e49-e49 (2012).

Wessel, P., Smith, W. H. F., Scharroo, R., Luis, J. & Wobbe, F. Generic mapping tools: improved version released. *Eos, Transactions American Geophysical Union* **94**, 409-410 (2013).

Yabu, H., Yasui, H. & Notoya, M. Chromosome numbers of *Undaria pinnatifida* f. *distans*. Bulletin of the Faculty of Fisheries, Hokkaido University **39**: 6-1 3. (1988).

Ye, N. *et al. Saccharina* genomes provide novel insight into kelp biology. *Nature Communications* **6**, (2015).

Zambounis, A., Elias, M., Sterck, L., Maumus, F. & Gachon, C. M. M. Highly Dynamic Exon Shuffling in Candidate Pathogen Receptors ... What if Brown Algae Were Capable of Adaptive Immunity? *Molecular Biology and Evolution* **29**, 1263-1276 (2012).

Zhang, C., Dong, S.-S., Xu, J.-Y., He, W.-M. & Yang, T.-L. PopLDdecay: a fast and effective tool for linkage disequilibrium decay analysis based on variant call format files. *Bioinformatics* **35**, 1786-1788 (2019).

Zhang, Y. *et al.* The complete chloroplast genome of Wakame (*Undaria pinnatifida*), an important economic macroalga of the family Alariaceae. *Mitochondrial DNA Part B* **1**, 25-26 (2016).
