# Supplementary material for: A genome-wide investigation of the effect of farming and human-mediated introduction on the ubiquitous seaweed Undaria pinnatifida
Source: Nat Ecol Evol. 2021 Jan 25;5(3):360–8. doi: 10.1038/s41559-020-01378-9 (PMC7929912; doi:10.1038/s41559-020-01378-9)
Supplement: Supplementary file 2 — Reporting Summary [file 41559_2020_1378_MOESM2_ESM.pdf]

nature research

Corresponding author(s): Yoon Hwan Su

Last updated by author(s): Dec 2, 2020

Reporting Summary

Nature Research wishes to improve the reproducibility of the work that we publish. This form provides structure for consistency and transparency in reporting. For further information on Nature Research policies, see our Editorial Policies and the Editorial Policy Checklist.

Statistics

For all statistical analyses, confirm that the following items are present in the figure legend, table legend, main text, or Methods section.

n/a	Confirmed	
☐	☒	The exact sample size (*n*) for each experimental group/condition, given as a discrete number and unit of measurement
☐	☒	A statement on whether measurements were taken from distinct samples or whether the same sample was measured repeatedly
☐	☒	The statistical test(s) used AND whether they are one- or two-sided<br>*Only common tests should be described solely by name; describe more complex techniques in the Methods section.*
☒	☐	A description of all covariates tested
☐	☒	A description of any assumptions or corrections, such as tests of normality and adjustment for multiple comparisons
☐	☒	A full description of the statistical parameters including central tendency (e.g. means) or other basic estimates (e.g. regression coefficient) AND variation (e.g. standard deviation) or associated estimates of uncertainty (e.g. confidence intervals)
☐	☒	For null hypothesis testing, the test statistic (e.g. *F*, *t*, *r*) with confidence intervals, effect sizes, degrees of freedom and *P* value noted<br>*Give P values as exact values whenever suitable.*
☒	☐	For Bayesian analysis, information on the choice of priors and Markov chain Monte Carlo settings
☒	☐	For hierarchical and complex designs, identification of the appropriate level for tests and full reporting of outcomes
☒	☐	Estimates of effect sizes (e.g. Cohen's *d*, Pearson's *r*), indicating how they were calculated

Our web collection on statistics for biologists contains articles on many of the points above.

Software and code

Policy information about availability of computer code

Data collection	Genotype calling was performed following the GATK.3.8-0 HaplotypeCaller function with ploidy level 2 and default parameters. Individual genotypes were combined with the GenotypeGVCFs function of GATK.3.8-0. Variants were filtered to excluded variant loci with Fisher strand bias > 60, mapping quality < 40.0, coverage lower than 50 higher than 1500, a minimum allele frequency of 0.0365 and no missing genotyping, using a combination of plink v1.9 and vcftools v0.1.15. The variants dataset was annotated with SnpEff v4.3.
Data analysis	plink v1.9, vcftools v0.1.15, SnpEff v4.3, SNPhylo, R 3.6.3, LEA 2.8.0, circlize_0.4.8, ggplot2 3.1.0, stacks v1.48, Biobase 2.46.0, topGO 2.38.1, MCScanX, Orthofinder, MAFFT v6, IQ-Tree v1.6.9, dollop, Blast2GO, EVidenceModeler, Exonerate, Tophat2, Cufflink, PASA, RepeatMasker v4.0.7, RepeatModeler, Geneious v8.1.2, blastn, blastp, CANU, BWA, perl, BUSCO v.1.1, Lep-MAP3, Trimmomatic, bowtie-2 v2.2.6, SAMtools v1.5, picard v2.9.2, RSEM v1.3.3, GMT 5.4.5.

For manuscripts utilizing custom algorithms or software that are central to the research but not yet described in published literature, software must be made available to editors and reviewers. We strongly encourage code deposition in a community repository (e.g. GitHub). See the Nature Research guidelines for submitting code & software for further information.

Data

Policy information about availability of data

All manuscripts must include a data availability statement. This statement should provide the following information, where applicable:

- Accession codes, unique identifiers, or web links for publicly available datasets
- A list of figures that have associated raw data
- A description of any restrictions on data availability

The raw sequencing reads were deposited in the National Center for Biotechnology Information (NCBI) in the BioProject accession PRJNA646283. The assemblies, gene model and functional annotation were deposited in the Marine Genome Information Center (http://www.magic.re.kr/) under the accession MA00358.

Field-specific reporting

Please select the one below that is the best fit for your research. If you are not sure, read the appropriate sections before making your selection.

☐ Life sciences ☐ Behavioural & social sciences ☒ Ecological, evolutionary & environmental sciences

For a reference copy of the document with all sections, see nature.com/documents/nr-reporting-summary-flat.pdf

Ecological, evolutionary & environmental sciences study design

All studies must disclose on these points even when the disclosure is negative.

Study description	We present a genome-wide analysis of natural, cultivated and introduced populations of the kelp Undaria pinnatifda to elucidate human-driven evolutionary change.
Research sample	The research sample constituted of 41 individuals of Undaria pinnatifida collected from eight populations located in Korea, France and New Zealand.
Sampling strategy	No statistical methods were used to predetermine sample size. The sample size was determined based on available samples and budgetary constraints for genotyping.
Data collection	In each populations, 10 mature sporophytes of Undaria pinnatifida were collected. Attention was given to collect sporophytes separated from each other by at least 2m.
Timing and spatial scale	Each populations were visited one time.
Data exclusions	No individuals were excluded from the dataset.
Reproducibility	No experiments were conducted, so replication of experimental results is not relevant.
Randomization	This is not relevant as our study does not consider variable assignments or categories.
Blinding	Blinding was not relevant to this study, which is standard in the population genetics field.

Did the study involve field work? ☒ Yes ☐ No

Field work, collection and transport

Field conditions	The study involves populations located in Korea, France and New Zealand. The general environment
Location	In Korea, Tongyeong (34°50'07.9"N 128°24'01.5"E), Goseong (38°17'45.2"N 128°33'01.4"E), Wando (approx. 34°19'30.2"N 126°39'05.5"E).<br>In France, Thau lagoon (43°25'49.8"N 3°40'20.2"E), Roscoff (48°42'58.9"N 3°57'57.7"W).<br>In New Zealand, (41°17'23.2"S 174°47'23.4"E), Lyall Bay (41°20'05.0"S 174°47'33.3"E).
Access & import/export	Visited habitats were located in public areas and did not required any permits to be accessed. Dried seaweeds are not subjected to particular regulations and they were shipped with specific permits.
Disturbance	A limited number of individuals were sampled from each populations, leaving them virtually undisturbed.

Reporting for specific materials, systems and methods

We require information from authors about some types of materials, experimental systems and methods used in many studies. Here, indicate whether each material, system or method listed is relevant to your study. If you are not sure if a list item applies to your research, read the appropriate section before selecting a response.

Materials & experimental systems

n/a	Involved in the study
☒	☐ Antibodies
☒	☐ Eukaryotic cell lines
☒	☐ Palaeontology and archaeology
☐	☒ Animals and other organisms
☒	☐ Human research participants
☒	☐ Clinical data
☒	☐ Dual use research of concern

Methods

n/a	Involved in the study
☒	☐ ChIP-seq
☒	☐ Flow cytometry
☒	☐ MRI-based neuroimaging

Animals and other organisms

Policy information about studies involving animals; ARRIVE guidelines recommended for reporting animal research

Laboratory animals	The study did not involved laboratory animals.
Wild animals	The study did not involved wild animals.
Field-collected samples	The study did not involved experimental procedure using live samples collected from the field.
Ethics oversight	No ethical approval was required to collect and extract DNA from algal material.

Note that full information on the approval of the study protocol must also be provided in the manuscript.
