# Supplementary material for: A genome-wide investigation of the effect of farming and human-mediated introduction on the ubiquitous seaweed Undaria pinnatifida
Source: Nat Ecol Evol. 2021 Jan 25;5(3):360–8. doi: 10.1038/s41559-020-01378-9 (PMC7929912; doi:10.1038/s41559-020-01378-9)
Supplement: Supplementary file 3 — Peer review information [file 41559_2020_1378_MOESM3_ESM.pdf]

naturereseach

Peer Review Information

Journal: Nature Ecology & Evolution
Manuscript Title: A genome-wide investigation of the effect of farming and human-mediated introduction on the ubiquitous seaweed Undaria pinnatifida
Corresponding author name(s): Hwan Su Yoon

Editorial Notes:

Redactions – published data	Parts of this Peer Review File have been redacted as indicated to remove third-party material.

Reviewer Comments & Decisions:

Decision Letter, initial version:

1st September 2020

*Please ensure you delete the link to your author homepage in this e-mail if you wish to forward it to your co-authors.

Dear Dr Yoon,

Your manuscript entitled "A genome-wide investigation of the effect of farming and human-mediated introduction on the ubiquitous seaweed Undaria pinnatifida" has now been seen by 2 reviewers, whose comments are attached. The reviewers have raised a number of concerns which will need to be addressed before we can offer publication in Nature Ecology & Evolution. We will therefore need to see your responses to the criticisms raised and to some editorial concerns, along with a revised manuscript, before we can reach a final decision regarding publication.

We therefore invite you to revise your manuscript taking into account all reviewer and editor comments. Please highlight all changes in the manuscript text file.

We are committed to providing a fair and constructive peer-review process. Do not hesitate to contact us if there are specific requests from the reviewers that you believe are technically impossible or unlikely to yield a meaningful outcome.

When revising your manuscript:

* Include a “Response to reviewers” document detailing, point-by-point, how you addressed each reviewer comment. If no action was taken to address a point, you must provide a compelling argument. This response will be sent back to the reviewers along with the revised manuscript.

* If you have not done so already please begin to revise your manuscript so that it conforms to our Article format instructions at http://www.nature.com/natecolevol/info/final-submission. Refer also to any guidelines provided in this letter.

* Include a revised version of any required reporting checklist. It will be available to referees (and, potentially, statisticians) to aid in their evaluation if the manuscript goes back for peer review. A revised checklist is essential for re-review of the paper.

Please use the link below to submit your revised manuscript and related files:

[REDACTED]

Note: This URL links to your confidential home page and associated information about manuscripts you may have submitted, or that you are reviewing for us. If you wish to forward this email to co-authors, please delete the link to your homepage.

We hope to receive your revised manuscript within four to eight weeks. If you cannot send it within this time, please let us know. We will be happy to consider your revision so long as nothing similar has been accepted for publication at Nature Ecology & Evolution or published elsewhere.

Nature Ecology & Evolution is committed to improving transparency in authorship. As part of our efforts in this direction, we are now requesting that all authors identified as ‘corresponding author’ on published papers create and link their Open Researcher and Contributor Identifier (ORCID) with their account on the Manuscript Tracking System (MTS), prior to acceptance. ORCID helps the scientific community achieve unambiguous attribution of all scholarly contributions. You can create and link your ORCID from the home page of the MTS by clicking on ‘Modify my Springer Nature account’. For more information please visit please visit www.springernature.com/orcid.

Please do not hesitate to contact me if you have any questions or would like to discuss these revisions further.

We look forward to seeing the revised manuscript and thank you for the opportunity to review your work.

[REDACTED]

Reviewers' comments:

Reviewer #1 (Remarks to the Author):

The manuscript in general is quite interesting, it uses a novel system to explore the genomic

consequences of domestication and invasion as a system to look at the effect of human mediated evolution. The outstanding features of this manuscript is the dataset that was assembled and the quality of the genomes which provides an excellent way to look for insight into a comparison (domestic, invasive, and natural) that is infrequently explored topic, especially in marine systems. This comparison is quite novel and interesting, but while the manuscript contains no flaws that make it unpublishable the presentation could be greatly improved, for example

1) The figure captions don't match the figures
2) There is inconsistency in referencing of supplemental material
3) There is missing information in the text and analysis that are not shown in either the main or supplemental material (e.g. Fst, Tajima D).

There are also other papers that contain both domestic and invasive such as sunflower, sorghum, and rice on this topic that could be referenced.

Line 28-29: I don't understand the sentence," Genome wide analysis of domesticated species that have also been introduced to non-native geographic regions however do not exist." I recommend deleting it. Are you saying that there is a limited number of studies on the comparative genomic consequences of domestication and invasion?

Line 83-84: by "domesticated and introduced" do you mean "domesticated and invasive", this seems more appropriate as range expansion is a domestication syndrome trait.

Line 127-128: Does this sentence "The traditional repeat-rich heterochromatin and gene-rich euchromatin could not be clearly differentiated in Kr2015" mean that you did not find centromeres or does it mean that the organism has holocentric centromeres?

Line 150 – it seems unlikely that every individual had 853.77 GB of sequence, I suggest double checking this figure

Line 209: Why isn't the ROH, Pi and LD for the natural population presented?

Line 252: The natural population diversity is not presented; it needs to be.

Line 324: what are the 252 regions this line discusses?

Line 394: which sup table is Supplemental table X?

What is the generation time, do expect many generations between 1987-2017? This would inform the interpretation of the generational sampling.

In figure 2 the colors in the caption do not match the colors in the figure, in the figure yellow appears to be French, and red appears to be New Zealand, and green appears to be native range.

In figure 2 it seems unlikely PC2 and PC3 explain the exact same amount of variance 22.84%, this is likely a typo, check

In figure 3 what are the boxes in figure 3c they do not seem to make any sense, are you showing where LD decays to a certain point? usually this is displayed as LD decays to 0.2 at XXX kb, it looks like the decay of LD to half its maximum, but the value is displayed nowhere. Also there is no caption for figure 3d

Qualitatively diversity looks lower in the domestic and invasive than wild, however, there are no tests comparing diversity.

Figure 3b would be more informative if it were a line graph rather than a scatterplot, also it would be interesting to see the ratio of Pi cultivated / Pi wild and Pi invasive/Pi wild

It would be interesting to see Fst between the invasive, native, and domesticated

It would also be interesting to see Tajima's D displayed

What is the critical value for significance for DCMS? and since this analysis is heavily relied on in the paper than to displace pi in two different ways in figure 3.

Line 309: What is the domestication syndrome of this species? This could be elaborated on here. Knowing this would help make sense of and improve the discussion of the genes under selection.

It would be interesting to see the past effective population size (maybe using PSMC analysis)

Reviewer #2 (Remarks to the Author):

This manuscript addresses the question of how species domestication and the introduction of these domesticated species to non-native regions have shaped their genomes. The model species used in the MS was the seaweed Undaria pinnatifida. One of the most intriguing results was that the domesticated form of this species was characterized by a higher genetic diversity than natural populations. Surprisingly at first sight, but considering how domestication works for this species, not surprising after all. Algal farmers frequently mix the domesticated form with different natural populations leading to sustained high levels of genetic diversity in the domestic population whereas natural populations, due to limited dispersal and population bottlenecks (e.g. founder effects), appear to have a lower genetic diversity.

To me, this manuscript addresses an important issue in the field of conservation ecology. Most of the approaches used in conservation ecology do not apply genomics approaches to estimate extinction risk, which can be significant, though, if inbreeding dominates for many generations. This manuscript offers new insights into how a controlled introduction of genes into domesticated populations helps to sustain these populations for high productivity and resilience. It also offers to identify the genomic regions that are characterized by high recombination rates and low homozygosity.

The genome sequences used for these analyses appear to be of high quality and the comparative analysis is comprehensive. To me, though, the main weakness of this paper is that it barely dives into

the genomic regions under selection in different populations and how those might help to explain the adaptation and evolution of these populations under the given environmental/breeding constraints. Although there is a paragraph on regions under putative selection, it looks like a collection of genes and gene families. Thus, I have the following suggestions to make to improve this part. Show genomic region of elevated genetic diversity in coding regions. Figure 3e points to regions of elevated SNPs but the window size (250kb) is pretty large for identifying any interesting regions that stand out. This kind of analysis should be accompanied by dN/dS data based on the same sliding window across chromosomes and coding regions. I am not very familiar with DCMS but it seems to be too course-grained based on the results shown. This additional analysis potentially would narrow down interesting regions under selection and it would also potentially identify markers that can be used by breeders. Another suggestion I have would be to apply coalescence theory to trace alleles under selection back in time. This would certainly strengthen the statements about the history of the genomic landscape in cultivars vs wild populations (line 213 onwards). It seems RNAseq has not been conducted although cDNA-libraries were available for genome annotations. With RNAseq data, though, it could be tested how selection impacts the phenotypes and therefore contributes to the adaptation of the different U. pinnatifida populations in different regions of the world and under conditions of domestication. Maybe the authors have already generated some RNAseq datasets while their paper was under review? Include them if available.

********************END********************

Author Rebuttal to Initial comments

The authors thank the Editor and two anonymous referees for their advice and efforts to improve the manuscript.
We have addressed, to the best of our abilities, the comments as detailed in the point-by-point reply below and hope that the revised manuscript will now be acceptable for publication.

Reviewer #1 (Remarks to the Author):
The manuscript in general is quite interesting, it uses a novel system to explore the genomic consequences of domestication and invasion as a system to look at the effect of human mediated evolution. The outstanding features of this manuscript is the dataset that was assembled and the quality of the genomes which provides an excellent way to look for insight into a comparison (domestic, invasive, and natural) that is infrequently explored topic, especially in marine systems. This comparison is quite novel and interesting, but while the manuscript contains no flaws that make it unpublishable the presentation could be greatly improved, for example

1) The figure captions don't match the figures
2) There is inconsistency in referencing of supplemental material

We regret these errors that arose during the last round of manuscript formatting. We have carefully checked the captions and referencing of supplemental material and believe that we have corrected all errors.

3) There is missing information in the text and analysis that are not shown in either the main or supplemental material (e.g. Fst, Tajima D).

The missing information is now provided (as described below).

There are also other papers that contain both domestic and invasive such as sunflower, sorghum, and rice on this topic that could be referenced.
The referee is correct when citing these important case studies. However, they concerned cases of populations that have first escaped from domestication, and then became invasive. This succession (natural => domesticated => invasive) is an interesting evolutionary model, well-illustrated by the above-mentioned case studies, but this situation contrasts with our case studies. In the case of *Undaria pinnatifida*, it is important to note that the domestication process (natural => domesticated) and the introduction process (natural => introduced) are independent events. This is what we attempted to emphasize in the introduction: to our knowledge there is no other genome-wide study of a similar case. We added a sentence in the introduction to clarify this aspect of independent derivation of the introduced and domesticated populations of *Undaria pinnatifida*:
"This kelp presents the rare characteristic of being independently cultivated and introduced in four continents outside of its native range. This situation contrasts with other well-studied cases such as *Oryza*[33] and *Sorghum*[34] in which domestication preceded escape to the wild."

Line 28-29: I don't understand the sentence," Genome wide analysis of domesticated species that have also been introduced to non-native geographic regions however do not exist." I recommend deleting it. Are you saying that there is a limited number of studies on the comparative genomic consequences of domestication and invasion?
Similar to the above point, the importance of the *Undaria pinnatifida* model is the independent derivation of cultivated and introduced populations. To clarify and emphasize this point, we changed the sentence as follows:
"However, genome wide analysis of domestication and introduction within a single species has not previously been done."

Line 83-84: by "domesticated and introduced" do you mean "domesticated and invasive", this seems more appropriate as range expansion is a domestication syndrome trait.
As explained above, domestication has been largely independent of the introduction process, with perhaps an exception in northern Brittany (Voisin et al. 2015). We used the term "introduced" to point out this feature: individuals that have been recently introduced from their native range in diverse regions around the world, independently of any original aim to cultivate it. We agree that the usage of the term "invasive" is often used in the case of domesticated individuals escaping from crops or farms to establish populations in the wild. This is not the case here. In addition, the definition of an invasive species generally includes the notion that the species is harmful to ecosystems. However, outside of its native range, the effects of *Undaria pinnatifida* on ecosystems are not typical of what is expected for an invasive species (for instance, replacement of native competitors occupying its ecological niche). This absence of impact was notably reported in Epstein & Smale (2017; DOI: 10.1002/ece3.3430). We therefore chose to use the more neutral term "introduced" for *Undaria pinnatifida*. Furthermore, it is important to clarify that *Undaria pinnatifida* was only domesticated in its native range in Asia. Elsewhere in the world, despite some small-scale cultivation it has not been extensively domesticated. Therefore, the link between expansion and a domestication syndrome is unlikely, or difficult to address.

Line 127-128: Does this sentence "The traditional repeat-rich heterochromatin and gene-rich euchromatin could not be clearly differentiated in Kr2015" mean that you did not find centromeres or does it mean that the organism has holocentric centromeres?
We were not able to identify centromeres in our genome assembly. In the brown algae genomes reported thus far, centromeres have not been formally identified but there is no evidence that they are holocentric. In related species (e.g. diatoms; Diner et al., 2017 https://doi.org/10.1073/pnas.1700764114) centromeres were monocentric.
However, in this sentence we were pointing to the homogeneity of the distribution of repeats and genes in the genomes of brown algae, as opposed to the distribution observed in plant genomes. We added a clarification note concerning the centromeres as follows:
"The traditional gene-rich euchromatin, repeat-rich heterochromatin and centromeric regions could not be clearly differentiated in Kr2015[36-38]"

Line 150 – it seems unlikely that every individual had 853.77 GB of sequence, I suggest double checking this figure
The 853.77 Gb represent the entire data generated for the 41 individuals. The sentence was misleading and changed as follows:
"We generated a total of 853.77 Gb of cleaned-trimmed paired-end sequence from the 41 individuals (average 20.69 Gb per individual)."

Line 209: Why isn't the ROH, Pi and LD for the natural population presented?
Because the focus of our study was on the comparison of domesticated and introduced populations, to keep the text succinct, we did not present these results. But we agree that this information is missing for a full understanding of the three population categories. We thus added the description of the natural population.
"Natural populations were characterized by high genetic diversity (mean $\pi = 0.0044$; Figure 3a-3b; Supplementary Figure 9 and Supplementary Table 9), high recombination rates (LD half-maximum decay at 3.95 kb in Natural; Figure 3c) but relatively high homozygosity (Natural mean total ROH length = 180.9 Mb and average ROH length = 1.13 Mb; Figure 3d and Supplementary Table 10)."

Line 252: The natural population diversity is not presented; it needs to be.
We added the description of the natural population as described above. We also modified one sentence to make the comparison with cultivated populations more clear.
"The cultivated populations of *U. pinnatifida* in Korea, however, deviated from these predictions, with genetic diversity (mean $\pi = 0.0044$; Supplementary Table 9); and LD disequilibrium decay (LD half-maximum decay at 3.95 kb; Figure 3c) comparable to that of natural populations (Figures 3a-3c)."

Line 324: what are the 252 regions this line discusses?
These are the regions detected in the DCMS comparison between individuals in Wellington Harbor 1987 and Wellington Harbor 2017. Because the term "regions" can be misleading, we changed it to "genomic windows". We clarified the sentence as follows:
"In contrast to the cultivated vs. natural population comparisons, the analysis across 30 to 60 generations (ca. one to two generations per year[66]) in Wellington Harbour between 1987 and 2017 were not enriched in a particular biological function (Supplementary Note and Supplementary Table 13 and 14)."

Line 394: which sup table is Supplemental table X?
We apologize for this typo and changed the text as follows: "Supplementary Table 8".

What is the generation time, do expect many generations between 1987-2017? This would inform the interpretation of the generational sampling.
Undaria pinnatifida is a short-lived species with one to two generations per year according to the regions. Seawater temperature is the major abiotic factor regulating its life cycle: the macroscopic sporophytes grow in cold waters. In the native range strong variation of seawater temperatures is observed between the winter cool waters and

summer warm waters. This change is responsible for the annual life cycle. Therefore, in its native range there is one generation per year for *Undaria pinnatifida.*
In New Zealand, however, seawater temperatures do not show such abrupt changes between summer and winter. This allows *Undaria pinnatifida* populations in New Zealand to often produce two overlapping cohorts annually, as reported in Schiel & Thompson (2012; https://doi.org/10.1016/j.jembe.2012.07.023). Therefore, in New Zealand, there is one to two generations per year. Therefore, from 1987 to 2017, the expected number of generations ranges from 30 - 60 generations. This is now explained in the following sentence:
"In contrast to the cultivated vs. natural population comparisons, the analysis across 30 to 60 generations (ca. one to two generations per year[68]) in Wellington Harbour between 1987 and 2017 did not reveal the enrichment of a particular biological function (Supplementary Note and Supplementary Table 13 and 14)."
And we added the reference:
"68. Schiel, D. R. & Thompson, G. A. Demography and population biology of the invasive kelp *Undaria pinnatifida* on shallow reefs in southern New Zealand. *Journal of Experimental Marine Biology and Ecology* **434-435**, 25-33 (2012)."

In figure 2 the colors in the caption do not match the colors in the figure, in the figure yellow appears to be French, and red appears to be New Zealand, and green appears to be native range.
The caption referred to three boxes separating the three different type of population considered. To avoid confusion, we added a yellow box for the French populations and adjusted the caption as follows:
"Red box, natural populations introduced in New-Zealand (NZ), outside the native range: Lyall Bay (NZ), and Wellington Harbour (NZ) sampled in 1987 and 2017. Yellow box, natural populations introduced in France (Fr), outside the native range: Thau (Fr) and Roscoff (Fr)."

In figure 2 it seems unlikely PC2 and PC3 explain the exact same amount of variance 22.84%, this is likely a typo, check
Indeed, there was a typo in the script used for the plotting of the PCA. It has been fixed and the explained variance of the PC3 was found to be 12.13%. Figure 2 was corrected accordingly.

In figure 3 what are the boxes in figure 3c they do not seem to make any sense, are you showing where LD decays to a certain point? usually this is displayed as LD decays to 0.2 at XXX kb, it looks like the decay of LD to half its maximum, but the value is displayed nowhere. Also there is no caption for figure 3d
The four different lines indicate the distance at which the LD decay to one-half its maximum. We added the values on the x axis of figure 3c.
We completed and corrected the caption of figure 3 as follows: "(a) Violin plot of the genetic diversity estimated by π in non-overlapping 10 kb windows. (b) Manhattan plot of the genetic diversity (π) estimated in non-overlapping 250 kb windows for the natural populations (green), cultivated populations (blue), New Zealand populations (red) and French populations (yellow). Local polynomial regression fitting are shown on the plots.

(c) Linkage disequilibrium (LD) decay in the four different type of populations, with thin line indicating the distance at which LD is half of its maximum. (d) Run of homozygosity (ROH) in the 41 individuals. Natural populations, cultivated populations, New Zealand populations and France populations are shown in green, blue, red and yellow, respectively."

Qualitatively diversity looks lower in the domestic and invasive than wild, however, there are no tests comparing diversity.

Genetic diversity is indeed lower in the introduced (i.e. invasive) populations than in the natural (i.e. wild) populations. However, the genetic diversity in the cultivated (i.e. domestic) populations is comparable to that of natural populations. We agree that the comparison was "qualitative". In our opinion, a rigorous test for genome-wide comparison of genetic diversity between population categories would be a permutation test, in which the populations would be permuted based on their category (i.e. natural, cultivated, introduced). However, because our dataset only contains two replicates of each type of populations, such a permutation test is not applicable. This is the reason we chose not to formally test the differences. Note also that the Pi ratio (see below) may be more convincing of the difference in genetic diversity in the different populations.

Figure 3b would be more informative if it were a line graph rather than a scatterplot, also it would be interesting to see the ratio of Pi cultivated / Pi wild and i invasive/Pi wild

We have produced a graph line of the genetic diversity for the cultivated (blue) and natural (green) populations, but we think that a plot is less readable than a scatterplot.

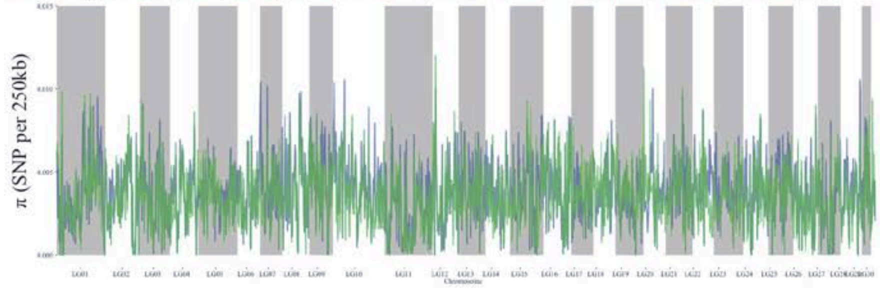

With this figure 3b, we aimed at showing that the base level of genetic diversity genome-wide was lower in the introduced populations (right plot) than in the natural and cultivated populations (left plot). In our opinion, the graph line does not help to emphasize this feature.

The comparison of the scatterplot of ratio of Pi cultivated/natural and Pi France/natural or New Zealand/natural demonstrates the lower level of genetic diversity in the introduced population and the comparable level of genetic diversity in the cultivated and natural populations. However, we think that this comparison remains clearer with the Manhattan plot showed in Figure 3b, now including regression lines to aid interpretation. We added the Pi ratio as Supplementary Figure 10.

It would be interesting to see Fst between the invasive, native, and domesticated

We also note that the Fst plot was missing from the original manuscript and contains important information, in particular, regarding the DCMS analysis, and to show the high temporal stability of the Wellington populations when compared to other comparisons among categories. Therefore we added a genome-wide graph line of the Fst for Cultivated-Natural, Wellington1987-Wellington2017, France-Natural and NewZealand2017-Natural. These scatterplots were included as Supplementary Figure 19.

It would also be interesting to see Tajima's D displayed
We also note that the TajimaD plot was missing from the original manuscript and contains important information, in particular, regarding the DCMS analysis. Therefore we added genome-wide plot of the TajimaD for Cultivated, Natural, France and NewZealand. These plots were included as Supplementary Figure 20.

What is the critical value for significance for DCMS? and since this analysis is heavily relied on in the paper than to displace pi in two different ways in figure 3.
As for any *p*-value, the value selected for significance is based on scientific precedent. We set a threshold for significance at $p = 0.025$ to determine the genomic windows under potential positive selection. This value was chosen over the traditional 0.05 one with the aim to be as conservative as possible.
Because the DCMS analysis was not properly presented in the original manuscript, we prepared a new figure (Figure 4) displaying the results of the DCMS analysis.

Line 309: What is the domestication syndrome of this species? This could be elaborated on here. Knowing this would help make sense of and improve the discussion of the genes under selection.
In Korean mariculture of *Undaria pinnatifida*, two phenomena are apparent: domestication of cultivars developed since the mid 2010's and cultivation of specimens generated by farmers. The cultivated individuals analyzed in our study were collected from a farm that did not exploit cultivars. We did not make phenotypic measurements of these individuals. In the first case, domestication syndrome might exist even though no formal phenotypic descriptions were published. In the second case, farming habits certainly prevent the emergence of a domestication syndrome sensu stricto. Indeed, farmers usually cross individuals cultivated in previous years and/or individuals collected in the wild, with particular phenotypes (e.g. large size), again not formally described. Phenotypic differences between natural and cultivated individuals can be observed but they are not fixed in any of the populations. Kelps have prodigious phenotypic plasticity, which could explain the difference observed between natural individuals growing on rocks in the intertidal zone and individuals growing on the surface of calm waters. Thus we can hypothesize (but only hypothesize) that one domestication syndrome might be large thallus and fast growing rates. However, in our opinion, the differences described above cannot be interpreted as a domestication syndrome, based on the lack of rigorous analyses of phenotypes in natural and cultivated populations.
For the readers to get some insights about the differences commonly observed between an individual from a natural population and one cultivated individual, we provided pictures in Supplementary Figure 15.

Furthermore, to avoid confusion and misleading the readers on which type of individuals were analyzed in our study we corrected the term "domestication" to "cultivation" when appropriate. We also modified the introduction as follows:
"During this period, farmers selected desired phenotypes and only recently breeding techniques were used to develop cultivars[26-28]"

It would be interesting to see the past effective population size (maybe using PSMC analysis)
Theoretically, the estimation of the past population size is of obvious interest to analyze the importance of potential bottlenecks in the cultivated populations or of founding events in the introduced populations. However, these events occurred only 70 years ago (beginning of the large scale cultivation in Korea in the 1950's) or even more recently (introduction in France in the late 1970's and in New Zealand in the 1980's).
Available methods to study past effective population size remain unreliable to measure recent variation in population size. The most commonly used programs (MSMC2 and PSMC) were shown to be informative for events that occurred as recently as 2,000 years ago for MSMC (Schiffels & Durbin, 2014; https://doi.org/10.1038/ng.3015) and 20,000 years ago for PSMC (Li & Durbin, 2011; 10.1038/nature10231). Even if the recently developed program SMC++ was shown to increase accuracy in recent times (Terhorst et al., 2017; 10.1038/ng.3748), events less than a 100-1000 years old are still extremely hard to estimate with confidence.
Furthermore, the coalescent theory involves multiple assumptions about the populations under study: neutral evolution and panmixia among the most important of these (Mather et al., 2019; https://doi.org/10.1002/ece3.5888). These assumptions are not met with *Undaria pinnatifida,* which is an autogamous species, thereby breaking the panmixia assumption, and likely affecting the reconstructed past effective population size. In addition, the genetic structure of the populations is known to have strong but not well understood effects on the results of these analyses. Furthermore, we showed strong genetic structure among populations in our study (Figure 2). For all of these reasons we chose to exclude such analyses. Nonetheless, they are provided here for this reviewer to inspect:

natureresearch

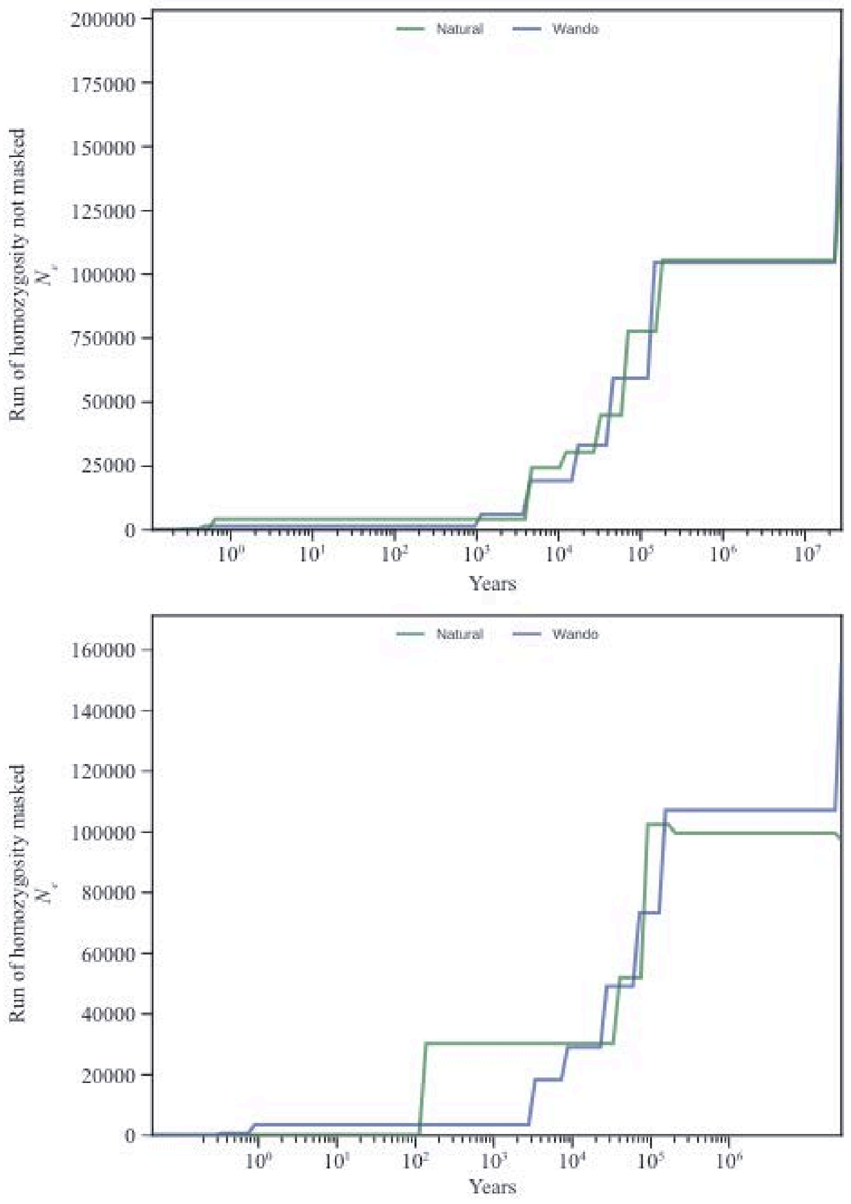

The figures show that while using all variable loci (upper figure) or variable loci outside of run of homozygosity (lower figure), estimation of population size of the cultivated and natural populations were not informative because the resolution was insufficient in the last 100 years. This supports the idea that even if we would leave aside our concerns regarding the bias and violation of the coalescent model, the reconstruction of ancient population size would not provide meaningful results for our study.

Reviewer #2 (Remarks to the Author):
This manuscript addresses the question of how species domestication and the introduction of these domesticated species to non-native regions have shaped their genomes. The model species used in the MS was the seaweed Undaria pinnatifida. One of the most intriguing results was that the domesticated form of this species was characterized by a higher genetic diversity than natural populations. Surprisingly at first sight, but considering how domestication works for this species, not surprising after all. Algal farmers frequently mix the domesticated form with different natural populations leading to sustained high levels of genetic diversity in the domestic population whereas natural populations, due to limited dispersal and population bottlenecks (e.g. founder effects), appear to have a lower genetic diversity.
To me, this manuscript addresses an important issue in the field of conservation ecology. Most of the approaches used in conservation ecology do not apply genomics approaches to estimate extinction risk, which can be significant, though, if inbreeding dominates for many generations. This manuscript offers new insights into how a controlled introduction of genes into domesticated populations helps to sustain these populations for high productivity and resilience. It also offers to identify the genomic regions that are characterized by high recombination rates and low homozygosity.
The genome sequences used for these analyses appear to be of high quality and the comparative analysis is comprehensive. To me, though, the main weakness of this paper is that it barely dives into the genomic regions under selection in different populations and how those might help to explain the adaptation and evolution of these populations under the given environmental/breeding constraints. Although there is a paragraph on regions under putative selection, it looks like a collection of genes and gene families.
The referee is correct when stating that in this section we essentially provide a catalog of genes of interest. This is due to the fact that we did not wish to overinterpret our results, and tried to be as conservative as possible, in particular, because the design of our study did not allow for in-depth analyses, and because the methods can be sensitive to bias as discussed in the manuscript. In particular, as shown in the new Figure 4, now cited in the discussion section, the gene density is high and adds up to the complexity of the population structure and history in the interpretation of the biological function that might be under selection. In most cases, it was not possible to discriminate between positive selection at a locus and genetic hitchhiking at the flanking loci, and thus did not allow us to more clearly define which genes could be potentially under positive selection. We hope that with this new figure (discussed in the relevant discussion section), the reviewer can now better understand our choice. Genome-wide association studies (GWAS) are needed to explore further this question, as indicated in the discussion with the following sentence:

"In the future, targeted sampling and an explicit experimental design are needed to better connect genetic and phenotypic information. In particular, quantitative trait loci (QTL) mapping in crosses between cultivars from breeding lines and natural individuals could help elucidate the domestication process in *U. pinnatifida*"

Thus, I have the following suggestions to make to improve this part. Show genomic region of elevated genetic diversity in coding regions. Figure 3e points to regions of elevated SNPs but the window size (250kb) is pretty large for identifying any interesting regions that stand out. This kind of analysis should be accompanied by dN/dS data based on the same sliding window across chromosomes and coding regions. I am not very familiar with DCMS but it seems to be too course-grained based on the results shown. This additional analysis potentially would narrow down interesting regions under selection and it would also potentially identify markers that can be used by breeders.
We thank the reviewer for this suggestion. However, to identify markers that could be used by breeders would require a dataset designed for this task. As pointed out in the discussion, we here aimed at providing first insights about putative genes and functions to better examine in dedicated studies like genome wide association and quantitative trait locus analyses, which should be conducted with genotyped and phenotyped individuals for breeding lines and from natural populations.
Analysis of the genetic diversity in small windows is included in the DCMS analysis because it incorporates the genetic diversity (π) calculated in 50kb windows across the genome. For this reason, we chose to use the DCMS. DCMS is not particularly coarse-grained because it does not rely on a single estimate or index but, rather, several at once, including high-Fst outlier SNPs often used for intra-specific studies and recently diverged populations. With this approach, we aimed at distinguishing regions which are showing the same trend regardless of method used (here, the three methods ROH, Fst and Pi), and thus providing more confidence about regions (and genes) putatively under selection.
We chose not to use the traditional dN/dS analysis because it has been shown to produce misleading results in the case of recently diverged populations (Kryazhimskiy & Plotkin, 2008; https://doi.org/10.1371/journal.pgen.1000304), due to polymorphisms, including shared polymorphisms due to gene flow. Following the request of this reviewer, we computed dN/dS in sliding windows of 50kb in the cultivated and natural populations. As shown for chromosome LG30 in the figure below, the two analyses are not congruent. Many regions were shown to have unexpectedly high dN/dS values when the DCMS analysis did not detect a signal of positive selection. We believe that this result comes from the fact that the natural and cultivated populations diverged less than 70 years ago, and with incomplete divergence (gene flow is still acting due to the farming practices). Therefore, the high dN/dS values observed likely result more from polymorphisms rather than differential allelic fixation.

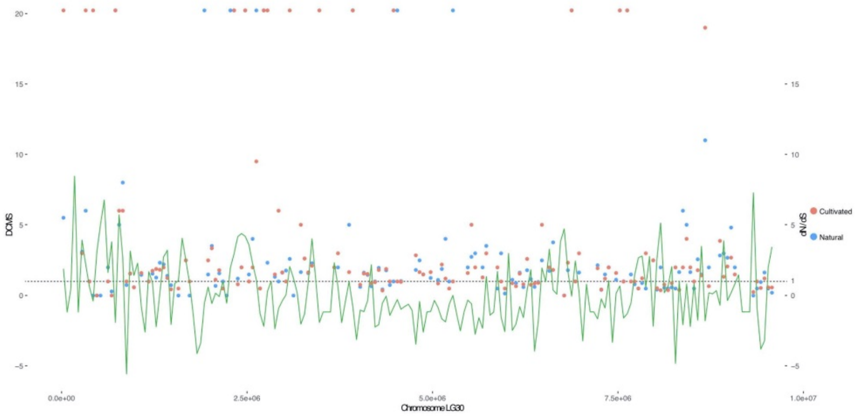

Another suggestion I have would be to apply coalescence theory to trace alleles under selection back in time. This would certainly strengthen the statements about the history of the genomic landscape in cultivars vs wild populations (line 213 onwards).
This is an interesting suggestion, but as we discussed above (reviewer #1 comment), using coalescent theory between genetically structured and demographically instable populations that have only diverged less than 70 years ago, is not possible.

It seems RNAseq has not been conducted although cDNA-libraries were available for genome annotations. With RNAseq data, though, it could be tested how selection impacts the phenotypes and therefore contributes to the adaptation of the different U. pinnatifida populations in different regions of the world and under conditions of domestication. Maybe the authors have already generated some RNAseq datasets while their paper was under review? Include them if available.
We agree that analysis of RNAseq data is necessary to further understand the impact of selection on the phenotypes of the domesticated *Undaria pinnatifida*. However, the transcriptome data generated for this manuscript was not designed to address these questions (we obtained RNA from 4 different tissues in one individual that were submitted to two different condition for 12 hours (i.e. in filtered and autoclaved seawater at 15C either under light or in the dark) before being fixed and the RNA extracted, resulting in eight sequencing libraries). Unfortunately, to generate a dataset suitable for the analysis proposed by this reviewer (RNAseq of genotyped and phenotyped natural individuals and breeding lines individuals) we would need to collect mature sporophytes, at best, in May 2021. With the time needed for sequencing and analysis of these individuals, our work would be pushed back at least one year, which we consider to be unwarranted.
Nevertheless, using the RNAseq generated for the annotated genes we compared orthologous groups containing genes under putative positive selection with those not in this category. This analysis revealed that in the majority of these families (94 out of 166) expression did not appear to be modified by positive selection. In the remaining 72 orthologous families, we observed both increased and decreased expression in the genes

under putative positive selection. The 79 genes with apparent differential expression and under putative positive selection encoded some of interest like a mannitol 1-phosphate dehydrogenase or two GDP-mannose-6 dehydrogenases. However, because these data were generated from a single individual we were not able to calculate DEGs and the observed differences in expression could also largely be attributed to differences between the tissues and treatments. Therefore, this analysis does not clearly respond to the reviewer's suggestion but is the best that could be done with the data at hand.
In conclusion, despite the absence of a robust gene expression analysis, we included the following text:

"Exploratory transcriptome analysis of genes within regions under positive selection revealed that they could potentially have different expression levels when compared to genes of similar functions encoded elsewhere in the genome (Supplementary Note; Supplementary Figure 16; Supplementary Table 12). However, these are preliminary results (Supplementary Note) and a more comprehensive transcriptomic analysis is needed to better understand the effect of positive selection on gene expression in the cultivated *U. pinnatifida*."
and in the Supplementary Material as follows:
"Using the RNA sequencing data generated for annotation of the Kr2015 genome (see 1-3), we investigated the expression of genes encoded in the genomic regions under putative positive selection. We first mapped the cleaned RNA reads for each of the eight libraries to the reference gene models using RSEM v1.3.3 (Li & Dewey, 2011) and the Transcripts Per Million (TPM) of each gene was estimated for each library. We then compared the expression level in the orthologous groups with at least one copy encoded in a genomic region under putative selection and one copy encoded outside of this region. Out of the 166 orthologous groups under consideration, 94 did not show an expression difference between the genes (Wilcoxon rank sum test p-value > 0.05). In the remaining 72 orthologous groups, expression appeared to be different between copy(ies) encoded in a genomic region under putative selection and copy(ies) encoded elsewhere on the genome (Wilcoxon rank sum test p-value < 0.05 (Supplementary Figure 16). This analysis only incorporated data obtained from a single individual and from different tissues submitted to different treatments (see 1-3), therefore do not represent a proper comparative analysis of gene expression. However, these results suggest that genes under positive selection might display expression differences when compared to neighbouring genes. A genome-wide association study and transcriptomic analysis should be conducted to clearly identify such loci and their effect on the phenotypes of *Undaria pinnatifida*."
and illustrated representatives cases in the Supplementary Figure 16.

Decision Letter, first revision:

13th November 2020

*Please ensure you delete the link to your author homepage in this e-mail if you wish to forward it to your co-authors.

Dear Dr Yoon,

Your manuscript entitled "A genome-wide investigation of the effect of farming and human-mediated introduction on the ubiquitous seaweed Undaria pinnatifida" has now been seen again by our reviewers, and in the light of their advice I am delighted to say that we can in principle offer to publish it. First, however, we would like you to revise your paper to address the final points made by the reviewers, and to ensure that it is as brief as possible and complies with our Guide to Authors at http://www.nature.com/natecolevol/info/final-submission.

TRANSPARENT PEER REVIEW
Nature Ecology & Evolution offers a transparent peer review option for new original research manuscripts submitted from 1st December 2019. We encourage increased transparency in peer review by publishing the reviewer comments, author rebuttal letters and editorial decision letters if the authors agree. Such peer review material is made available as a supplementary peer review file. Please state in the cover letter ‘I wish to participate in transparent peer review’ if you want to opt in, or ‘I do not wish to participate in transparent peer review’ if you don’t. Failure to state your preference will result in delays in accepting your manuscript for publication.
Please note: we allow redactions to authors’ rebuttal and reviewer comments in the interest of confidentiality. If you are concerned about the release of confidential data, please let us know specifically what information you would like to have removed. Please note that we cannot incorporate redactions for any other reasons. Reviewer names will be published in the peer review files if the reviewer signed the comments to authors, or if reviewers explicitly agree to release their name. For more information, please refer to our FAQ page.

SPECIFIC POINTS:
In particular, while checking through the manuscript and associated files, we noticed the following specific points which we will need you to address:

1. A brief editorial summary of the paper will appear on the journal homepage with the link to the paper. This is our proposed summary: ‘The genome of Pacific kelp, with data from natural, cultivated, and introduced populations, illustrates the combined influence of neutral (demography, migration) and non-neutral (selection) processes in human-driven evolutionary change.” Please let us know of any factual inaccuracies.
2. Please note that we have recently moved from having figures in the supplementary information to having them as Extended Data items, which are linked directly from the main text in the html version of the paper. You can have up to 10 Extended Data figures, and each may be multi-panel. All further figures should be compiled into a single Supplementary Informaton file that also contains the supplementary text and small supplementary tables. Larger tables can be submitted as spreadsheets.

Please see below for further details of how to submit supporting files.
3. Please complete the Editorial policy checklist and the Reporting Summary (links below) and upload them with your revised manuscript. We will publish the latter along with the paper. Please note that these forms are dynamic 'smart pdfs' and must therefore be downloaded and completed in Adobe Reader. Please also ensure that "Final Submission" box is checked.
a. Editorial policy checklist: https://www.nature.com/authors/policies/Policy.pdf
b. Reporting summary: https://www.nature.com/authors/policies/ReportingSummary.pdf

GENERAL POINTS:
We will also need you to check through all of the following general points when preparing the final version of your manuscript:

The main manuscript file should include the abstract, main text, methods, author contribution, data availability, code availability and competing interests statements, acknowledgements, references, and figure legends. Figures should be submitted separately as individual files. For details on other supporting material, please see below.

Title & Abstract:
Titles should give an idea of the main finding of the paper and ideally not exceed 90 characters (including spaces). We discourage the use of active verbs and do not allow punctuation.

The paper's abstract (about 150-200 words; no references) should serve both as a general introduction to the topic, and as a brief, non-technical summary of your main results and their implications. It should start by outlining the background to your work (why the topic is important) and the main question you have addressed (the specific problem that initiated your research), before going on to describe your new observations, main conclusions and their general implications. Because we hope that scientists across the wider ecology and evolution community will be interested in your work, the abstract should be as accessible as possible, explaining essential but specialised terms concisely. We suggest you show your abstract to colleagues in other fields to uncover any problematic concepts.

Figures:
Choosing the right electronic format for your figures at this stage will speed up the processing of your paper. We would like the figures to be supplied as vector files - EPS, PDF, AI or postscript (PS) file formats (not raster or bitmap files), preferably generated with vector-graphics software (Adobe Illustrator for example). Please try to ensure that all figures are non-flattened and fully editable. All images should be at least 300 dpi resolution (when figures are scaled to approximately the size that they are to be printed at) and in RGB colour format. Please do not submit Jpeg or flattened TIFF files. Please see our guidelines https://www.nature.com/documents/NRJs-guide-to-preparing-final-artwork.pdf for more details, and also our image policies http://www.nature.com/authors/editorial_policies/image.html.

We will edit your figures/tables electronically so they conform to Nature Ecology & Evolution style. If necessary, we will re-size figures to fit single or double column width. If your figures contain several parts, the parts should be labelled lower case a, b, and so on, and form a neat rectangle when assembled.

Figure legends must provide a brief description of the figure and the symbols used, within 350 words.

This must include definitions of any error bars employed in the figures.

Should your Article contain any items (figures, tables, images, videos or text boxes) that are the same as (or are adaptations of) items that have previously been published elsewhere and/or are owned by a third party, please note that it is your responsibility to obtain the right to use such items and to give proper attribution to the copyright holder. This includes pictures taken by professional photographers and images downloaded from the internet. If you do not hold the copyright for any such item (in whole or part) that is included in your paper, please complete and return this Third Party Rights Table, and attach any grant of rights that you have collected.

Please check the PDF of the whole paper and figures (on our manuscript tracking system) VERY CAREFULLY when you submit the revised manuscript. This will be used as the 'reference copy' to make sure no details (such as Greek letters or symbols) have gone missing during file-transfer/conversion and re-drawing.

Supporting Information:
All Supporting Information must be submitted in accordance with the instructions in the attached Inventory of Supporting Information, and should fit into one of two categories:

1. EXTENDED DATA: Extended Data are an integral part of the paper and only data that directly contribute to the main message should be presented. These figures will be integrated into the full-text HTML version of your paper and will be appended to the online PDF. There is a limit of 10 Extended Data figures, and each must be referred to in the main text, cited as Extended Data 1, Extended Data 2, etc. Each Extended Data figure should be of the same quality as the main figures, and should be supplied at a size that will allow both the figure and legend to be presented on a single A4 page. Each figure should be submitted as an individual .jpg, .tif or .eps file with a maximum size of 10 MB each. All Extended Data figure legends must be provided in the attached Inventory of Supporting Information, not in the figure files themselves.

2. SUPPLEMENTARY INFORMATION: Supplementary Information is material that is essential background to the study but which is not practical to include in the printed version of the paper (for example, video files, large data sets and calculations). Each item must be detailed in the attached Inventory of Supplementary Information. Tables containing large data sets should be in Excel format, with the table number and title included within the body of the table. All textual information and any additional Supplementary Figures (which should be presented with the legends directly below each figure) should be provided as a single, combined PDF. Please note that we cannot accept resupplies of Supplementary Information after the paper has been formally accepted unless there has been a critical scientific error.

Additional Supplementary Figures and other items are not required to be referred to in your manuscript text (though they can be), but should be numbered as Supplementary Figure 1, not SI1, etc.

Methods & Notes:
Please include references for the Methods in the same list as those for the main text, following on sequentially after the main text references. Any citations in the Supplementary Information will need inclusion in a separate SI reference list.

Please include a data availability statement as a separate section after Methods but before references, under the heading "Data Availability". This section should inform readers about the availability of the data used to support the conclusions of your study. This information includes accession codes to public repositories (data banks for protein, DNA or RNA sequences, microarray, proteomics data etc...), references to source data published alongside the paper, unique identifiers such as URLs to data repository entries, or data set DOIs, and any other statement about data availability. All data that support the findings of the study must be made available. If DOIs are provided, we also strongly encourage including these in the Reference list (authors, title, publisher (repository name), identifier, year). For more guidance on how to write this section please see:
http://www.nature.com/authors/policies/data/data-availability-statements-data-citations.pdf

Nature Research policies (https://www.nature.com/authors/policies/availability.html#data) include a strong preference for research data to be archived in public repositories and in some cases this is mandatory. If you need help complying with this policy, or need help depositing and curating your research data (including raw and processed data, text, video, audio and images) you should consider:

Contacting Springer Nature's Research Data Helpdesk
(https://www.springernature.com/gp/authors/research-data-policy/helpdesk/12327114) for advice.
Finding a suitable data repository (https://www.springernature.com/gp/authors/research-data-policy/repositories/12327124) for your data.
Uploading your data to Springer Nature's Research Data Support service
(https://springernaturedata.typeform.com/to/UeGGKT). Please note there are fees
(https://www.springernature.com/gp/authors/research-data-policy/pricing/15499842) for using Springer Nature's Research Data Support service.

Finally, we require authors to include a statement of their individual contributions to the paper, such as experimental work, project planning, data analysis, etc., immediately after the acknowledgements. The statement should be short, and refer to authors by their initials. For details please see the Authorship section of our joint Editorial policies at
http://www.nature.com/authors/editorial_policies/authorship.html

We will not send your revised paper for further review if, in the editors' judgement, the referees' comments on the present version have been addressed. If the revised paper is in Nature Ecology & Evolution format, in accessible style and of appropriate length, we shall accept it for publication immediately.

Please resubmit electronically

* the final version of the text (not including the figures) in either Word or Latex.

* publication-quality figures. For more details, please refer to our Figure Guidelines, which is available here: https://www.nature.com/documents/NRJs-guide-to-preparing-final-artwork.pdf .

* any Extended Data and Supplementary Information, as per instructed, with
the associated Inventory document.

* copies of our reporting and editorial policy checklists even if they have not changed since the previous round of revision.

* a point-by-point response to any issues raised by our reviewers and to any editorial suggestions.

* any suggestions for cover illustrations, which should be provided at high resolution as electronic files. Please note that such pictures should be selected more for their aesthetic appeal than for their scientific content. I am sure you will understand that we cannot make any promise as to whether any of your suggestions might be selected for the cover of Nature Ecology & Evolution.

Please use the following link to access your home page:

[REDACTED]

*This url links to your confidential homepage and associated information about manuscripts you may have submitted or be reviewing for us. If you wish to forward this e-mail to co-authors, please delete this link to your homepage first.

Please also send the following forms as a hand-signed PDF by email to ecoevo@nature.com.

*Please sign and return the Licence to Publish form

Or, if the corresponding author is a Crown government employee (including Great Britain and Northern Ireland, Canada and Australia), please sign
and return the Licence to Publish form for Crown government employees , or the Licence to Publish form for US government employees

For more information on our licence policy, please consult http://npg.nature.com/authors.

AUTHORSHIP

CONSORTIA -- For papers containing one or more consortia, all members of the consortium who contributed to the paper must be listed in the paper (i.e., print/online PDF). If necessary, individual authors can be listed in both the main author list and as a member of a consortium listed at the end of the paper. When submitting your revised manuscript via the online submission system, the consortium name should be entered as an author, together with the contact details of a nominated consortium representative. See https://www.nature.com/authors/policies/authorship.html for our authorship policy and https://www.nature.com/documents/nr-consortia-formatting.pdf for further consortia formatting guidelines, which should be adhered to prior to acceptance.

ORCID

Nature Ecology & Evolution is committed to improving transparency in authorship. As part of our efforts in this direction, we are now requesting that all authors identified as ‘corresponding author’ create and link their Open Researcher and Contributor Identifier (ORCID) with their account on the

Manuscript Tracking System (MTS) prior to acceptance. ORCID helps the scientific community achieve unambiguous attribution of all scholarly contributions. For more information please visit http://www.springernature.com/orcid

For all corresponding authors listed on the manuscript, please follow the instructions in the link below to link your ORCID to your account on our MTS before submitting the final version of the manuscript. If you do not yet have an ORCID you will be able to create one in minutes.
https://www.springernature.com/gp/researchers/orcid/orcid-for-nature-research

IMPORTANT: All authors identified as 'corresponding author' on the manuscript must follow these instructions. Non-corresponding authors do not have to link their ORCIDs but are encouraged to do so. Please note that it will not be possible to add/modify ORCIDs at proof. Thus, if they wish to have their ORCID added to the paper they must also follow the above procedure prior to acceptance.

To support ORCID's aims, we only allow a single ORCID identifier to be attached to one account. If you have any issues attaching an ORCID identifier to your MTS account, please contact the Platform Support Helpdesk.

We hope that you will support this initiative and supply the required information. Should you have any query or comments, please do not hesitate to contact me.

Nature Research journals encourage authors to share their step-by-step experimental protocols on a protocol sharing platform of their choice. Nature Research's Protocol Exchange is a free-to-use and open resource for protocols; protocols deposited in Protocol Exchange are citable and can be linked from the published article. More details can found at www.nature.com/protocolexchange/about.

We hope to hear from you within two weeks; please let us know if the revision process is likely to take longer.

[REDACTED]

****

Reviewer Comments:

Reviewer #1 (Remarks to the Author):

The authors have adequately addressed the reviewer comments, this is a solid paper. This is a study where the data is excellent and analysis well done, it is a nice contribution to the literature.

Figure 4 is particularly nice

Line 257-260: "The cultivated populations of U. pinnatifida in Korea, however, deviated from these predictions, with genetic diversity (mean $\pi = 0.0044$; Supplementary Table 9); and LD disequilibrium

decay (LD half-maximum decay at 3.95 kb; Figure 3c)" ----It seems unlikely that these number should be exactly the same in the cultivated and natural populations (line 197-199), the authors should double check these numbers.

Reviewer #2 (Remarks to the Author):

Thanks for addressing all of my suggestions. Well done. Congrats to this paper. ***[REDACTED]***

********************END********************

Author Rebuttal, first revision:

The authors thank the Editor and two referees for their advices and efforts to improve the manuscript.

Reviewer #1 (Remarks to the Author):
The authors have adequately addressed the reviewer comments, this is a solid paper. This is a study where the data is excellent and analysis well done, it is a nice contribution to the literature.

Figure 4 is particularly nice

We thank the reviewer for his kind comments and the help he provided to increase the quality of our study.

Line 257-260: "The cultivated populations of U. pinnatifida in Korea, however, deviated from these predictions, with genetic diversity (mean π = 0.0044; Supplementary Table 9); and LD disequilibrium decay (LD half-maximum decay at 3.95 kb; Figure 3c)" ----It seems unlikely that these number should be exactly the same in the cultivated and natural populations (line 197-199), the authors should double check these numbers.

We feel very sorry about this error that arose during the last formatting of our manuscript. The values in line 257-260 were corrected with correct values as follows:

"The cultivated populations of *U. pinnatifida* in Korea, however, deviated from these predictions, with genetic diversity (mean π = 0.0040; Supplementary Table 9); and LD disequilibrium decay (LD half-maximum decay at 3.14 kb; Figure 3c) comparable to that of natural populations (Figures 3a-3c)."

Reviewer #2 (Remarks to the Author):

Thanks for addressing all of my suggestions. Well done. Congrats to this paper. ***[REDACTED]***

We thank the reviewer for his kind comments and the help he provided to increase the quality of our study.

Final Decision Letter:

3rd December 2020

Dear Dr Yoon,

We are pleased to inform you that your Article entitled "A genome-wide investigation of the effect of farming and human-mediated introduction on the ubiquitous seaweed Undaria pinnatifida", has now been accepted for publication in Nature Ecology & Evolution.

Before your manuscript is typeset, we will edit the text to ensure it is intelligible to our wide readership and conforms to house style. We look particularly carefully at the titles of all papers to ensure that they are relatively brief and understandable.

The subeditor may send you the edited text for your approval. Once your manuscript is typeset you will receive a link to your electronic proof via email within 20 working days, with a request to make any corrections within 48 hours. If you have queries at any point during the production process then please contact the production team at rjsproduction@springernature.com. Once your paper has been scheduled for online publication, the Nature press office will be in touch to confirm the details.

Acceptance of your manuscript is conditional on all authors' agreement with our publication policies (see www.nature.com/authors/policies/index.html). In particular your manuscript must not be published elsewhere and there must be no announcement of the work to any media outlet until the publication date (the day on which it is uploaded onto our web site).

The Author's Accepted Manuscript (the accepted version of the manuscript as submitted by the author) may only be posted 6 months after the paper is published, consistent with our self-archiving embargo. Please note that the Author's Accepted Manuscript may not be released under a Creative Commons license. For Nature Research Terms of Reuse of archived manuscripts please see: http://www.nature.com/authors/policies/license.html#terms
If you have posted a preprint on any preprint server, please ensure that the preprint details are updated with a publication reference, including the DOI and a URL to the published version of the article on the journal website.

An online order form for reprints of your paper is available at <a

href="https://www.nature.com/reprints/author-reprints.html">https://www.nature.com/reprints/author-reprints.html. All co-authors, authors' institutions and authors' funding agencies can order reprints using the form appropriate to their geographical region.

We welcome the submission of potential cover material (including a short caption of around 40 words) related to your manuscript; suggestions should be sent to Nature Ecology & Evolution as electronic files (the image should be 300 dpi at 210 x 297 mm in either TIFF or JPEG format). Please note that such pictures should be selected more for their aesthetic appeal than for their scientific content, and that colour images work better than black and white or grayscale images. Please do not try to design a cover with the Nature Ecology & Evolution logo etc., and please do not submit composites of images related to your work. I am sure you will understand that we cannot make any promise as to whether any of your suggestions might be selected for the cover of the journal.

You can now use a single sign-on for all your accounts, view the status of all your manuscript submissions and reviews, access usage statistics for your published articles and download a record of your refereeing activity for the Nature journals.

To assist our authors in disseminating their research to the broader community, our SharedIt initiative provides you with a unique shareable link that will allow anyone (with or without a subscription) to read the published article. Recipients of the link with a subscription will also be able to download and print the PDF.

You can generate the link yourself when you receive your article DOI by entering it here: <a href="http://authors.springernature.com/share">http://authors.springernature.com/share<a>.

[REDACTED]

P.S. Click on the following link if you would like to recommend Nature Ecology & Evolution to your librarian http://www.nature.com/subscriptions/recommend.html#forms

** Visit the Springer Nature Editorial and Publishing website at www.springernature.com/editorial-and-publishing-jobs for more information about our career opportunities. If you have any questions please click here.**
